# Breakpoint Chlorination Chemistry in a Chlorine-Cyanurate System and Trade-Offs between Nitrosamine Formation and Micropollutant Removals

**DOI:** 10.1021/acs.est.5c09618

**Published:** 2025-11-18

**Authors:** Yi-Hsueh Chuang, Chia-Shun Chou, Yi-Lin Chu, Yen-Pao Chiang

**Affiliations:** Institute of Environmental Engineering, 34914 National Yang Ming Chiao Tung University No. 1001, University Rd., Hsinchu city 30010, Taiwan

**Keywords:** cyanuric acid, kinetic model, dichloroisocyanurate, reactive radicals, trichloramine

## Abstract

Breakpoint chlorination occurs in swimming pools and generates •OH and nitrosating agents. While •OH facilitates micropollutant removal, nitrosating agents promote nitrosamine formation. Cyanuric acid, a common chlorine stabilizer, reacts reversibly with free chlorine to form chlorinated cyanurates, effectively “locking” free chlorine and altering breakpoint chemistry. Accurate estimation of free chlorine is essential for evaluating these impacts and depends on the hydrolytic dissociation constants of chlorinated cyanurates, yet reported values vary widely. This study re-evaluates these constants and examines how cyanuric acid influences breakpoint reactions, focusing on the trade-off between •OH-driven micropollutant degradation and nitrosamine formation. Using phenolic probes, we show that electrochemically determined hydrolytic dissociation constants more accurately predict free chlorine concentrations under pool-relevant conditions than spectrophotometric values. Kinetic experiments reveal that chlorinated cyanurates participate in breakpoint reactions, chlorinating NH_2_Cl and NHCl_2_ with rate constants approximately half of those of HOCl. Cyanuric acid also catalyzes NHCl_2_ formation from NH_2_Cl. Under simulated pool conditions, cyanuric acid enhanced micropollutant removal, suppressed NCl_3_ formation, but promoted nitrosamine formation. A refined kinetic model captured these trends and provided mechanistic insights. Cyanuric acid, while mitigating scavenging of •OH and nitrosating agents by oxidants, prolongs NCl_3_–NHCl_2_ interactions, thereby increasing •OH and nitrosating agent yields while lowering residual NCl_3_.

## Graphical abstract

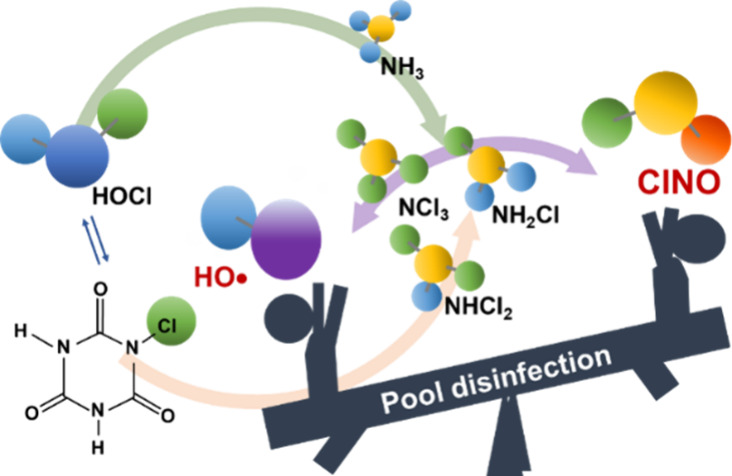

## Introduction

Chlorine disinfection remains the standard practice for maintaining microbial safety in swimming pools. In both outdoor and indoor pools, cyanuric acid is often present either as a deliberately added stabilizer to reduce chlorine photodegradation or as a byproduct of stabilized chlorine disinfectants such as trichloroisocyanuric acid and sodium dichloroisocyanurate.^1–3^ Typical cyanuric acid concentrations in swimming pools range from 39 to 595 μM, with a median of 311 μM.^4^ Cyanuric acid forms reversible complexes with free chlorine species (i.e., HOCl and OCl^–^), resulting in a dynamic equilibrium among free chlorine, cyanuric acid, and chlorinated cyanurates (Scheme 1). This equilibrium is influenced by pH, total chlorine concentration, and the cyanuric acid-to-chlorine molar ratio, with higher ratios favoring the formation of chlorinated cyanurates.^3^ Prior studies have reported that elevated cyanuric acid concentrations reduce disinfection efficacy attributed to a decline in HOCl.^3,5^ Given that HOCl is the principal oxidant responsible for chlorination of cellular and organic targets, these findings also suggest that chlorinated cyanurates are significantly less reactive as chlorinating agents.

**Scheme 1:**
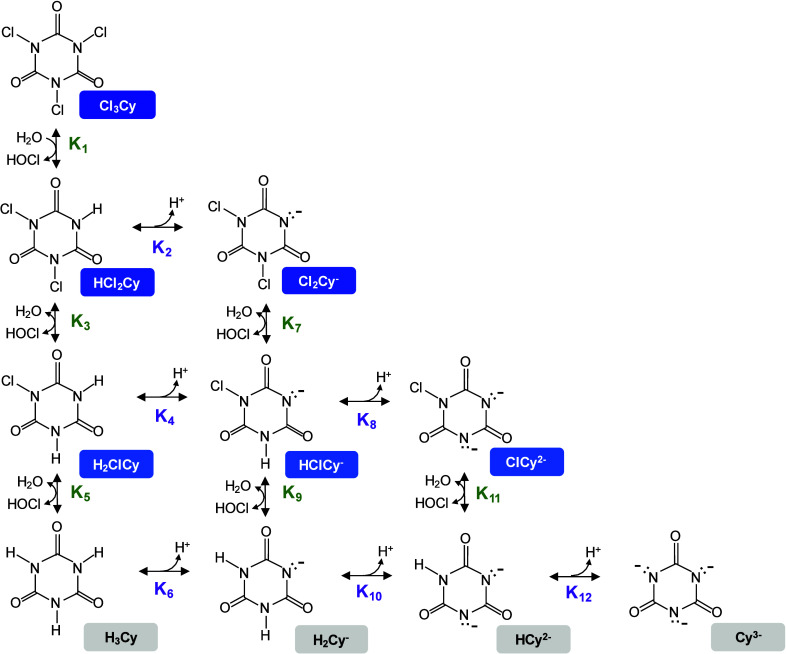
Equilibria among Free Chlorine, Chlorinated Cyanurates, and Cyanurates in a Chlorine-Cyanuric Acid System^a^

a The hydrolytic dissociation constants (K_1_, K_3_, K_5_, K_7_, K_9_, and K_11_) represent reactions that release HOCl, while the acid dissociation constants (K_2_, K_4_, K_6_, K_8_, K_10_, and K_12_) correspond to the dissociation of acidic species (values provided in Table S4).

While chlorination effectively mitigates acute risks of waterborne diseases, it raises chronic exposure concerns attributed to formation of disinfection byproducts (DBPs). Over 100 DBPs have been detected in pool waters.^6,7^ Among them, *N*-nitrosamines like *N*-nitrosodimethylamine (NDMA) occur at relatively low levels but carry significant risks due to their high toxicity. For example, Walse and Mitch reported NDMA levels ranging from 12–44 ng/L in indoor pools and 5–7 ng/L in outdoor pools, whereas a field study in Korea found a wider range of 0.7–210 ng/L in indoor pools.^8,9^

In contrast to drinking water, swimming pool water typically contains far higher concentrations of DBP precursors because of human-derived substances such as sweat and urine, resulting in DBP concentrations up to 200 times higher than those in tap water.^10–13^ Swimmers are estimated to release 78–250 mL of body fluids (mixtures of sweat and urine) during a swim event, equivalent of 1,400 mg-C of dissolved organic carbon (DOC).^11^ These excretions also introduce micropollutants such as pharmaceuticals and personal care products in addition to DBP precursors.^14^ Swimmers may be exposed to DBPs or micropollutants through inhalation, dermal absorption, and accidental ingestion.^15^

Since ammonia is a major component of human body excretions, release of body fluids during swimming triggers breakpoint reactions in pools wherein free chlorine is steadily maintained. A cascade of reactions is initiated when the chlorine-to-ammonia (Cl_2_/NH_4_^+^) molar ratio exceeds ∼ 1.5, resulting in the stepwise formation of monochloramine (NH_2_Cl), dichloramine (NHCl_2_), and trichloramine (NCl_3_) through their interactions with HOCl (Scheme 2).^16^ Subsequently, NCl_3_ reacts with either NHCl_2_ or NH_2_Cl to form N_2_ and other byproducts (reaction U12 in Scheme 2), with this reaction predominantly contribute to total chlorine loss relative to other reactions. While kinetic models have been developed to describe these processes,^16–18^ recent studies have expanded this understanding by identifying highly reactive species such as nitrosyl chloride (ClNO; via U12b), a potent nitrosating agent, and •OH (via U12c) as additional products of breakpoint chlorination.^19,20^ •OH plays a beneficial role in degrading recalcitrant micropollutants, yet ClNO can drive the formation of toxic nitrosamines. Furthermore, recent work has shown that •OH and NCl_3_ enhance haloacetonitrile production, likely through NCl_3_-mediated nitrogen incorporation into hydroxylated moieties of natural organic matter.^21^ These results indicate that breakpoint chlorination can inadvertently accelerate the formation of nitrogenous DBPs, which exhibit cytotoxicity and genotoxicity orders of magnitude greater than trihalomethanes.^22^

**Scheme 2:**
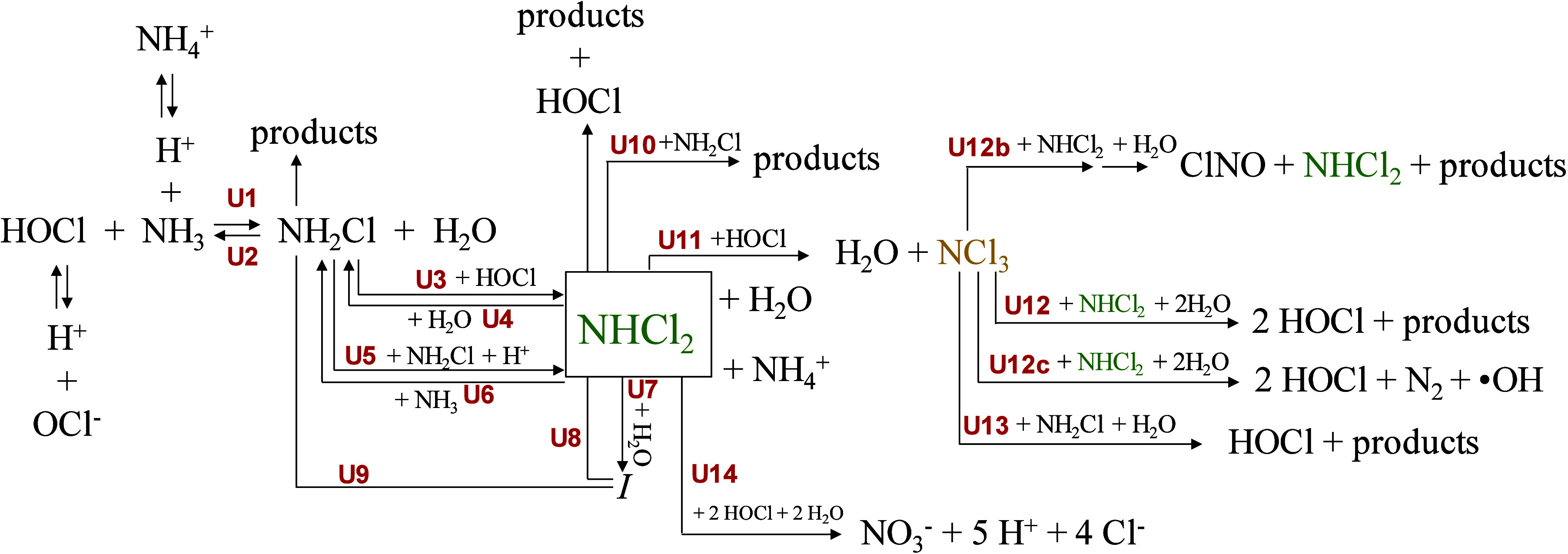
. Reaction Pathways during the Free Chlorine-Ammonia Interactions^a^

a The reaction scheme is based on Jafvert and Valentine’s model, with additional reactions regarding formation of nitrosating agent and •OH.^16,19,20^

The presence of cyanuric acid in pools complicates breakpoint chlorination chemistry in several ways. First, it reduces the equilibrium concentration of free chlorine, thereby lowering the transformation rates of ammonia and chloramines (U1, U3, and U11). Accurate quantification of free chlorine in HOCl–cyanuric acid systems is critical for evaluating the impact of cyanuric acid on the dynamics of breakpoint reactions. However, the widely used DPD (*N,N*-diethyl-p-phenylenediamine oxalate) method^23^ cannot distinguish free chlorine from chlorinated cyanurates, necessitating indirect estimation based on equilibrium and hydrolytic dissociation constants, particularly for dichloroisocyanurate (Cl_2_Cy^–^, K_7_) and hydrochloroisocyanurate (HClCy^–^, K_9_), which are dominant under typical pool conditions.^3,24–27^ Reported values for these constants, however, vary considerably. Early spectrometric measurements employing curve-resolving techniques reported p*K*_7_ and p*K*_9_ of 4.51 and 5.62, respectively.^25^ Later, Pinsky and Hu^26^ revised these values to 4.11 and 4.92 using linear sweep voltammetry to address potential spectral overlaps at mM concentrations. In a follow-up study, Jensen and Johnson^27^ suspected that interference from chlorinated cyanurates may have affected prior measurements and re-evaluated p*K*_9_ as 4.8 (nearly one log unit lower than previously reported^25^) using an amperometric membrane electrode. Despite these efforts, the debate over the accuracy of hydrolytic dissociation constants persists in recent studies,^3,28^ leaving the accuracy of p*K*_7_ and p*K*_9_ values an open question. While recognizing these disparities, a recent study argued in favor of the constants reported by the early spectrometric measurements, though without clearly articulating the underlying rationale.^3^ Notably, the U.S. EPA’s web-based Free Chlorine and Cyanuric Acid Simulator also employs equilibrium constants derived from these early spectrometric data.^29^

In addition to altering dynamics, cyanuric acid also appears to influence reaction pathways during breakpoint chlorination. One study indicates that cyanuric acid enhances the conversion of NH_2_Cl to NHCl_2_,^30^ suggesting the presence of additional pathways beyond direct HOCl-mediated transformation (U3) or NH_2_Cl disproportionation (U5). These pathways remain largely uncharacterized and are not yet represented in current kinetic models.

Moreover, cyanuric acid may influence the balance between contaminant degradation and byproduct formation during breakpoint reactions. Previous studies have shown that both micropollutant removal and nitrosamine formation peak at a Cl_2_/NH_4_^+^ molar ratio of approximately 1.8–2 (the ‘breakpoint’), then decline as the ratio increases.^19,20,31–34^ This trend, though no explanation was provided in previous works, may be attributed to enhanced scavenging of •OH and ClNO by excess free chlorine under postbreakpoint conditions. However, these scavenging reactions are likely diminished in the presence of cyanuric acid due to the lower reactivity of chlorinated cyanurates. For example, *k*_•OH_ for chlorinated cyanurates (<1.9 × 10^7^ M^–1^s^–1^)^35^ are orders of magnitude lower than that for HOCl/OCl^–^ (1.2–6 × 10^9^ M^–1^s^–1^).^36^ This reduced reactivity may extend the lifetime of •OH and ClNO, thereby enhancing removal of recalcitrant micropollutants while also elevating the risk of nitrosamine formation. These dual effects underscore the complex trade-offs introduced by cyanuric acid in chlorinated pool systems, which however remain largely uncharacterized.

The main objective of this study is to evaluate how cyanuric acid stabilizers affect micropollutant removal and DBP formation, particularly nitrosamines, under typical chlorinated pool conditions. To support this assessment, we first re-evaluated the equilibrium constants governing the hydrolytic dissociation of chlorinated cyanurates to improve the accuracy of free chlorine quantification. We then investigated potential interactions among cyanuric acid, chlorinated cyanurates, and chloramines, and determined their associated rate constants. Finally, we examined the trade-offs between nitrosamine formation and micropollutant removal during breakpoint chlorination and provided mechanistic insights using refined kinetic model simulations.

## Materials and Methods

### Chemicals and Reagents

All chemicals used in this study are listed in Table S1 in the Supporting Information (SI). All inorganic chloramine stock solutions were prepared freshly. NH_2_Cl stock solution was prepared by adding NaOCl dropwise to a NH_4_Cl solution stirred continuously at a 1:1 Cl_2_:N molar ratio.^37^ Stock solutions of NHCl_2_ were prepared by adjusting the pH of a 3.5 mM NH_2_Cl solution to 3.7 with 0.2 M phosphoric acid and maintaining this condition for 30 min. The solution was then stored in an ice bath for an additional hour to allow the NH_2_Cl disproportionation reaction (2 NH_2_Cl + H^+^ → NHCl_2_ + NH_4_^+^) to complete, leaving a solution containing NHCl_2_ and NH_4_^+^ at approximately a 1:1 molar ratio. The ammonia-containing NHCl_2_ stock solution was passed through an Amberlite IRC120 H resin to remove ammonia, yielding a final solution with a pH of 1.9 and minimal ammonia content (<5% molar ratio). Spectrophotometric analysis confirmed that the spectra (200–400 nm) for the resin-treated NHCl_2_ solution remained stable for over an hour; experiments associated with NHCl_2_ were conducted within 1 h. A Cary 60 UV–visible spectrophotometer was used to standardize the concentrations of inorganic chloramine stock solutions (Text S1).^38^

### Experiments for Re-evaluating Equilibrium Constants

Phenol and *p*-chlorophenol were used as probes for determining the equilibrium concentration of free chlorine in cyanuric acid-HOCl system. These compounds were chosen because their chlorination kinetics are well characterized,^39–42^ and their reaction pathways have been extensively studied.^40,41^ While both compounds undergo rapid and well-defined reactions with free chlorine, their reactivity is not identical (Table S5), making them complementary probes. Using both therefore provided a means to cross-validate the experimental results and ensured that the conclusions were not dependent on a single compound.

Experiments were carried out by treating phenol or *p*-chlorophenol at 2 μM with HOCl and cyanuric acid at target concentrations and at target pH. Samples were taken periodically with a stoichiometric amount of chlorine quencher (sodium thiosulfate). All experiments were conducted in duplicate.

### Experiments for Evaluating Interaction between Chloramines and (Chlorinated) Cyanurates

A total of 17 experiments were conducted to investigate the interaction between cyanuric acid or chlorinated cyanurates with NH_2_Cl or NHCl_2_ (as listed in Table S2). Experiments were carried out in a 10 cm path length quartz cuvette mounted in a spectrophotometer for *in situ* measurements of UV–vis spectra at wavelengths between 230–400 nm. A 10-mM or 20-mM phosphate buffer was used to maintain the pH throughout the experiments, and UV–vis spectra were taken periodically during the reaction (scan time was ∼ 1 s). An UV absorbance/simultaneous equations method was employed to evaluate oxidant evolution and decomposition during the reactions,^19^ as detailed in Text S1. Experiments were conducted in duplicate, and the spectra were highly consistent; for simplicity, only one representative spectrum is shown.

### Experiments for Evaluating Trade-Offs between Nitrosamine Formation and Micropollutant Removals

Experiments evaluating the trade-offs between micropollutant removals and nitrosamine formation in breakpoint reactions with or without cyanuric acid were carried out separately. Five model compounds (benzoate, nitrobenzene, 1,4-dioxane, *N, N*-Diethyl-meta-toluamide (DEET), and caffeine) that exhibit different reactivity toward •OH, •Cl, •Cl_2_^–^, •ClO, and reactive nitrogen species (Table S3),^43–53^ which are potentially generated in breakpoint reactions, were chosen for evaluating micropollutant removals. Their reactivity toward •OH fell within 2.5 × 10^9^–7.5 × 10^9^ M^–1^s^–1^. Among them, 1,4-dioxane showed relatively low reactivity with •Cl (*k* = 4.4 × 10^6^ M^–1^s^–1^), whereas nitrobenzene and caffeine exhibit the rate constants of 5.2 × 10^8^ and 3.9 × 10^10^ M^–1^s^–1^, respectively. In addition, caffeine is susceptible to •ClO attack, with a rate constant of 1.03 × 10^8^ M^–1^s^–1^. DEET was also included because it reacts appreciably with reactive nitrogen species (HOONO/OONO^–^, •NO, and •NO_2_^–^), with a combined rate constant on the order of 10^9^ M^–1^s^–1^. Our recent study demonstrates that •OH is the primary reactive species for their degradation in breakpoint reactions except caffeine.^20^

*N*-Chloro-dimethylamine (Cl-DMA) was utilized as a model precursor for nitrosamines as suggested by previous studies.^19,54^ Dimethylamine, excreted in human body fluids,^55^ is readily chlorinated to Cl-DMA under typical pool conditions.

Breakpoint chlorination experiments were conducted in deionized water buffered with phosphates at pH 7, using NaOCl and NH_4_^+^ at target Cl_2_/NH_4_^+^ molar ratios (ranging from 0 to up to 5) with micropollutants at sub-μM levels (0.2–0.5 μM) or with 7.5 μM Cl-DMA. A mixture of micropollutants or Cl-DMA and cyanuric acid in 10 mM phosphates was adjusted pH using NaOH or phosphoric acid. The mixture was then subsequently dosed with ammonia and HOCl at desired concentrations to initiate the reactions. Samples were collected periodically and immediately quenched with a stoichiometric amount of chlorine quencher. Two quenchers were used depending on the subsequent analysis: ascorbic acid was applied for NDMA samples, while sodium thiosulfate was used for micropollutant samples because ascorbic acid eluted early in the HPLC-UV chromatogram and slightly interfered with quantification. The pH change during the experiments was consistently <0.2 unit.

Reaction rate constant of ClNO with Cl-DMA was experimentally determined by monitoring the decay of Cl-DMA in a mixture of NO_2_^–^ and Cl^–^ at acidic pH. Additionally, a competition kinetics approach was employed to determine the rate constant of ClNO with H_3_Cy/H_2_Cy^–^ at pH 7 using Cl-DMA as a reference compound. Text S2 provides the experimental details.

### Analytical Method

Total chlorine concentration in the breakpoint chlorination experiments, with or without cyanuric acid, was measured by the DPD method. Nitrite, nitrate, and ammonia were analyzed using a Dionex Aquion ion chromatograph with a conductivity detector. NDMA, 1,4-dioxane, and DEET were analyzed using an Agilent GC (7890B)-MS (5977A). Nitrobenzene, benzoic acid, and caffeine were analyzed using an Agilent HPLC (1260 II) coupled with a UV detector. Analytical details are available in previous studies.^19,20^ Phenol, *p*-chlorophenol, and Cl-DMA were analyzed using an Agilent HPLC (1260 II) coupled with a UV detector (using 275 nm for phenol, 225 nm for *p*-chlorophenol, and 262 nm for Cl-DMA). Details for Cl-DMA analyses were provided in Text S2.

### Kinetic Modeling

A kinetic model encompassing 25 elementary reactions was adapted from our recent study (Reactions S1–S25 in Table S4).^20^ This model is based on the widely used unified (UF) model,^16^ with revised reactions and rate constants for NCl_3_–NHCl_2_ interactions.^19^ Scheme 2 provides the reactions. Additional revision was made for the bad reversible loop found in previous model, by revising the reversed reaction rates as suggested by a recent work.^56^ Moreover, this model was further refined with incorporating 24 reactions regarding the acid-dissociation reactions for (chlorinated) cyanuric acid/cyanurates, and six primary reactions between (chlorinated) cyanurates and NH_2_Cl or NHCl_2_ (Table 1), as discussed below. Kinetic modeling was implemented using Kintecus 6.8.^57^ Rate constants for the additional 24 reactions were either adapted from the literature, determined in this study, or reasonably assumed. For those parameters obtained through data fitting, optimization was performed sequentially rather than simultaneously, as elaborated in a later section.

**Table 1:** Primary Reactions and Rate Constants for the (Chlorinated) Cyanurates-Chloramines Interactions^a^

reaction		rate constant
H_3_Cy + NH_2_Cl → HClCy^–^ + NH_4_^+^	[R1]	*k*_R1_ = 2 M^–1^s^–1^
H_2_Cy^–^ + NH_2_Cl → HClCy^–^ + NH_3_	[R2]	*k*_R2_ < 0.01 M^–1^s^–1^
HClCy^–^ + NH_2_Cl → H_2_Cy^–^ + NHCl_2_	[R3]	*k*_R3_ = 166 M^–1^s^–1^
H_3_Cy + NHCl_2_ → H_2_ClCy + NH_2_Cl	[R4]	*k*_R4_ = negligible
H_2_Cy^–^ + NHCl_2_ → HClCy^–^ + NH_2_Cl	[R5]	*k*_R5_ = negligible
HClCy^–^ + NHCl_2_ → H_2_Cy^–^ + NCl_3_	[R6]	*k*_R6_ = 135 M^–1^s^–1^

a Reactions and rate constants were determined in this study.

## Results and Discussion

### Chemical Equilibrium Constants for Chlorine-Cyanuric Acid System

Initial experiments focused on evaluating the hydrolytic dissociation constants for Cl_2_Cy^–^ (K_7_ in Scheme 1) and HClCy^–^ (K_9_), driven by the fact that the wide variation in reported values introduces significant uncertainty in the predictions of free chlorine concentration at circumneutral pH (>5-fold differences; Text S3). To address this, we choose to examine the degradation kinetics of phenol in chlorine-cyanuric acid systems; phenol chlorination is one of the best-studied reactions, with established bimolecular rate constants well-established for HOCl/OCl^–^ reactions (Table S5).^40–42^

The degradation of phenol followed pseudo-first-order kinetics (Figure S6) due to the presence of free chlorine in substantial excess relative to phenol (i.e., 100 μM total Cl_2_ relative to the 2 μM phenol). In this case, the apparent rate constant for phenol degradation (*k*_app_) can be expressed by eq 1, where *f*_PhOH_ and *f*_PhO‑_ represent the fractions of total phenol in the conjugate acid (PhOH) and phenolate (PhO^–^) forms, respectively.

$$k_{\mathrm{app}}=k_{\mathrm{HOCl},\mathrm{PhO}-}{\left[\mathrm{HOCl}\right]}_{\mathrm{eq}}f_{\mathrm{PhO}-}+k_{\mathrm{HOCl},\mathrm{PhOH}}{\left[\mathrm{HOCl}\right]}_{\mathrm{eq}}f_{\mathrm{PhOH}}+\Sigma k_{\mathrm{Cl}-\mathrm{cyanurate},\mathrm{PhO}-}{\left[\mathrm{Cl}-\mathrm{cyanurate}\right]}_{\mathrm{eq}}f_{\mathrm{PhO}-}+\Sigma k_{\mathrm{Cl}-\mathrm{cyanurate},\mathrm{PhOH}}{\left[\mathrm{Cl}-\mathrm{cyanurate}\right]}_{\mathrm{eq}}f_{\mathrm{PhOH}}$$ 1

While *k*_HOCl, PhO‑_ are orders of magnitude larger than *k*_HOCl, PhOH_ (Table S5), experimental results in this study indicated that Cl-cyanurates exhibit negligible reactivity with phenol and phenolate (Figure S7). Furthermore, the reaction involving OCl^–^ is excluded due to its significantly lower reactivity.^41,58^ As a result, eq 1 simplifies to eq 2:

$$k_{\mathrm{app}}=k_{\mathrm{HOCl},\mathrm{PhO}-}{\left[\mathrm{HOCl}\right]}_{\mathrm{eq}}f_{\mathrm{PhO}-}+k_{\mathrm{HOCl},\mathrm{PhOH}}{\left[\mathrm{HOCl}\right]}_{\mathrm{eq}}f_{\mathrm{PhOH}}$$ 2

In this simplified form, *k*_app_ is solely dependent on the equilibrium concentration of HOCl ([HOCl]_eq_). The equilibrium constants K_7_ and K_9_ from various studies along with other relevant constants were used to calculate [HOCl]_eq_ and the corresponding *k*_app_ using eq 2. These calculated values were then compared with experimentally observed *k*_app_ to determine which set of K_7_ and K_9_ provided the best match.

Figures 1a–1b demonstrates that the values of p*K*_7_ (4.1) and p*K*_9_ (4.8) reported by Pinsky and Hu^26^ and Jensen and Johnson^27^ provided the most accurate *k*_app_ predictions from experiments with a variety of conditions, including those with different cyanuric acid concentration (0–300 μM) and different pH (6–8). The relative percentage errors remained within 15%, whereas predictions using p*K*_7_ and p*K*_9_ from other studies exhibited errors as high as 164%. Further validation using *p*-chlorophenol as a probe (*k*_HOCl_ and *k*_OCl‑_ provided in Table S5) also demonstrated a strong agreement between model predictions and experimental *k*_app_ with low errors (∼20%, Figure 1c). These findings support the accuracy of K_7_ and K_9_ values determined via electrochemical methods, and highlight the importance of refining these constants to enhance the robustness of equilibrium-based approaches for chlorine speciation.

**Figure 1:**
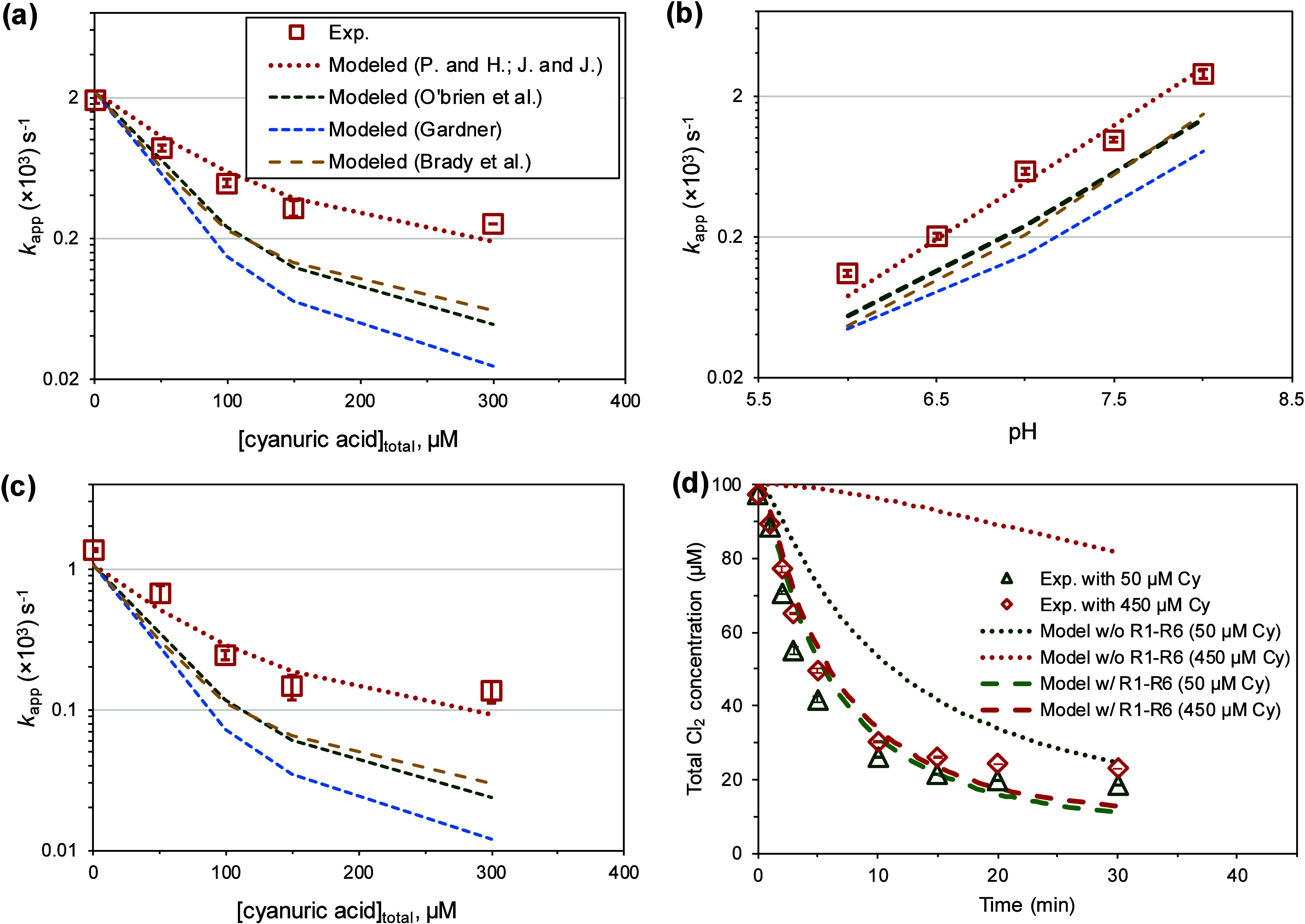
Experimentally observed *k* (*k*_app_) for the degradation of (a) phenol at pH 7 with different cyanuric acid concentration, (b) phenol at pH 6–8 with 100 μM cyanuric acid concentration, and (c) *p*-chlorophenol at pH 7 with different cyanuric acid concentration, and the predictions calculated using eq 2 in which the equilibrium concentration of HOCl were calculated using K_7_ and K_9_ reported from the literature. A 10-mM phosphate buffer was used to maintain the pH, with 2 μM phenol or *p*-chlorophenol treated by 100 μM HOCl and 0–300 μM cyanuric acid. The K_7_ and K_9_ reported from different works are summarized in Figure S4. References: P. and H.;^26^ J. and J.;^27^ O’Brien et al.;^25^ Gardner;^59^ Brady et al.^60^ (d) Experimental and modeled total chlorine concentrations during the reactions of 100 μM HOCl with 50 μM NH_4_^+^ and 50 or 450 μM cyanuric acid (Cy). Model simulations were implemented with and without inclusion of reactions R1–R6 from Table 1. Error bar represents data range from experimental duplicates.

### Development of Kinetic Model: Interactions between Cyanuric Acid and Chloramines

Kinetic models for evaluating micropollutant degradation and DBP formation need to reliably predict (1) radical formation and (2) the dynamic concentrations of oxidants during breakpoint chlorination in the presence of cyanuric acid. These predictions are essential to elucidate the competition among target contaminants, oxidants, and matrix components for reactions with these radicals. In this study, the chlorine-cyanuric acid reactions (Reactions S26–S49 in Table S4) were integrated into a previously developed breakpoint chlorination kinetic model.^20^ As the equilibrium constant (K) for an elementary reversible reaction is defined by the ratio of forward (*k*_f_) and reverse (*k*_r_) reaction rate constants (i.e., K = *k*_f_/*k*_r_), the knowledge of any one parameter enables the derivation of the others. Acid–base reactions are generally rapid, such that we assumed a 1 × 10^10^ M^–1^s^–1^ (diffusion-controlled limit) for the *k* values of base association reaction (e.g., H^+^ + ClCy^2–^ → HClCy^–^; *k* = 1 × 10^10^ M^–1^s^–1^). Although exceptions exist (e.g., nitronate anions),^61^ sensitivity analysis showed that varying these rate constants from 1 × 10^6^ to 1 × 10^10^ M^–1^s^–1^ had negligible effects on predicted chlorine loss (see later section).

However, the rate constants that have been reported involve reactions of nonchlorinated cyanuric acid species (H_2_Cy^–^ and HCy^2–^) with HOCl to form the corresponding monochlorocyanurates, with values of 7.27 × 10^4^ M^–1^s^–1^ and 2.16 × 10^7^ M^–1^s^–1^, respectively.^62^ By contrast, the kinetics of reactions between HOCl and chlorinated cyanurates (leading to higher chlorinated derivatives) remain largely unreported. We adopted a reasonable estimate of 2 × 10^5^ M^–1^s^–1^ for the remaining reactions between (chlorinated) cyanurates and HOCl. This estimate was based on findings by Jensen and Johnson,^63^ who used stopped-flow spectroscopy to determine that the reaction of DPD with HOCl (*k*_HOCl_ for DPD = 3.15 ± 0.03 × 10^6^ M^–1^s^–1^)^64^ had a reaction half-time of <0.4 s in HOCl-cyanuric acid systems. Our chosen rate constant (2 × 10^5^ M^–1^s^–1^) yielded a reaction half-time of 0.4 s under their experimental conditions (Text S4). When incorporating these rate constants, the kinetic model successfully predicted the degradation kinetics of phenol and *p*-chlorophenol in HOCl-cyanuric systems across all experimental conditions (Figure S9).

The kinetic model was employed to simulate total chlorine losses during breakpoint reactions. While the model accurately captured chlorine loss kinetics in the absence of cyanuric acid, it significantly underestimated the rate when cyanuric acid was present (Figure 1d and Figure S10). For instance, in experiments with 50 μM and 450 μM cyanuric acid, the measured total chlorine losses after 10 min were 74% and 70%, respectively, substantially higher than the model predictions of 46% and 5%. The discrepancy between the modeled and experimental results suggests that cyanuric acid or chlorinated cyanurates (particularly H_3_Cy, H_2_Cy^–^, and HClCy^–^, the dominant species under pool disinfection conditions) may actively facilitate the chlorination of ammonia, NH_2_Cl, or NHCl_2_ (i.e., Reactions R1–R6 in Table 1), counteracting the slow conversions due to the low equilibrium concentration of free chlorine.

Further experiments were carried out to investigate the interactions among HOCl, cyanuric acid, and chloramines. Spectral measurements of a mixture containing 400 μM cyanuric acid (in 10 mM phosphates at pH 7) treated with 50 μM NH_2_Cl showed that absorbance in the 278–330 nm range increased over time, while absorbance below 278 nm decreased (Figure 2). Since all cyanurate species and NH_2_Cl exhibit limited absorbance above 280 nm (Figure S1a), the emerging signal in the 278–330 nm range potentially indicates the formation of NHCl_2_ which has a characteristic absorbance peak at 292 nm (Figure S1a). The spectra also revealed an isosbestic point at 277 nm, where the molar absorption coefficient of NHCl_2_ (ε_NHCl2, 277 nm_ = 169 M^–1^cm^–1^) is twice that of NH_2_Cl (ε_NH2Cl, 277 nm_ = 84 M^–1^cm^–1^). This suggests that two NH_2_Cl molecules are consumed to generate one NHCl_2_ during the cyanuric acid-mediated NH_2_Cl conversion, leading to a total absorbance change at 277 nm (ΔA_277 nm_ = – 2×ε_NH2Cl, 277 nm_ + 1×ε_NHCl2, 277 nm_) of zero. Similar spectral patterns were obtained when repeating the experiments at pH 6, 6.5, and 7.5, where NH_2_Cl remains stable (<2% change) over a 20 min time scale (Figures S11–S13).

**Figure 2:**
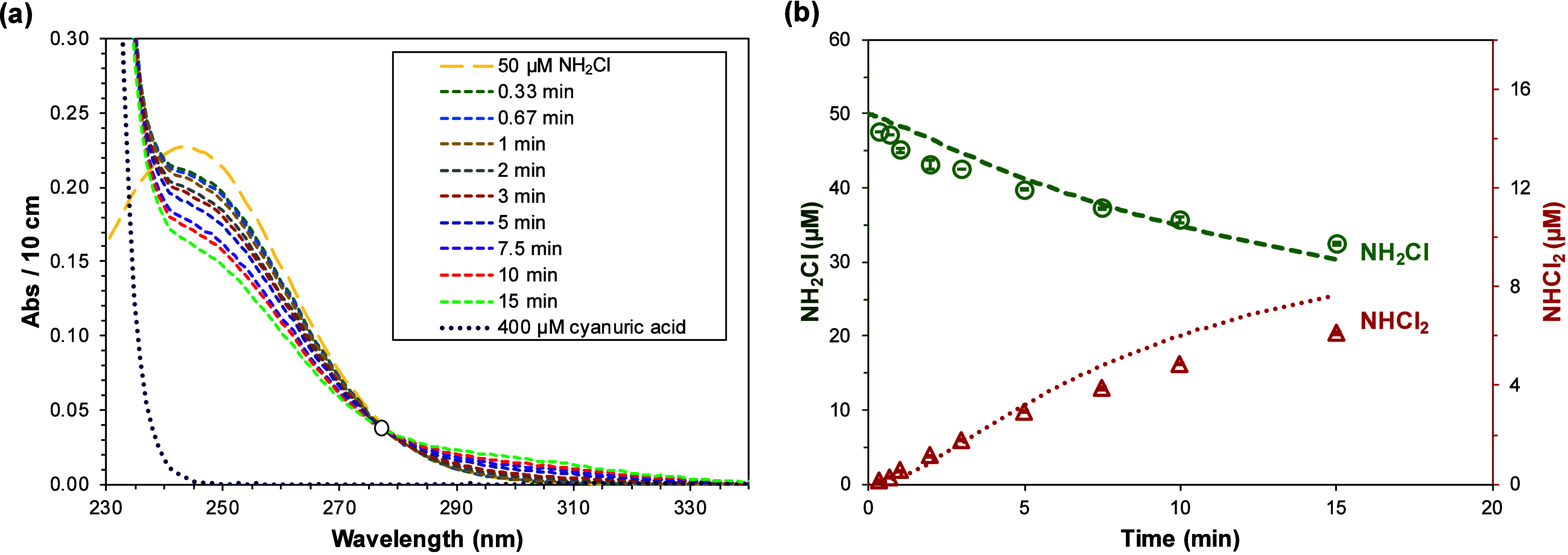
Time-dependent (a) UV spectra of a mixture containing 400 μM cyanuric acid in 10 mM phosphates at pH 7 treated with 50 μM NH_2_Cl, and (b) Concentrations of NH_2_Cl and NHCl_2_ over time, determined by the UV absorbance/simultaneous equations method. Symbols represent experimental data and lines are model predicted results. The hollow circle in (a) denotes the isosbestic point. Error bar represents data range from experimental duplicates.

A UV spectra/simultaneous-equation approach used to assess the evolution and decomposition of chloramines confirmed that NH_2_Cl decomposed with the concomitant formation of NHCl_2_ (Figure 2b and Figures S11b–S13b). Across the tested pH range of 6–7.5, the reaction followed a consistent stoichiometric ratio of 2.1(±0.1) NH_2_Cl moles depleted per mole NHCl_2_ formed, aligning with the isosbestic point at 277 nm observed in the spectra. The data further revealed that as pH increased from 6.0 to 7.5, both the decomposition of NH_2_Cl and the formation of NHCl_2_ slowed substantially. This trend points to the protonated form of cyanuric acid (H_3_Cy, p*K*_a_ = 6.88) being more effective than its deprotonated form (H_2_Cy^–^) in driving the conversion of NH_2_Cl to NHCl_2_. In line with this interpretation, kinetic analysis (Text S5) showed that NH_2_Cl decomposition in phosphate-buffered NH_2_Cl–cyanuric acid systems followed second-order kinetics, with apparent rate constants (*k*_app_) decreasing from 26.4 ± 0.1 M^–1^s^–1^ at pH 6.0 to 1.7 ± 0.1 M^–1^s^–1^ at pH 7.5.

Previous work by Valentine and Jafvert^65^ demonstrated that NH_2_Cl disproportionation to NHCl_2_ (NH_2_Cl + NH_2_Cl → NHCl_2_ + NH_3_; Reaction S5 in Table S4) proceeds via a general acid-catalyzed mechanism. In this pathway, protonation of NH_2_Cl produces NH_3_Cl^+^, which rapidly chlorinates a second NH_2_Cl to generate NHCl_2_, NH_3_, and H^+^. Accordingly, the overall second-order rate constant for NH_2_Cl disproportionation (i.e., *d*[NH_2_Cl]/*d*t = *k*_S5_×[NH_2_Cl]^2^) was expressed as the sum of contributions from proton-donating species. We extended this framework by recognizing that both H_3_Cy and H_2_Cy^–^ bear exchangeable protons and may therefore catalyze NH_2_Cl disproportionation. Incorporating these terms yields eq 3.

$$k_{S5}=k_{H+}\left[H^{+}\right]+k_{H3\mathrm{PO}4}\left[H_{3}{\mathrm{PO}}_{4}\right]+k_{H2\mathrm{PO}4-}\left[H_{2}{{\mathrm{PO}}_{4}}^{-}\right]+k_{H3\mathrm{Cy}}\left[H_{3}\mathrm{Cy}\right]+k_{H2\mathrm{Cy}-}\left[H_{2}{\mathrm{Cy}}^{-}\right]$$ 3

We estimated *k*_H3Cy_ and *k*_H2Cy‑_ using linear free-energy relationships which relate the specific rate constant of a proton donor to its p*K*_a_, the number of exchangeable protons, and the binding capacity of its conjugate base (Text S5).^65^ The resulting values, 0.37 M^–2^s^–1^ for H_3_Cy and 4.4 × 10^–4^ M^–2^s^–1^ for H_2_Cy^–^, were on par with those for H_2_CO_3_ (0.68 M^–2^s^–1^) and HCO_3_^–^ (1.7 × 10^–3^ M^–2^s^–1^). When the concentrations of each acid were substituted into the expanded expression, the calculated *k*_S5_ values (1.4 × 10^–3^–1.4 × 10^–2^ M^–1^s^–1^) were more than 3 orders of magnitude lower than the experimental *k*_app_ values obtained in the NH_2_Cl–cyanuric acid systems (1.7–26.4 M^–1^s^–1^). This large discrepancy indicates that acid-catalyzed disproportionation alone cannot explain the rapid NH_2_Cl decay observed in the presence of cyanuric acid.

To test this further, we performed control experiments in carbonate buffer (pH 7), where the H_2_CO_3_ concentration (173 μM) matched the H_3_Cy concentration present in NH_2_Cl–cyanuric acid experiments (50 μM NH_2_Cl and 400 μM cyanuric acid at pH 7). Under these conditions, NH_2_Cl remained essentially stable over 20 min (Figure S15), in stark contrast to the rapid decay observed in the presence of cyanuric acid. These findings demonstrate that cyanuric acid promotes NH_2_Cl conversion through additional pathways beyond simple acid catalysis.

Zhang and von Gunten’s^66^ recent investigations into chlorine reactions with amides demonstrate that OCl^–^ reacts with amides to form *N*-chloroamides through the formation of a stable complex. This structure features a hydrogen bond between the oxygen in OCl^–^ and the hydrogen in amide, enabling the amide N to attack the partially positively charged – Cl in OCl^–^. By analogy, the interaction between H_3_Cy and NH_2_Cl may proceed through the formation of a hydrogen bond between the nitrogen in NH_2_Cl and the hydrogen in H_3_Cy. This interaction facilitates a nucleophilic attack by the nitrogen in H_3_Cy on the chlorine in NH_2_Cl, generating H_2_ClCy and NH_3_ (Reaction R1 and Scheme S1a). Once formed, H_2_ClCy undergoes Cl­(+1) transfer reactions with another NH_2_Cl molecule to produce NHCl_2_ and regenerates cyanuric acid (Reaction R3 and Scheme S1b). These reactions follow an overall stoichiometry in which two NH_2_Cl molecules are consumed to produce one NHCl_2_ molecule. Cyanuric acid thus appears to act as a catalyst that promotes NH_2_Cl disproportionation and facilitates NHCl_2_ formation, mirroring with the catalytic role of amides in amides-OCl^–^ interactions.^66^

Our results suggest a two-step process in which H_3_Cy catalyzes the conversion of 2 NH_2_Cl to 1 NHCl_2_, though the rate-limiting step remains unknown. To investigate this, we treated a solution containing 20 μM HOCl pre-equilibrated with 400 μM cyanuric acid by 20–125 μM NH_2_Cl. The large excess of cyanuric acid relative to HOCl ensured that more than 96% of the chlorine remained cyanurate-bound, with HClCy^–^ accounting for 93% of the total Cl_2_. The results (Figure S16) revealed a biphasic pattern in both NHCl_2_ formation and NH_2_Cl decomposition, with an initial rapid phase lasting approximately 2 min followed by a slower phase. The kinetics of NHCl_2_ formation and NH_2_Cl decomposition were significantly enhanced in the HOCl-equilibrated cyanuric acid solution compared to a solution containing only cyanuric acid and NH_2_Cl during the first 2 min (Figure S17), suggesting Reaction R1 is the rate-determining step (i.e., *k*_R3_ > *k*_R1_). Therefore, this biphasic behavior arises from the interplay between a rapid reaction of HClCy^–^ with NH_2_Cl to form NHCl_2_ (R3) and a concurrent slower, cyanuric acid-facilitated transformation of NH_2_Cl to NHCl_2_ (R1–R3). The increasing stoichiometry of NH_2_Cl consumption per NHCl_2_ formed over time (Figure S16) supports the progressive contribution of both mechanisms.

Although NHCl_2_ may interact with H_3_Cy in a similar fashion to NH_2_Cl, our results suggest that H_3_Cy is less effective at catalyzing Cl­(+1) transfer between two NHCl_2_ molecules than between two NH_2_Cl molecules. When NHCl_2_ was treated with H_3_Cy alone at pH 7 for 5 min, only 10% decomposition was observed (Figure S18), which was half the 20% decomposition seen when NH_2_Cl was treated under the same conditions (Figure 2b). This can be explained by the relative electron-withdrawing nature of – Cl on NHCl_2_ compared to – H on NH_2_Cl, lowering the tendency of forming the hydrogen bond between the – N on NHCl_2_ and – H on H_3_Cy. However, NHCl_2_ decomposition was significantly accelerated in the 20 μM HOCl-equilibrated 400 μM cyanuric acid solution, reaching 70–80% within 5 min (Figure S19). While NCl_3_ is likely formed as an intermediate (R6), the newly formed NCl_3_ rapidly reacts with NHCl_2_, further accelerating NHCl_2_ decomposition (U12 in Scheme 2). As a result, the spectra did not display the characteristic absorption peak of NCl_3_ at 365 nm (Figure S19a).

### Rate Constants for Reactions between Cyanuric Acid and Chloramines

Plotting the initial decay rates for NH_2_Cl or NHCl_2_ (i.e., – d­[NH_2_Cl]/dt or – d­[NHCl_2_]/dt) against [NH_2_Cl]_initial_ (or [NHCl_2_]_initial_)×[HClCy^–^]_initial_ from all experiments yielded linear lines (Text S6 and Figure S20). Accordingly, the rate constants of *k*_R3_ (HClCy^–^ + NH_2_Cl) and *k*_R6_ (HClCy^–^ + NHCl_2_) were determined to be 166 and 135 M^–1^s^–1^, respectively. These rate constants are approximately half of those for HOCl with NH_2_Cl (278 M^–1^s^–1^) and NHCl_2_ (330 M^–1^s^–1^ at pH 7), reflecting that HClCy^–^ is less effective than HOCl in chlorine transfer reactions to chloramines.

These reactions were incorporated into the kinetic model to determine species-specific reaction rate constants for *k*_R1_ and *k*_R2_, by implementing the refined model to optimize *k*_5_ and *k*_6_ against experimental results for cyanuric acid-NH_2_Cl reaction at pH 6–7.5 (Text S7). The results showed that the rate constant for reaction of NH_2_Cl with H_3_Cy (*k*_R1_ = 2 M^–1^s^–1^) or H_2_Cy^–^ (*k*_R2_ < 0.01 M^–1^s^–1^) is several orders of magnitude lower than that for the reaction between HOCl with H_2_Cy^–^ (7.3 × 10^4^ M^–1^s^–1^).^62^

When incorporating Reactions R1–R6 and the respective rate constants, the refined kinetic model successfully predicted NH_2_Cl and NHCl_2_ concentrations across all HOCl–cyanuric acid–chloramine experiments listed in Table S2 (as shown in Figure 2b and Figure S11–S13, S16, and S21), with most deviations between predicted and measured values remaining within twice the method detection limit (2 × MDL ∼ 1.6 μM). Furthermore, the model significantly improved its ability to predict the total chlorine losses (relative errors <5%; Figure 1d and Figure S10), emphasizing the critical role of chlorinated cyanurates in the chlorination of ammonia, NH_2_Cl, and NHCl_2_. Our results also suggest that chlorinated cyanurates are more selective chlorination agents than HOCl, as they showed negligible reactivity with phenols despite their similar molecular weights to NH_2_Cl/NHCl_2_.

### Impact of Cyanuric Acid in Model Micropollutant Removals and DBP Formation

In the absence of cyanuric acid, the degradation of the five HOCl-resistant micropollutants during breakpoint chlorination followed a distinct volcano-shaped trend. Micropollutant removals increased with rising Cl_2_/NH_4_^+^ molar ratios, peaking around the breakpoint (1.8–2.0) before tapering off at higher ratios (Figure 3 and Figure S22), consistent with prior studies.^31,33,67^

**Figure 3:**
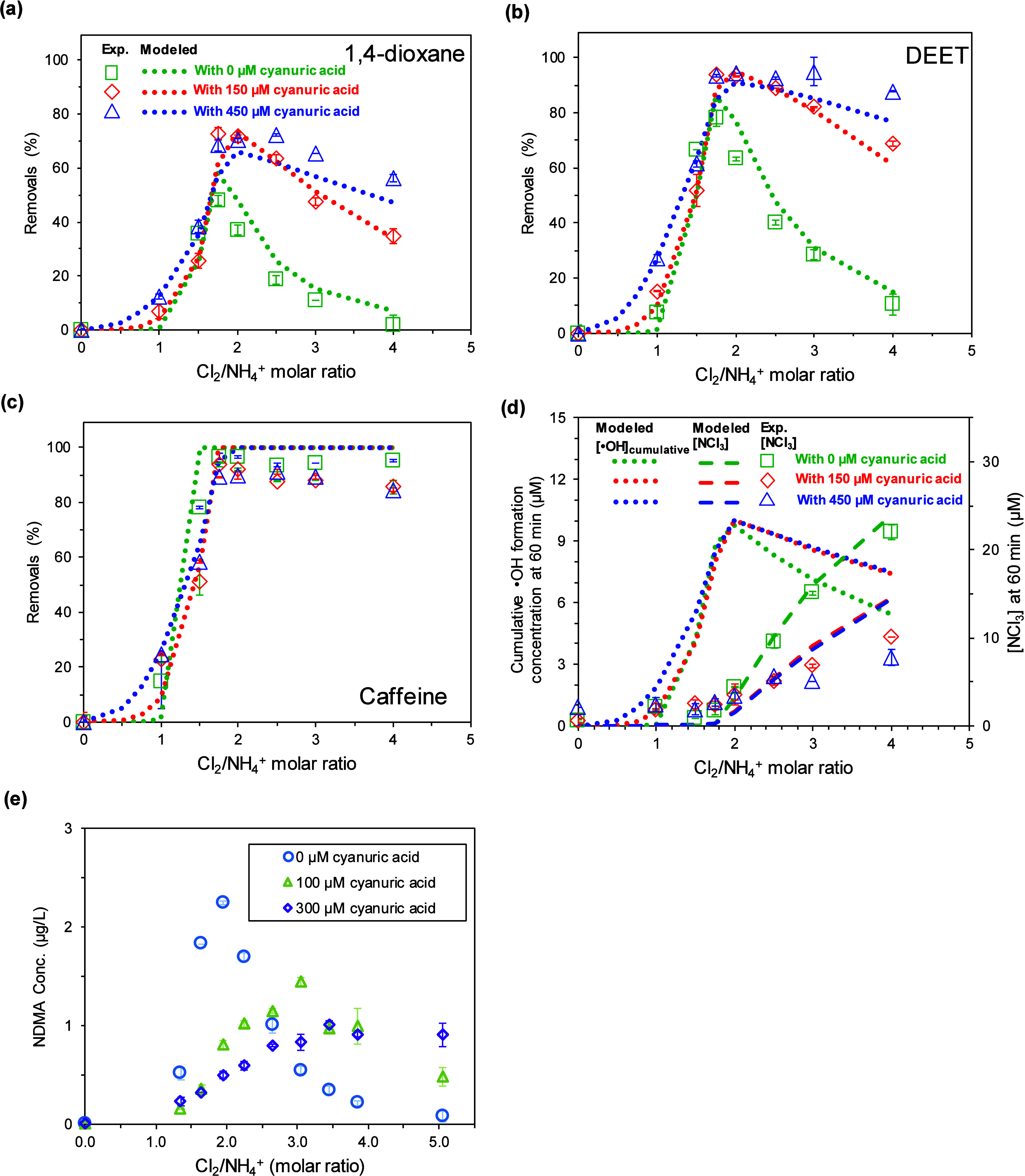
Experimental and modeled removals for (a) 1,4-dioxane, (b) DEET, and (c) caffeine during the chlorination of a mixture of 50 μM NH_4_^+^ and five micropollutants at 0.2 or 0.4 μM at pH 7 by 0–200 μM HOCl with or without cyanuric acid. (a)–(c) share the same legend. (d) Modeled cumulative formation concentration for •OH, and modeled and experimental NCl_3_ formation concentration at 60 min after the treatments (experimental details and analyses provided in Text S1-D). (e) NDMA formation concentration at 60 min after the treatments of a solution containing 7.5 μM Cl-DMA and 50 μM NH_4_^+^ by HOCl at pH 7.0 with 0–300 μM cyanuric acid. Error bar represents data range from experimental duplicates.

Kinetic modeling well captured these trends and offered mechanistic insights. The observed removal pattern is primarily governed by the interplay between •OH generation and •OH scavenging by free chlorine. At the breakpoint, cumulative •OH formation reaches its maximum due to a series of reactions through which NHCl_2_ is the hub. Following its formation, NHCl_2_ reacts with HOCl to form NCl_3_, which subsequently reacts with NHCl_2_ to yield •OH, N_2_, ClNO while regenerating NHCl_2_ through parallel pathways (Reactions U12, U12b, and U12c in Scheme 2). This cyclic process results in an observed stoichiometry where 2.6 mol of NCl_3_ are consumed per mole of NHCl_2_.^19^ The optimal Cl_2_/NH_4_^+^ ratio (i.e., at breakpoint) enhances NHCl_2_–NCl_3_ interactions while minimizing the residual levels of both NHCl_2_ and NCl_3_ after reaction (as reported in the literature^68,69^ and in Figure 3d), thus maximizing •OH production and micropollutant degradation (Figure 3a–c).

Beyond the breakpoint (Cl_2_/NH_4_^+^ > 2), the chemical dynamics shift. Excess HOCl rapidly drives the conversion of NHCl_2_ to NCl_3_ (via U11 in Scheme 2), reducing the availability of NHCl_2_ for •OH-generating reactions. As a result, NCl_3_ accumulates and •OH formation declines (Figure 3d). Additionally, free chlorine competes with micropollutants as a major •OH scavenger. Model simulations indicate that free chlorine scavenges approximately 75% of the •OH formed at Cl_2_/NH_4_^+^> 2.5, compared to 53% at the breakpoint (Figure S23).

The presence of cyanuric acid significantly altered this behavior by enhancing micropollutant removals and mitigating the postbreakpoint decline. At Cl_2_/NH_4_^+^ = 4, removals for micropollutants except caffeine increased from <15% without cyanuric acid to 56–88% with 450 μM cyanuric acid. Two key mechanisms contribute to this enhancement. First, cyanurate-bound HClCy^–^ converts NHCl_2_ to NCl_3_ (i.e., reaction R6) at a rate approximately one-third that of HOCl, thereby prolonging the lifetime of NHCl_2_ and expanding the window for NHCl_2_–NCl_3_ interactions; model predictions show 12–36% higher cumulative •OH formation within the Cl_2_/NH_4_^+^ range of 2.5–4 when cyanuric acid is present (Figure 3d). Second, cyanuric acid markedly reduced •OH scavenging by free chlorine by more than 50% over the same Cl_2_/NH_4_^+^ range (Figure S23), thus increasing the fraction of •OH available for micropollutant degradation. Cyanuric acid also suppressed NCl_3_ accumulation; modeled NCl_3_ concentrations were more than 40% lower with cyanuric acid than without (Figure 3d), consistent with the extended NHCl_2_ reaction window and increased •OH generation. Experimental measurements confirmed these predictions (Figure 3d).

Structural trade-offs among micropollutants emerged in both systems. •OH scavenging by HOCl generates •ClO, a selective oxidant that is of high reactivity with imidazole moieties.^50^ This accounts for the nearly complete removal of caffeine postbreakpoint (Figure 3c). With cyanuric acid, reduced HOCl scavenging lowered •ClO formation and thus decreasing caffeine removal, although the model predictions did not capture this behavior due to the lack of comprehensive •ClO-related reactions. When the experiments were repeated with real pool water, a similar but less pronounced removal pattern was observed, attributable to radical scavenging by additional inorganic and organic constituents (Figure S24).

Lastly, cyanuric acid significantly affected nitrosamine formation. In separate experiments using Cl-DMA as the NDMA precursor, NDMA formation in the absence of cyanuric acid followed a volcano-shaped trend with respect to the Cl_2_/N molar ratio (Figure 3e), consistent with patterns observed in micropollutant degradation. Increasing cyanuric acid concentrations to 300 μM broadened the NDMA formation profiles, with peak concentrations shifting toward higher Cl_2_/N ratios and overall maximum NDMA levels decreasing. These trends suggest that ClNO generated during breakpoint chlorination may react not only with HOCl/OCl^–^ but also with (chlorinated) cyanurates, and the observed NDMA formation behavior likely reflects the competitive consumption of ClNO among oxidants, cyanuric acid, and precursors. Indeed, competition kinetics using ClNO vapor revealed a *k*_ClNO_ for H_3_Cy/H_2_Cy^–^ of 5.8 × 10^5^ M^–1^s^–1^ at pH 7 (Text S2), approximately an order of magnitude lower than that for Cl-DMA (6.8 × 10^6^ M^–1^s^–1^). Although the *k*_ClNO_ for HOCl/OCl^–^ could not be determined due to limitations of the vapor-phase approach, model simulations suggest it lies between the values for ClNO with cyanuric acid and ClNO with Cl-DMA (additional discussion in Text S8). This inference is supported by the closer agreement between model-predicted concentration of products from the ClNO–Cl-DMA reactions and experimentally observed NDMA formation concentrations. Nonetheless, further investigation is warranted to refine the kinetic model by comprehensively characterizing the aqueous-phase reactivity and fate of ClNO.

## Environmental Implications

Cyanuric acid is widely used in outdoor swimming pools as a stabilizer to reduce chlorine photodegradation. While its role in preserving chlorine residuals is well recognized, its broader impact on chlorination chemistry and DBP formation in ammonia-containing water has remained underexplored. Our experimental results show that chlorinated cyanurates (e.g., HClCy^–^) function as effective chlorinating agents for NH_2_Cl and NHCl_2_, with reaction rates approximately half those of HOCl. These findings suggest that chlorinated cyanurates may contribute to chlorination of low-molecular-weight amines, which are important precursors for nitrogenous DBPs,^70^ although they exhibit low reactivity toward phenolic compounds.

Accurate estimation of free chlorine concentrations, which is critical for assessing disinfection efficacy, depends on reliable hydrolytic dissociation constants for chlorinated cyanurates. Using phenols as probes, we confirm that the electrochemically determined values for Cl_2_Cy^–^ (p*K*_7_ = 4.1) and HClCy^–^ (p*K*_9_ = 4.8) better predict free chlorine levels than previously reported spectrophotometric values, yielding concentrations nearly five times higher under typical pool conditions. These insights are particularly important given the limitations of standard DPD-based chlorine measurements, which cannot differentiate HOCl from chlorinated cyanurates.

Although cyanuric acid is primarily used in outdoor pools, it is occasionally applied in indoor settings in some countries (e.g., China).^1,2^ Because cyanuric acid slows the formation and accumulation of trichloramine, its use in indoor pools may help mitigate exposure to this volatile irritant, which is associated with skin, eye, and respiratory symptoms.

Swimmer-derived inputs such as ammonia, organic nitrogen, and micropollutants can lead to substantial local variation in chlorine-to-nitrogen ratios within pool water. The presence of cyanuric acid alters the fate of chlorine-derived radicals and nitrosating agents in such microenvironments, leading to complex trade-offs between disinfection efficacy, contaminant removal, and DBP formation. This is supported by our kinetic modeling (Text S9), which simulated micropollutant removal and nitrosamine formation potential under realistic pool conditions where free chlorine is steadily maintained while ammonia is continuously introduced by swimmers. The prolonged lifetime for •OH, while enhancing micropollutant degradation, may also compensate the reduced disinfection efficacy due to low HOCl equilibrium concentration. Prior research highlights that breakpoint-chlorination-induced reactive species improve disinfection of hard-to-kill organisms like Amoeba spores and their internal bacteria.^32^

Lastly, given that cyanuric acid is predominantly applied in sunlit outdoor pools, the potential influence of irradiation on the kinetics and mechanisms of chlorine–ammonia–cyanuric acid reactions, as well as their implications for DBP formation, warrants further investigation.

## Supplementary Material



## Notes

Chemical lists, additional experimental details and analytical methods, figures for micropollutant removals during the treatments of breakpoint chlorination with or without cyanuric acid, kinetic model development, and additional experimental results and discussion. (PDF)

## References

[1] Li Y., Chen L., Li H., Peng F., Zhou X., Yang Z. (2020). Occurrence, distribution, and health risk assessment of 20 personal
care products in indoor and outdoor swimming pools. Chemosphere.

[2] Li J., Chen J., Hu Z., Li X., Li M., Wang Y., Zhang Z., Liang X. (2023). Overlooked
inorganic
DBPs in trichloroisocyanuric acid (TCCA) disinfected indoor swimming
pool: Evidences from concentration, cytotoxicity, and human health
risk. Chemosphere.

[3] Wahman D. G. (2018). Chlorinated
cyanurates: Review of water chemistry and associated drinking water
implications. J. Am. Water Works Ass..

[4] Cantú R., Evans O., Kawahara F. K., Wymer L. J., Dufour A. P. (2001). HPLC Determination
of Cyanuric Acid in Swimming Pool Waters Using Phenyl and Confirmatory
Porous Graphitic Carbon Columns. Anal. Chem..

[5] Anderson J.
R. (1965). A study
of the influence of cyanuric acid on the bactericidal effectiveness
of chlorine. American Journal of Public Health.

[6] Manasfi T., Coulomb B., Boudenne J.-L. (2017). Occurrence, origin, and toxicity
of disinfection byproducts in chlorinated swimming pools: An overview. Int. J. Hyg Envir Heal.

[7] Teo T. L., Coleman H. M., Khan S. J. (2015). Chemical
contaminants in swimming
pools: Occurrence, implications and control. Environ. Int..

[8] Walse S. S., Mitch W. A. (2008). Nitrosamine carcinogens also swim
in chlorinated pools. Environ. Sci. Technol..

[9] Kim H., Han K. (2011). Swimmers contribute
to additional formation of N-nitrosamines in
chlorinated pool water. Toxicology Environmental
Health Sciences.

[10] Erdinger L., Kirsch F., Sonntag H. (1997). Potassium as an indicator
of anthropogenic
contamination of swimming pool water. Int. J.
Hygiene Environ. Med..

[11] Judd S. J., Black S. H. (2000). Disinfection by-product formation in swimming pool
waters: a simple mass balance. Water Res..

[12] Krasner S. W., McGuire M. J., Jacangelo J. G., Patania N. L., Reagan K. M., Aieta E. M. (1989). The occurrence of disinfection by-products in US drinking
water. J. Am. Water Works Assoc.

[13] Kanan, A. A. Occurrence and formation of disinfection by-products in indoor swimming pools water. Doctoral Thesis. Clemson University, 2010.

[14] Weng S., Sun P., Ben W., Huang C.-H., Lee L. T., Blatchley E. R. (2014). The Presence of Pharmaceuticals and Personal Care Products
in Swimming Pools. Environ. Sci. Technol. Lett..

[15] Leavens T.
L., Blount B. C., DeMarini D. M., Madden M. C., Valentine J. L., Case M. W., Silva L. K., Warren S. H., Hanley N. M., Pegram R. A. (2007). Disposition of Bromodichloromethane in Humans Following
Oral and Dermal Exposure. Toxicol. Sci..

[16] Jafvert C. T., Valentine R. L. (1992). Reaction scheme for the chlorination of ammoniacal
water. Environ. Sci. Technol..

[17] Vikesland P. J., Ozekin K., Valentine R. L. (2001). Monochloramine
decay in model and
distribution system waters. Water Res..

[18] Wahman D. G. (2018). Web-Based
Applications to Simulate Drinking Water Inorganic Chloramine Chemistry. J.-Am. Water Works Assoc..

[19] Chuang Y. H., Chen T. Y., Chou C. S., Chu L. K., Hou C. Y., Szczuka A. (2023). Critical Role of Trichloramine Interaction with Dichloramine
for N-Nitrosamine Formation during Breakpoint Chlorination. Environ. Sci. Technol..

[20] Chuang Y.-H., Chou C.-S., Chu Y.-L. (2024). Unveiling
the Critical Pathways of
Hydroxyl Radical Formation in Breakpoint Chlorination: The Role of
Trichloramine and Dichloramine Interactions. Environ. Sci. Technol..

[21] Huang H., Zheng H., Jiao J., Lei Y., Zhou Y., Qiu J., Yang X. (2022). Trichloramine and Hydroxyl
Radical Contributions to
Dichloroacetonitrile Formation Following Breakpoint Chlorination. Environ. Sci. Technol..

[22] Wagner E. D., Plewa M. J. (2017). CHO cell cytotoxicity
and genotoxicity analyses of
disinfection by-products: An updated review. Journal of Environmental Sciences.

[23] APHA , Method 4500-Cl, Standard Methods for The Examination of Water and Wastewater. 20th ed.; American Water Works Association & Water Environment Federation: Washington, D.C., USA, 1998.

[24] Wahman D. G., Alexander M. T. (2019). A Drinking
Water Relevant Water Chemistry Model for
the Free Chlorine and Cyanuric Acid System from 5 to 35 °C. Environ. Eng. Sci..

[25] O’Brien, J. E. ; Morris, J. C. ; Butler, J. N. , Equilibria in Aqueous Solutions of Chlorinated Isocyanurate. In Equilibria in Aqueous Solutions of Chlorinated Isocyanurate. In Chemistry of Water Supply, Treatment, and Distribution.; Ann Arbor Science Publishers Inc.: Ann Arbor, Mich., 1974.

[26] Pinsky M. L., Hu H.-C. (1981). Evaluation of the
chloroisocyanurate hydrolysis constants. Environ.
Sci. Technol..

[27] Jensen J. N., Johnson J. D. (1989). Quantitation of interferences under equilibrium conditions
with application to free chlorine analysis in the presence of organic
chloramines. Anal. Chem..

[28] Wojtowicz J. A. (1996). Reevaluation
of Chloroisocyanurate Hydrolysis Constants. J. Swimming Pool Spa Ind..

[29] Wahman, D. Free chlorine and cyanuric acid simulator application description–Version 0.50; EPA/600/S–17/165, United States Environmental Protection Agency, Washington, DC, 2017.

[30] Tachikawa M., Sayama C., Saita K., Tezuka M., Sawamura R. (2002). Effects of
isocyanuric acid on the monochlorodimedone chlorinating rates with
free chlorine and ammonia chloramine in water. Water Res..

[31] Wang W. L., Wu Q. Y., Du Y., Huang N., Hu H. Y. (2018). Elimination
of chlorine-refractory carbamazepine by breakpoint chlorination: Reactive
species and oxidation byproducts. Water Res..

[32] Wang L., Mai Y., Li S., Shu L., Fang J. (2023). Breakpoint Chlorination
Enhances the Disinfection of Amoeba Spores and Their Intraspore Bacteria. Environ. Sci. Technol. Lett..

[33] Patton S. D., Dodd M. C., Liu H. (2022). Degradation
of 1,4-dioxane by reactive
species generated during breakpoint chlorination: Proposed mechanisms
and implications for water treatment and reuse. Journal of Hazardous Materials Letters.

[34] Schreiber I. M., Mitch W. A. (2007). Enhanced nitrogenous
disinfection byproduct formation
near the breakpoint: Implications for nitrification control. Environ. Sci. Technol..

[35] Chuang Y. H., Shi H. J. (2022). UV/chlorinated cyanurates
as an emerging advanced oxidation
process for drinking water and potable reuse treatments. Water Res..

[36] Bulman D. M., Mezyk S. P., Remucal C. K. (2019). The impact of pH
and irradiation
wavelength on the production of reactive oxidants during chlorine
photolysis. Environ. Sci. Technol..

[37] Schreiber I. M., Mitch W. A. (2005). Influence of the order of reagent addition on NDMA
formation during chloramination. Environ. Sci.
Technol..

[38] Schurter L. M., Bachelor P. P., Margerum D. W. (1995). Nonmetal Redox Kinetics: Mono-, Di-,
and Trichloramine Reactions with Cyanide Ion. Environ. Sci. Technol..

[39] Burttschell R. H., Rosen A. A., Middleton F. M., Ettinger M. B. (1959). Chlorine derivatives
of phenol causing taste and odor. J. Am. Water
Works Ass..

[40] Lee G. F., Morris J. C. (1962). Kinetics of chlorination
of phenol - chlorophenolic
tastes and odors. Int. J. Air Water Poll.

[41] Lau S. S., Abraham S. M., Roberts A. L. (2016). Chlorination
Revisited: Does Cl–
Serve as a Catalyst in the Chlorination of Phenols?. Environ. Sci. Technol..

[42] Gallard H., Von Gunten U. (2002). Chlorination
of phenols: Kinetics and formation of
chloroform. Environ. Sci. Technol..

[43] Patton S., Li W., Couch K. D., Mezyk S. P., Ishida K. P., Liu H. (2017). Impact of
the ultraviolet photolysis of monochloramine on 1,4-dioxane removal:
New insights into potable water reuse. Environ.
Sci. Technol. Lett..

[44] Buxton G. V., Greenstock C. L., Helman W. P., Ross A. B. (1988). Critical
Review
of rate constants for reactions of hydrated electrons, hydrogen atoms
and hydroxyl radicals (•OH/•O^–^) in
aqueous solution. J. Phys. Chem. Ref. Data.

[45] Thomas J. (1965). Rates of reaction
of the hydroxyl radical. T Faraday Soc..

[46] Lei Y., Yu Y., Lei X., Liang X., Cheng S., Ouyang G., Yang X. (2023). Assessing
the Use of Probes and Quenchers for Understanding the Reactive
Species in Advanced Oxidation Processes. Environ.
Sci. Technol..

[47] Lu S., Shang C., Sun B., Xiang Y. (2023). Dominant Dissolved
Oxygen-Independent Pathway to Form Hydroxyl Radicals and the Generation
of Reactive Chlorine and Nitrogen Species in Breakpoint Chlorination. Environ. Sci. Technol..

[48] Lei Y., Cheng S. S., Luo N., Yang X., An T. C. (2019). Rate Constants
and Mechanisms of the Reactions of •Cl^–^ and
•Cl_2_
^–^ with Trace Organic Contaminants. Environ. Sci. Technol..

[49] The NIST database . NDRL/NIST Solution Kinetics Database (Web pages; https://kinetics.nist.gov/solution/). http://kinetics.nist.gov/solution/ (2025.01.01 accessed).

[50] Sun P. Z., Lee W. N., Zhang R. C., Huang C. H. (2016). Degradation of DEET
and caffeine under UV/chlorine and simulated sunlight/chlorine conditions. Environ. Sci. Technol..

[51] Guo K., Wu Z., Shang C., Yao B., Hou S., Yang X., Song W., Fang J. (2017). Radical chemistry
and structural
relationships of PPCP degradation by UV/chlorine treatment in simulated
drinking water. Environ. Sci. Technol..

[52] Benitez F. J., Acero J. L., Real F. J., Roldan G., Rodriguez E. (2013). Modeling the
photodegradation of emerging contaminants in waters by UV radiation
and UV/H2O2 system. Journal of Environmental
Science and Health, Part A.

[53] Song W., Cooper W. J., Peake B. M., Mezyk S. P., Nickelsen M. G., O’Shea K. E. (2009). Free-radical-induced
oxidative and reductive degradation
of N, N′-diethyl-m-toluamide (DEET): Kinetic studies and degradation
pathway. Water Res..

[54] Soltermann F., Lee M., Canonica S., von Gunten U. (2013). Enhanced N-nitrosamine formation
in pool water by UV irradiation of chlorinated secondary amines in
the presence of monochloramine. Water Res..

[55] Tricker A. R., Pfundstein B., Kälble T., Preussmann R. (1992). Secondary
amine precursors to nitrosamines in human saliva, gastric juice, blood,
urine and faeces. Carcinogenesis.

[56] Stanbury D. M. (2024). Kinetics
and Equilibria Interconverting Aqueous Inorganic Chloramines: Errors
and Corrections. ACS ES&T Water.

[57] Ianni, J. C. Kintecus, Windows Version 6.8. www.kintecus.com, 2019.

[58] Gallard H., von Gunten U. (2002). Chlorination
of natural organic matter: kinetics of
chlorination and of THM formation. Water Res..

[59] Gardiner J. (1973). Chloroisocyanurates
in the treatment of swimming pool water. Water
Res..

[60] Brady A. P., Sancier K. M., Sirine G. (1963). Equilibria in Solutions
of Cyanuric
Acid and its Chlorinated Derivatives. J. Am.
Chem. Soc..

[61] Fukuyama M., Flanagan P. W. K., Williams F. T., Frainier L., Miller S. A., Shechter H. (1970). Thermodynamic and kinetic acidity
properties of nitroalkanes. Correlation of the effects of structure
on the ionization constants and the rate constants of neutralization
of substituted 1-phenyl-1-nitroethanes. J. Am.
Chem. Soc..

[62] Matte D., Solastiouk B., Merlin A., Deglise X. (1990). Étude cinétique
de la N-chloration de l’acide cyanurique en phase aqueuse. Can. J. Chem..

[63] Jensen J. N., Johnson J. D. (1990). Interferences by
monochloramine and organic chloramines
in free available chlorine methods. 2. N,N-Diethyl-p-phenylenediamine. Environ. Sci. Technol..

[64] Song D., Liu H., Qiang Z., Qu J. (2014). Determination of rapid chlorination
rate constants by a stopped-flow spectrophotometric competition kinetics
method. Water Res..

[65] Valentine R. L., Jafvert C. T. (1988). General acid catalysis
of monochloramine disproportionation. Environ.
Sci. Technol..

[66] Zhang T., von Gunten U. (2023). Chlorination of amides: Kinetics and mechanisms of
formation of N-chloramides and their reactions with phenolic compounds. Water Res..

[67] Ye B., Wu Q.-Y., Wang W.-L., Hu H.-Y. (2023). PPCP degradation
by ammonia/chlorine: Efficiency, radical species, and byproducts formation. Water Res..

[68] Shang C., Blatchley E. R. (1999). Differentiation and Quantification
of Free Chlorine
and Inorganic Chloramines in Aqueous Solution by MIMS. Environ. Sci. Technol..

[69] Shang C., Gong W.-L., Blatchley E. R. (2000). Breakpoint
Chemistry and Volatile
Byproduct Formation Resulting from Chlorination of Model Organic-N
Compounds. Environ. Sci. Technol..

[70] Bond T., Templeton M. R., Graham N. (2012). Precursors of nitrogenous disinfection
by-products in drinking water––A critical review and
analysis. J. Hazard. Mater..

