# Supplementary material for: Breakpoint Chlorination Chemistry in a Chlorine-Cyanurate System and Trade-Offs between Nitrosamine Formation and Micropollutant Removals
Source: Environ Sci Technol. 2025 Nov 18;59(47):25390–401. doi: 10.1021/acs.est.5c09618 (PMC12676743; doi:10.1021/acs.est.5c09618)
Supplement: Supplementary file 1 [file es5c09618_si_001.pdf]

Supporting Information

for

Breakpoint chlorination chemistry in chlorine-cyanurate system and trade-offs between nitrosamine formation and micropollutant removals

Yi-Hsueh Chuang[1,*], Chia-Shun Chou[1], Yi-Lin Chu[1], and Yen-Pao Chiang[1]

[1]Institute of Environmental Engineering, National Yang Ming Chiao Tung University

No. 1001, University Rd., Hsinchu city, Taiwan 30010

*corresponding author: yhchuang@nycu.edu.tw

This supporting information contains:

9 Texts

10 Tables

26 Figures

1 Scheme

Table S1. Chemicals

Chemical	Concentration or purity	Supplier
Dimethylamine	40 wt.%	Acros Organics
EPA 521 Nitrosamine Mix	2000 µg/mL each component in dichloromethane	Sigma-Aldrich
EPA521 internal standard	1000 µg/L in Methylene Chloride	Accustandard
Dichloromethane	99.9%	Honeywell
Sodium hypochlorite	min.6%	Duksan
Ammonium chloride	ACS grade	Macron
Sodium hydroxide	≥98%	Honeywell
Potassium phosphate monobasic	>99%	Acros Organics
Sodium sulfite	≥98%	Acros Organics
Sodium chloride	99.5%	Honeywell
Orthophosphoric acid	85%	Fisher Scientific
Sodium nitrite	98%	Alfa Aesar
Sodium nitrate	≥98%	Alfa Aesar
Ascorbic acid	Reagent grade	Sigma-Aldrich
Potassium iodide	>99.5%	Honeywell Fluka
DPD Oxalate N,N-Diethyl-p-Phenylenediamine	Analytical Grade	Hach
Hydrogen peroxide	34.5%-36.5%	Honeywell
1,4-dioxane	99.8%	Thermo Scientific
DEET	98%	Acros Organics
Caffeine	99%	Alfa Aesar
Nitrobenzene	99%	Thermo Scientific
Benzoic acid	99%	Thermo Scientific
1,4-dioxane-d8	> 99%	Acros Organics
Amberlite IRC120 H resin	NA	Sigma-Aldrich
Cyanuric acid	>98.0%	Tokyo Chemical Industry

Table S2. Experiments for investigating the interaction between cyanuric acid or chlorinated cyanurates with NH_2Cl or $NHCl_2$.

Exp. No.	[Cyanuric acid], μM	$[HOCl]_{initial}$, μM	$[NH_2Cl]_{initial}$, μM	$[NHCl_2]_{initial}$, μM	pH	$[HClCy^-]_{initial}$ (calculated)[a] μM
1	400	-	50	-	7	0
2	400	-	50	-	6	0
3	400	-	50	-	6.5	0
4	400	-	50	-	7.5	0
5	400	20	20	-	7	18.6
6	400	20	30	-	7	18.6
7	400	20	50	-	7	18.6
8	400	20	75	-	7	18.6
9	400	20	100	-	7	18.6
10	400	20	125	-	7	18.6
11	400	-	-	20	7	0
12	400	20	-	20	7	18.6
13	400	20	-	30	7	18.6
14	400	20	-	40	7	18.6
15	400	20	-	55	7	18.6
16	400	20	-	70	7	18.6
17	400	20	-	80	7	18.6

[a] the initial concentration of $HClCy^-$ in each experiments were calculated using the K_7 and K_9 reevaluated in this study, along with other equilibrium constants.

Text S1. Stock solution standardization, experimental details, and the UV-spectrum-based approach for determination of the concentrations of NH_2Cl and $NHCl_2$ in the cyanuric acid experiments.

A. Stock solution standardization.

In this study, the concentration of NH_2Cl and $NHCl_2$ in the prepared stock solutions were standardized spectrometrically, using A Cary 60 UV-visible spectrophotometer. The concentrations of NH_2Cl and $NHCl_2$ were determined using the total absorbance values in conjunction with their respective molar extinction coefficients at the selected wavelengths ($\varepsilon_{NH_2Cl,245\ nm}$ = 445 $M^{-1}cm^{-1}$, $\varepsilon_{NHCl_2,245\ nm}$ = 208 $M^{-1}cm^{-1}$, $\varepsilon_{NH_2Cl,295\ nm}$ = $M^{-1}cm^{-1}$, and $\varepsilon_{NHCl_2,295\ nm}$ = 267 $M^{-1}cm^{-1}$).[1, 2]

B. Experiments

Experiments were carried out in a 10-cm path length quartz cuvette mounted in a spectrophotometer for *in situ* measurements of UV-vis spectra at wavelengths between 230–400 nm. A 10-mM or 20-mM phosphate buffer was used to maintain the pH throughout the experiments, and UV-vis spectra were taken periodically during the reaction (scan time was ~1 sec). To initiate the experiments, cyanuric acid was added at desired concentration, followed by the application of HOCl (for experiments investigating chlorinated cyanurates). For experiments involving NH_2Cl or $NHCl_2$, the solution was gently mixed by pipetting with a 1-mL pipette for ~10 s. The cuvette was capped with a TPFE lid to minimize headspace. Reaction time was tracked from the moment of NH_2Cl or $NHCl_2$ injection. No mechanical stirrer was placed in the cuvette.

The absence of a mixer does not affect the results because the system is homogeneous and the reactions are homogeneous. To confirm this, we compared NH_2Cl decomposition and $NHCl_2$ formation in two experiments using 400 µM cyanuric acid in 10 mM phosphate buffer (pH 7) treated with 50 µM NH_2Cl. In one case, the solution was mixed only at the beginning; in the other, it was mixed intermittently between spectral measurements. Both experiments produced comparable kinetic trends (Figure S2; see Text S1-**C** for analytical method), demonstrating that mixing did not influence the observed reaction behavior.

C. The UV-spectrum-based approach for determination of the concentrations of NH_2Cl and $NHCl_2$ in the cyanuric acid experiments.

A UV-spectrum/simultaneous equation approach was employed to accurately quantify the concentrations of NH_2Cl and $NHCl_2$ during the reaction of cyanuric acid with NH_2Cl. This method was developed in our previous studies for the quantification of chlorine and chloramines during the reaction of breakpoint reactions or during the reactions between NCl_3 and $NHCl_2$.[3] Briefly, the reaction of cyanuric acid with NH_2Cl was conducted in a 10 cm pathlength quartz cuvette mounted in an Agilent Cary 60 spectrophotometer for *in situ* measurements of UV-vis spectra at wavelengths between 230–400 nm. Cyanuric acid concentration (400 µM) was excess to NH_2Cl (up to 125 µM) or $NHCl_2$ (up to 80 µM) in these experiments.

Then, UV spectra were taken periodically during the reaction. For each spectrum, the concentrations of NH_2Cl and $NHCl_2$ were calculated based on the absorbance values at 280 and 310 nm, at which the 400 µM cyanuric acid absorbs negligible light, using their corresponding molar absorption coefficients, as presented in Eqs. S1–S2.[3] Note that the absorbance attributed to the cyanuric acid (i.e., $A_{280\ nm,\ cyanuric\ acid}$ and $A_{310\ nm,\ cyanuric\ acid}$ in Eqs. S1–S2) can be neglected because the 400 µM cyanuric acid does not absorb light at wavelength >245 nm (Figure S1a).

$$A_{280\ nm} = \varepsilon_{NH_2Cl,280nm} \times [NH_2Cl] + \varepsilon_{NHCl_2,280nm} \times [NHCl_2] + A_{280\ nm,cyanuric\ acid} \qquad \text{(Eq. S1)}$$

$$A_{310\ nm} = \varepsilon_{NH_2Cl,310nm} \times [NH_2Cl] + \varepsilon_{NHCl_2,310nm} \times [NHCl_2] + A_{310\ nm,cyanuric\ acid} \qquad \text{(Eq. S2)}$$

To evaluate whether unknown products interfere with the quantification of oxidant species, UV spectra for known oxidant species were reconstructed over a wavelength range of 230–400 nm. This was achieved using calculated oxidant concentrations in conjunction with wavelength-dependent molar absorption coefficients (Eq. S3). The reconstructed spectra were then compared to those measured experimentally. Perfect matches were anticipated only if NH_2Cl and $NHCl_2$ were the predominant absorbers within this wavelength range.

$$A_{\lambda} = \varepsilon_{\lambda,\ NH2Cl} \times [NH_2Cl] + \varepsilon_{\lambda,\ NHCl2} \times [NHCl_2],\ (\lambda = 230\text{–}400\ nm) + A_{\lambda,\ cyanuric\ acid} \qquad \text{(Eq. S3)}$$

Figure S1b shows the comparison between the measured spectra and the reconstructed spectra during the reaction of 400 µM cyanuric acid with 50 µM NH_2Cl (at pH 7) at 20 secs and at 5 min. The perfect match support that NH_2Cl and $NHCl_2$ are the main light absorbers in the reactions.

The reconstructed spectra matched with the measured spectra at wavelength >275 nm for the reaction of 400 µM cyanuric acid + 20 µM HOCl with 50 µM NH_2Cl, but were a bit deviated from each other at <275 nm. This is because the chlorinated cyanurates, which absorb light at 230–275 nm (Figure S1a), were consumed during the reactions; the light absorption of chlorinated cyanurates were not taken into account in Eq. S3. Nevertheless, this does not interfere the quantification of NH_2Cl and $NHCl_2$ given that they were calculated using the absorbances at 280 nm and 310 nm (i.e., Eqs. S1–S2). The perfect match of the measured spectra with the reconstructed spectra at >280 nm support that NH_2Cl and $NHCl_2$ were the main light absorber.

A set of standard samples was prepared to further validate this method by mixing NH_2Cl and $NHCl_2$ (which stably coexist) at concentrations of 1–6 µM each in a 10 mM phosphate buffer containing 400 µM cyanuric acid at pH 7. UV spectra were recorded for each sample immediately after preparation (within 20 s). The UV spectrum/simultaneous equation approach was then applied to determine NH_2Cl and $NHCl_2$ concentrations. The measured values closely agreed with the expected concentrations ($R^2 > 0.98$). To determine detection limits following the USEPA standard method,[4] seven replicates containing 5 µM each of NH_2Cl and $NHCl_2$ with 400 µM cyanuric acid were analyzed, and the minimum detection limits for both compounds were ~0.8 µM.

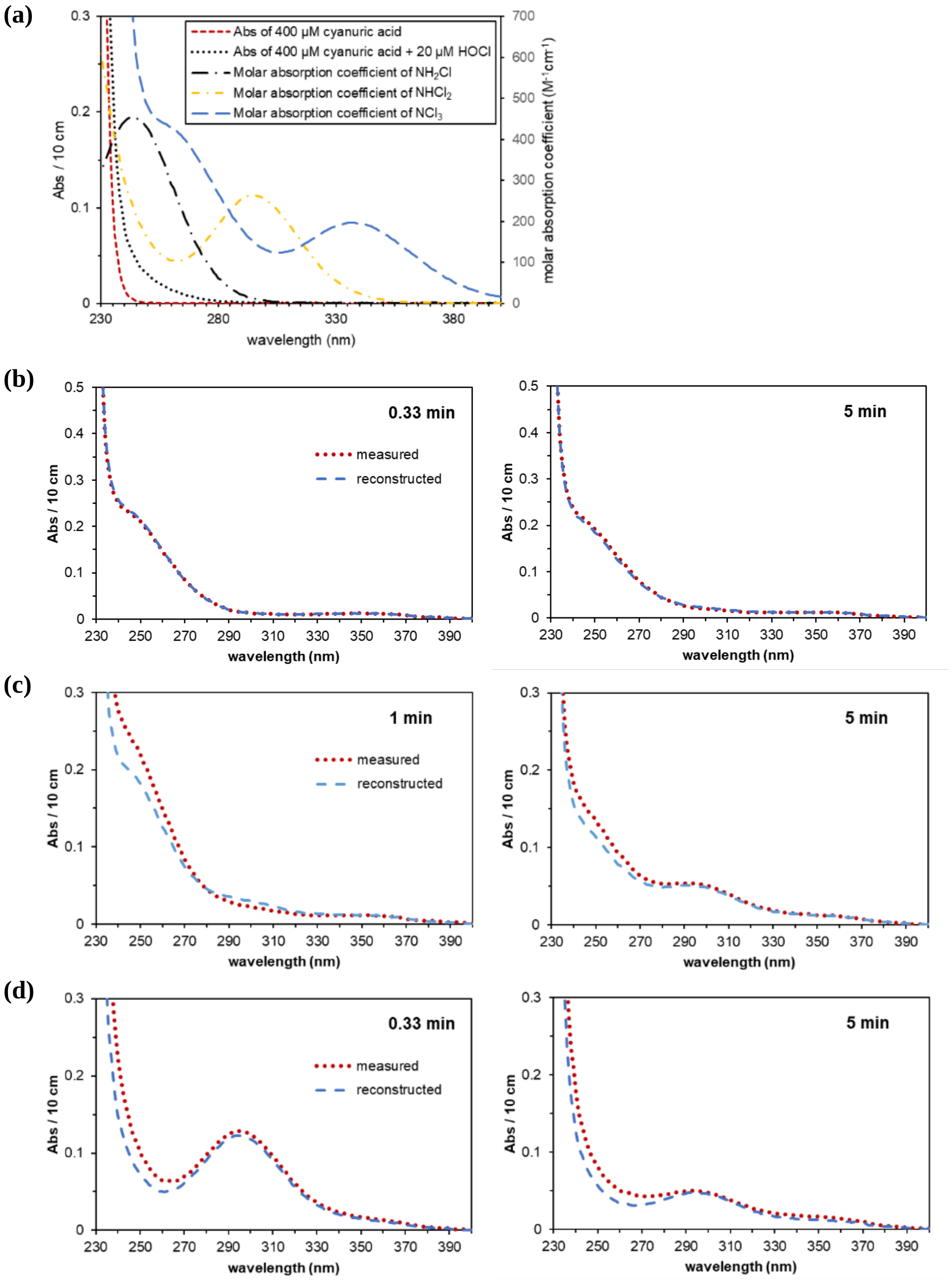

Figure S1. (a). Light absorption at 230–400 nm for 400 µM cyanuric acid, 400 µM cyanuric acid + 20 µM HOCl, and the molar absorption coefficient of inorganic chloramines. (b) Comparison between the measured spectra and the reconstructed spectra during the reaction of 400 µM cyanuric acid + 50 µM NH_2Cl, (c) 400 µM cyanuric acid + 20 µM HOCl + 50 µM NH_2Cl at different time, and (d) 400 µM cyanuric acid + 20 µM HOCl + 40 µM $NHCl_2$ at different time. These experiments were conducted at pH 7 (10 mM phosphates).

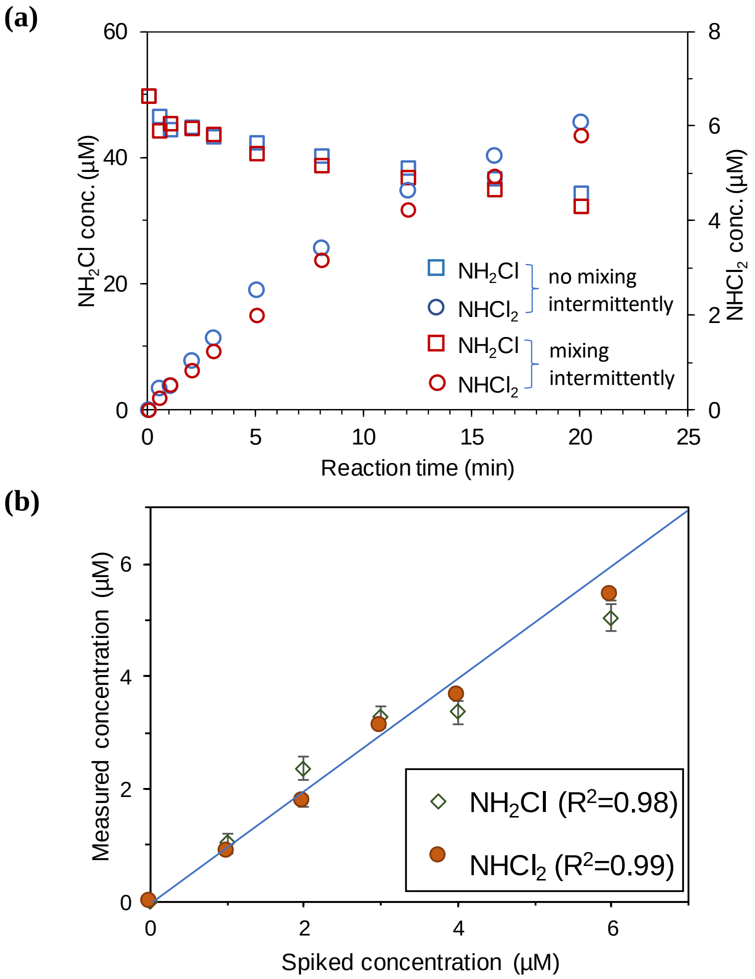

Figure S2. (a) NH_2Cl and $NHCl_2$ concentration during the reaction of 400 µM cyanuric acid with 50 µM NH_2Cl (in 10 mM phosphate buffer at pH 7), with or without intermittent mixing. (b) NH_2Cl and $NHCl_2$ concentrations determined in the standard samples containing 400 µM cyanuric acid (in 10 mM phosphate buffer at pH 7) with NH_2Cl and $NHCl_2$ at 0–6 µM each.

D. UV-spectrum-based determination of NCl_3 concentration in breakpoint chlorination reactions with or without cyanuric acid.

In one experiment, the NCl_3 concentration was determined 60 min after chlorinating ammonia-containing water (50 µM NH_4^+) with varying Cl_2:NH_4^+ molar ratios (0–4) at pH 7, in the presence of 0–450 µM cyanuric acid. The reaction was carried out directly in a UV–vis spectrometer using a 10-cm path length quartz cuvette capped with a PTFE lid to minimize headspace. UV spectra were recorded before and after 60 min of reaction. The NCl_3 concentration was quantified from the change in absorbance at 360 nm ($\varepsilon_{360\ nm}$ = 126 $M^{-1}cm^{-1}$).[2]

Table S3. Summary of the rate constants of micropollutants with radicals.

Micropollutant	$k_{\bullet OH}$ ($\times 10^9$ $M^{-1}s^{-1}$)	$k_{\bullet Cl}$ ($\times 10^9$ $M^{-1}s^{-1}$)	$k_{\bullet ClO}$ ($\times 10^8$ $M^{-1}s^{-1}$)	$k_{\bullet Cl2-}$ ($\times 10^6$ $M^{-1}s^{-1}$)	RNS[a] ($M^{-1}s^{-1}$)	References
1,4-Dioxane	2.5–3.2	0.0044	NA	<0.05–3.3	NA	[5-7]
Nitrobenzene	3.2–4.7	0.52	0.002	<0.5	Negligible	[6, 8, 9]
Benzoate	5.5–6.2	14	<0.03	<2	Negligible	[8-11]
Caffeine	6.4	14.6–38.7	1.03	9.28×10^8	NA	[10, 12, 13]
DEET	5.0–7.5	3.8	slow	NA	$\leq 1\times 10^9$	[9, 12, 14, 15]

[a] RNS may include •NO, $\bullet NO_2$, $\bullet NH_2$. NA = not available.

Text S2. Additional experimental details

A. Determination of reaction rate constant between ClNO and Cl-DMA.

To determine the reaction rate constant of ClNO with Cl-DMA, we monitored the degradation of Cl-DMA in a solution containing Cl-DMA, NO_2^-, and Cl^- at pH 3 buffered with 10 mM phosphates. ClNO equilibrates with NO_2^-, H^+, and Cl^-.[16, 17]

$$HNO_2 + H^+ + Cl^- \leftrightarrow ClNO + H_2O \qquad K = 1.1\times10^{-3}\ M^{-2}$$

$$H^+ + NO_2^- \leftrightarrow HNO_2 \qquad K = 1/K_{a,\ HNO2} = 1.4\times10^3$$

To initiate an experiment, a 0.5 M NaCl solution buffered at pH 3 (10 mM phosphate) was spiked with 200 µM NO_2^-, followed by the addition of 12 µM Cl-DMA. Reactions were terminated by raising pH to 7 with a pre-determined dose of NaOH. No quencher was added because quenchers (e.g., sodium thiosulfate or ascorbic acid) decompose Cl-DMA. Raising the pH effectively terminated nitrosation reactions and Cl-DMA in the pH adjusted solution was stable for more than 6 h. The samples were immediately analyzed by an Agilent HPLC (1260 II) coupled with a UV detector, and the time for analyses were within 6 h.

The equilibrium constants suggest an equilibrium concentration of 1.55×10^{-10} M for ClNO in the mixture of 200 µM NO_2^- and 0.5 M Cl^- at pH 3. While the equilibrium concentration of ClNO was orders of magnitude lower than the Cl-DMA concentration, Le Chatelier's principle indicates that the system restores equilibrium by producing more ClNO as ClNO is consumed, such that ClNO concentration would be steady. Indeed, experimental results demonstrated that Cl-DMA degradation followed pseudo-first order kinetics (Figure S3a). The rate constant for the reaction of ClNO with Cl-DMA was determined to be 6.8×10^6 $M^{-1}s^{-1}$. While previous research indicated nitrosation reaction of nitrite with amines occurs at acidic pH,[16] our recent study indicated negligible reaction between Cl-DMA and NO_2^- at pH 3 without Cl^-.[3]

B. Competition kinetics approach for determining rate constant of ClNO with H_3Cy/H_2Cy^-

To determine the rate constant for the reaction between ClNO and H_3Cy/H_2Cy^-, we employed a competitive kinetics approach by monitoring the decomposition of Cl-DMA and cyanuric acid in mixed solutions. Experiments were conducted at pH 7 in 20 mM phosphate buffer containing 1 mM Cl-DMA and 1 mM cyanuric acid. A series of samples were prepared in 22-mL glass vials (10 mL per vial), each spiked with varying amounts (0–150 µL) of ClNO vapor. ClNO vapor was generated by mixing 0.5 mL of 8 M $NaNO_2$ with 0.5 mL of 6 M HCl in a 40-mL Teflon-lined screw-top vial, following established procedures.[18] The vapor was immediately transferred to the sample vials using a microsyringe and stirred to promote gas–liquid interaction, as previously described.[19] The pH of each solution was maintained at 7.0±0.1 throughout the

reactions using the phosphate buffer. Because the first pK_a of cyanuric acid is 6.88, both H_3Cy and H_2Cy^- are present in solution at pH 7. The observed rate constant from this study thus reflects the average reactivity of ClNO with the H_3Cy/H_2Cy^- mixture.

Cl-DMA and cyanuric acid concentrations were quantified using an Agilent HPLC-UV, and the analytical method for cyanuric acid is available in our previous study.[20] The ClNO added was deliberately kept limiting relative to Cl-DMA and cyanuric acid, such that their decays were each less than 40%. This ensured that the extent of reaction could be attributed to relative reactivity. Under these conditions, the following relationship applies:

$$\frac{k_{ClNO,H_3Cy/H_2Cy^-}}{k_{ClNO,Cl-DMA}} = \frac{H_3Cy/H_2Cy^- \text{ consumed by ClNO}}{Cl - DMA \text{ consumed by ClNO}} = \frac{[H_3Cy/H_2Cy^-]_0 - [H_3Cy/H_2Cy^-]}{[Cl - DMA]_0 - [Cl - DMA]}$$

Plotting the consumption of H_3Cy/H_2Cy^- versus that of Cl-DMA across all samples yielded a linear relationship with a slope of 0.105 (Figure 3b), indicating that cyanuric acid reacts with ClNO at one-tenth the rate of Cl-DMA. Given that the second-order rate constant for ClNO with Cl-DMA is 6.8×10^6 $M^{-1}s^{-1}$, the rate constant for ClNO with H_3Cy/H_2Cy^- is determined to be 6.9×10^5 $M^{-1}s^{-1}$.

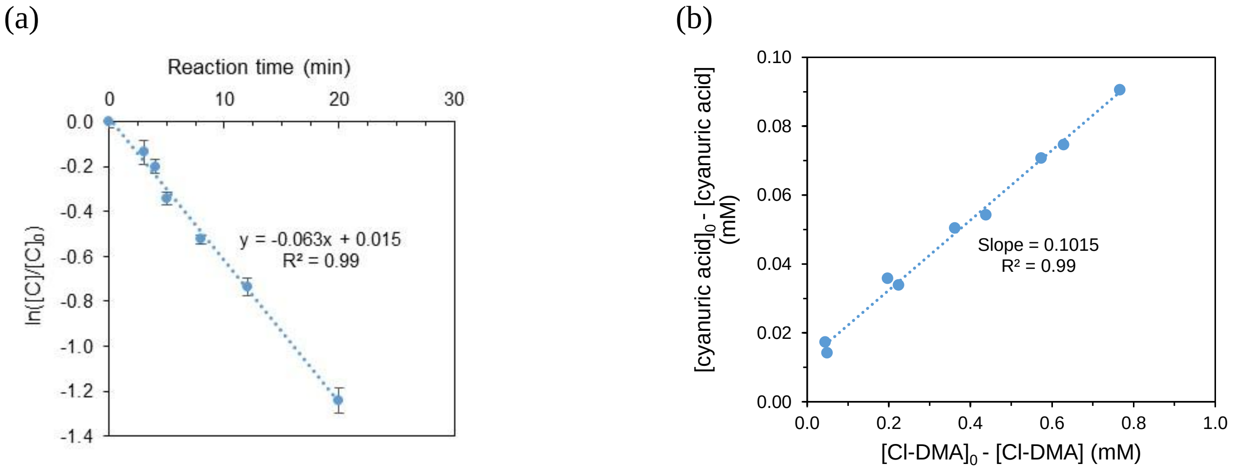

Figure S3. (a) Concentration changes of Cl-DMA during treatment of 12 μM Cl-DMA in 0.5 M NaCl solution buffered at pH 3 with 10 mM phosphate and spiked with 200 μM NO_2^-. Error bar represents data range from experimental duplicates. (b) Relationship between the changes in cyanuric acid and Cl-DMA concentrations in 10 mL solutions containing 1 mM Cl-DMA and 1 mM cyanuric acid, buffered at pH 7 with 20 mM phosphate, and exposed to varying amounts of ClNO vapor.

Table S4. Principal reactions in the breakpoint reactions kinetic model. Note that the rate constants for reaction Nos. S2 and S4 were revised according to Stanbury's work[21] to fix the bad reversible loop issue (see footnote).

**No.**	***k***	**Unit for *k***	**Reaction**	**Note**	**Ref.**
Breakpoint chlorination reactions					
S1	4.17×10^{6}	$M^{-1}s^{-1}$	$HOCl + NH_3 \rightarrow NH_2Cl + H_2O$		22
S2	1.10×10^{-4}	s^{-1}	$NH_2Cl + H_2O \rightarrow HOCl + NH_3$		21, 22
S3	2.78×10^{2}	$M^{-1}s^{-1}$	$NH_2Cl + HOCl \rightarrow NHCl_2 + H_2O$		22
S4	2.45×10^{-8}	s^{-1}	$NHCl_2 + H_2O \rightarrow NH_2Cl + HOCl$		21, 22
S5	k_{S5}[a]	$M^{-1}s^{-1}$	$NH_2Cl + NH_2Cl \rightarrow NHCl_2 + NH_3$		22
S6	6.00×10^{-3}	$M^{-1}s^{-1}$	$NHCl_2 + NH_3 \rightarrow 2\ NH_2Cl$		22
S7	2.00×10^{-7}	s^{-1}	$NHCl_2 + H_2O \rightarrow HNO$ + product		22
S8	2.78×10^{4}	$M^{-1}s^{-1}$	$NHCl_2 + HNO \rightarrow HOCl$ + product		22
S9	8.30×10^{3}	$M^{-1}s^{-1}$	$NH_2Cl + HNO \rightarrow$ product		22
S10	1.53×10^{-2}	$M^{-1}s^{-1}$	$NH_2Cl + NHCl_2 \rightarrow$ product		22
S11	3.30×10^{9}	$M^{-2}s^{-1}$	$HOCl + NHCl_2 + OH^- \rightarrow NCl_3 + OH- + H_2O$		22
S12	1.00×10^{5}	$M^{-2}s^{-1}$	$HOCl + NHCl_2 + OCl^- \rightarrow NCl_3 + OH^- + HOCl$		22
S13	1.60×10^{4}	$M^{-2}s^{-1}$	$HOCl + NHCl_2 + HPO_4^{2-} \rightarrow NCl_3 + OH^- + H_2PO_4^-$		22
S14	2.52×10^{2}	$M^{-1}s^{-1}$	$NCl_3 + NHCl_2 \rightarrow 2\ HOCl + N_2$	Rate constant at pH 7	3
S15	2.77×10^{2}	$M^{-1}s^{-1}$	$NCl_3 + NHCl_2 \rightarrow 2\ HOCl + N_2 + \bullet OH$	Rate constant at pH 7	3
S16	2.54×10^{2}	$M^{-1}s^{-1}$	$NCl_3 + NHCl_2 \rightarrow NHCl_2 + ClNO + H^+ + Cl^-$	Rate constant at pH 7	3
S17	3.10×10^{0}	$M^{-1}s^{-1}$	$NCl_3 + NH_2Cl \rightarrow HOCl$ + product	Rate constant at pH 7	3
S18	1.00×10^{-3}	s^{-1}	$H_2O \rightarrow H^+ + OH^-$	pK_a for H_2O at 25 °C = 14	3
S19	1.00×10^{11}	$M^{-1}s^{-1}$	$H^+ + OH^- \rightarrow H_2O$	pK_a for H_2O at 25 °C = 14	3
S20	1.41×10^{3}	s^{-1}	$HOCl \rightarrow OCl^- + H^+$	pK_a for HOCl at 25 °C = 7.5	3
S21	5.00×10^{10}	$M^{-1}s^{-1}$	$OCl^- + H^+ \rightarrow HOCl$	pK_a for HOCl at 25 °C = 7.5	3
S22	5.00×10^{10}	$M^{-1}s^{-1}$	$H^+ + Cl^- \rightarrow HCl$	pK_a for HCl at 25 °C = -6.2	3
S23	8.60×10^{16}	s^{-1}	$HCl \rightarrow H^+ + Cl^-$	pK_a for HCl at 25 °C = -6.2	3
S24	2.51×10^{1}	s^{-1}	$NH_4^+ \rightarrow NH_3 + H^+$	pK_a for NH_4^+ at 25 °C = 9.3	3
S25	5.00×10^{10}	$M^{-1}s^{-1}$	$NH_3 + H^+ \rightarrow NH_4^+$	pK_a for NH_4^+ at 25 °C = 9.3	3
Chemical reactions for chlorine-cyanuric acid interactions					
S26	3.17×10^{3}	s^{-1}	$Cl_3Cy + H_2O \rightarrow HCl_2Cy + HOCl$	pK_1 = 1.8; see Figure S4	23
S27	2.00×10^{5}	$M^{-1}s^{-1}$	$HCl_2Cy + HOCl \rightarrow Cl_3Cy + H_2O$	Assumed. See Text S4	
S28	1.78×10^{6}	s^{-1}	$HCl_2Cy \rightarrow Cl_2Cy^- + H^+$	pK_2 = 3.75; see Figure S4	23
S29	1.00×10^{10}	$M^{-1}s^{-1}$	$Cl_2Cy^- + H^+ \rightarrow HCl_2Cy$	Assumed	
S30	4.68×10^{4}	s^{-1}	$H_2ClCy \rightarrow HClCy^- + H^+$	pK_4 = 5.33; see Figure S4	23
S31	1.00×10^{10}	$M^{-1}s^{-1}$	$HClCy^- + H^+ \rightarrow H_2ClCy$	Assumed	
S32	1.32×10^{3}	s^{-1}	$H_3Cy \rightarrow H_2Cy^- + H^+$	pK_6 = 6.88; see Figure S4	23
S33	1.00×10^{10}	$M^{-1}s^{-1}$	$H_2Cy^- + H^+ \rightarrow H_3Cy$	Assumed	
S34	1.55×10^{1}	s^{-1}	$Cl_2Cy^- + H_2O \rightarrow HClCy^- + HOCl$	pK_7 = 4.1; see Figure S4	24
S35	2.00×10^{5}	$M^{-1}s^{-1}$	$HClCy^- + HOCl \rightarrow Cl_2Cy^- + H_2O$	Assumed. See Text S4	
S36	7.59×10^{-1}	s^{-1}	$HClCy^- \rightarrow ClCy^{2-} + H^+$	pK_8 = 10.12; see Figure S4	23
S37	1.00×10^{10}	$M^{-1}s^{-1}$	$ClCy^{2-} + H^+ \rightarrow HClCy^-$	Assumed	
S38	3.98×10^{-2}	s^{-1}	$H_2Cy^- \rightarrow HCy^{2-} + H^+$	pK_{10} = 11.4; see Figure S4	23
S39	1.00×10^{10}	$M^{-1}s^{-1}$	$HCy^{2-} + H^+ \rightarrow H_2Cy^-$	Assumed	
S40	2.72	s^{-1}	$ClCy^{2-} + H_2O \rightarrow HCy^{2-} + HOCl$	pK_{11} = 6.9; see Figure S4	23

S41	2.16×10^{7}	$M^{-1}s^{-1}$	$HCy^{2-} + HOCl \rightarrow ClCy^{2-} + H_2O$		[25]
S42	3.16×10^{-4}	s^{-1}	$HCy^{2-} \rightarrow Cy^{3-} + H^+$	pK_{12} = 13.5; see Figure S4	[23]
S43	1.00×10^{10}	$M^{-1}s^{-1}$	$Cy^{3-} + H^+ \rightarrow HCy^{2-}$	Assumed	
S44	1.15	s^{-1}	$HClCy^- + H_2O \rightarrow H_2Cy^- + HOCl$	pK_9 = 4.8; see Figure S4	[26]
S45	7.27×10^{4}	$M^{-1}s^{-1}$	$H_2Cy^- + HOCl \rightarrow HClCy^- + H_2O$		[25]
S46	235	s^{-1}	$HCl_2Cy + H_2O \rightarrow H_2ClCy + HOCl$	pK_3 = 2.93; see Figure S4	[23]
S47	2.00×10^{5}	$M^{-1}s^{-1}$	$H_2ClCy + HOCl \rightarrow HCl_2Cy + H_2O$	Assumed. See Text S4	
S48	25.8	s^{-1}	$H_2ClCy + H_2O \rightarrow H_3Cy + HOCl$	pK_5 = 4.1; see Figure S4	[23]
S49	2.00×10^{5}	$M^{-1}s^{-1}$	$H_3Cy + HOCl \rightarrow H_2ClCy + H_2O$	Assumed. See Text S4	
Radical scavenging reactions and others					
S50	1.21×10^{9}	$M^{-1}s^{-1}$	•OH + HOCl → ClO•		[27]
S51	6.37×10^{9}	$M^{-1}s^{-1}$	•OH + OCl^- → ClO•		[27]
S52	1.02×10^{9}	$M^{-1}s^{-1}$	•OH + NH_2Cl → product		[1]
S53	6.21×10^{8}	$M^{-1}s^{-1}$	•OH + $NHCl_2$ → product		[1]
S54	7.10×10^{9}	$M^{-1}s^{-1}$	•OH + DEET → product		[12]
S55	3.15×10^{9}	$M^{-1}s^{-1}$	•OH + 1,4-dioxane → product		[5]
S56	5.80×10^{9}	$M^{-1}s^{-1}$	•OH + benzoic acid → product		[8-11]
S57	4.00×10^{9}	$M^{-1}s^{-1}$	•OH + nitrobenzene → product		[6, 8, 9]
S58	6.40×10^{9}	$M^{-1}s^{-1}$	•OH + caffeine → product		[12]
S59	1.03×10^{8}	$M^{-1}s^{-1}$	ClO• + caffeine → product		[12]
S60	5.77×10^{5}	$M^{-1}s^{-1}$	ClNO + H_3Cy → product		This study
S61	5.77×10^{5}	$M^{-1}s^{-1}$	ClNO + H_2Cy^- → product		This study
S62	$5.8–68\times10^{5}$	$M^{-1}s^{-1}$	ClNO + HOCl → product		This study
S63	$5.8–68\times10^{5}$	$M^{-1}s^{-1}$	ClNO + OCl^- → product		This study
S64	6.79×10^{6}	$M^{-1}s^{-1}$	ClNO + Cl-DMA → product		This study
S65	3.15×10^{6}	$M^{-1}s^{-1}$	HOCl + DPD → DPD^+		[28]
S66	1.90×10^{7}	$M^{-1}s^{-1}$	H_3Cy + •OH → product		[20]
S67	1.90×10^{7}	$M^{-1}s^{-1}$	H_2Cy^- + •OH → product		[20]
S68	1.90×10^{7}	$M^{-1}s^{-1}$	$HClCy^-$ + •OH → product		[20]

[a] $k_{S5} = k_{H+}[H^+] + k_{H3PO4}[H_3PO_4] + k_{H2PO4-}[H_2PO_4^-] + k_{H3Cy}[H_3Cy] + k_{H2Cy-}[H_2Cy^-]$; see Table S6 for the specific rate constant for acid species.

Note: A reversible loop is a sequence of reversible reactions that together form a closed thermodynamic cycle. In principle, the product of the equilibrium constants for all steps in such a loop should equal unity. A substantial deviation from unity indicates an inconsistent, or "bad," reversible loop. For instance, reactions S1 and S2, S3 and S4, and S5 and S6 each represent reversible pairs that collectively form a closed thermodynamic cycle. Using previously reported constants,[22] the product of their equilibrium constants deviates markedly from one.

Text S3. Sensitivity of free chlorine predictions to reported K_7 and K_9 values in the chlorine–cyanuric acid system

Free chlorine (HOCl and OCl^-) reacts reversibly with cyanuric acid (H_3Cy) to form a series of chlorinated cyanurates, resulting in dynamic equilibria among 12 chemical species. These include unchlorinated cyanurates (H_3Cy, H_2Cy^-, HCy^{2-}, and Cy^{3-}), free chlorine (HOCl, OCl^-), and six chlorinated cyanurates (H_2ClCy, $HClCy^-$, $ClCy^{2-}$, HCl_2Cy, Cl_2Cy^-, and Cl_3Cy), as illustrated in Figure S4. These equilibria are pH-dependent and are also influenced by the relative concentrations of cyanuric acid and chlorine.

Accurate prediction of free chlorine concentration under typical pool conditions (e.g., near-neutral pH and sub-mM chlorine levels) depends heavily on the hydrolytic dissociation constants of dichlorocyanurate (Cl_2Cy^-, pK_7) and monochlorocyanurate ($HClCy^-$, pK_9). However, reported values of pK_7 and pK_9 in the literature vary significantly by over one log unit, leading to more than fivefold differences in modeled free chlorine concentrations.

O'Brien et al.[23] used UV-Vis spectrophotometry and electrometric titrations to determine equilibrium constants for 10 cyanurate species, reporting pK_7 = 4.51 and pK_9 = 5.62. Pinsky and Hu[24] later argued that the method and conditions used in O'Brien et al.'s study[23] may be biased because of spectral overlap with cyanuric acid at mM concentrations they employed in their experiments. Using linear sweep voltammetry, Pinsky and Hu[24] revised these values to 4.11 and 4.92, respectively. These values are shown in Figure S4. Jensen and Johnson[26] suggested that interference from chlorinated cyanurates may have affected Pinsky and Hu's measurements,[24] and re-evaluated pK_9 as 4.8 using an amperometric membrane electrode.

To evaluate the impact of these discrepancies, we used a chemical equilibrium model incorporating K_1–K_6, K_8, K_{10}–K_{12}, and K_{HOCl} from O'Brien et al.,[23] while substituting different literature values for K_7 and K_9. Figure S5 illustrates the resulting variation in equilibrium concentrations for free chlorine and chlorinated cyanurates at pH 7.0, under conditions of 100 µM free chlorine and 100 or 200 µM cyanuric acid. The predicted free chlorine concentrations vary by more than fivefold, demonstrating the critical role of accurate K_7 and K_9 values in modeling the concentration of HOCl/OCl^- in cyanuric acid-chlorine systems. In fact, the debate over the accuracy of hydrolytic dissociation constants persists in recent studies,[29, 30] leaving the accuracy of pK_7 and pK_9 values an open question.

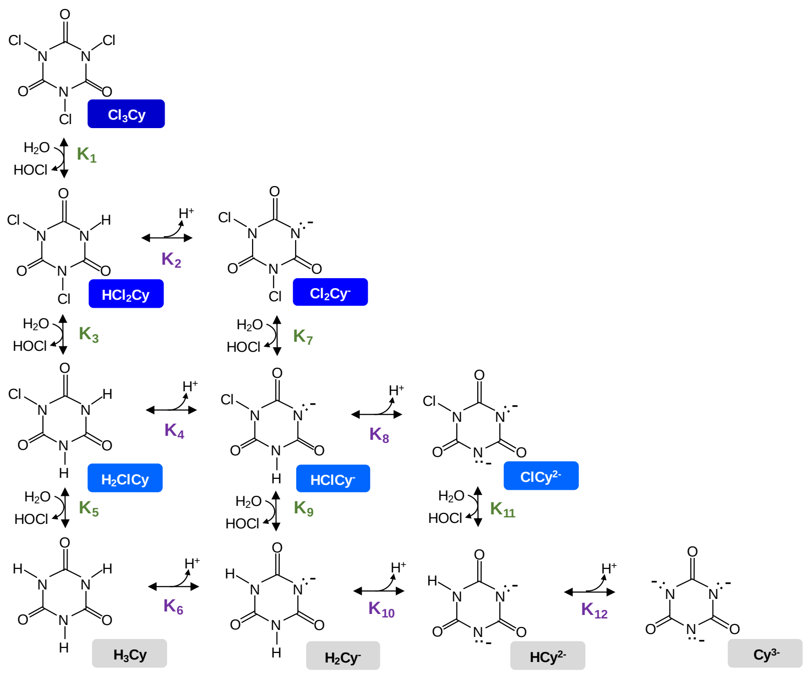

Reactions	Constant	pK (method; reference)
$Cl_3Cy + H_2O \leftrightarrow HCl_2Cy + HOCl$	K_1	1.80 (spectrometry; 23)
$HCl_2Cy \leftrightarrow Cl_2Cy^- + H^+$	K_2	3.75 (electrometric titration; 23)
$H_2ClCy + H_2O \leftrightarrow H_2ClCy + HOCl$	K_3	2.93 (spectrometry; 23)
$H_2ClCy \leftrightarrow HClCy^- + H^+$	K_4	5.33 (spectrometry; 23)
$H_2ClCy + H_2O \leftrightarrow H_3Cy + HOCl$	K_5	4.10 (spectrometry; 23)
$H_3Cy^- \leftrightarrow H_2Cy^- + H^+$	K_6	6.88 (spectrometry; 23)
$Cl_2Cy^- + H_2O \leftrightarrow HClCy^- + HOCl$	K_7	4.51 (spectrometry; 23)
		4.86 (spectrometry; 31)
		4.80 (titration, 32)
		4.11 (linear sweep voltammetry; 24)
$HClCy^- \leftrightarrow ClCy^{2-} + H^+$	K_8	10.1 (electrometric titration; 23)
$HClCy^- + H_2O \leftrightarrow H_2Cy^- + HOCl$	K_9	5.62 (spectrometry; 23)
		5.99 (spectrometry; 31)
		5.40 (titration, 32)
		4.92 (linear sweep voltammetry; 24)
		4.80 (electrochemical method; 26)
$H_2Cy^- \leftrightarrow HCy^{2-} + H^+$	K_{10}	11.4 (spectrometry; 23)
$ClCy^{2-} + H_2O \leftrightarrow HCy^{2-} + HOCl$	K_{11}	6.90 (spectrometry; 23)
$HCy^{2-} \leftrightarrow Cy^{3-} + H^+$	K_{12}	13.5 (spectrometry; 23)
$HOCl \leftrightarrow OCl^- + H^+$	K_{HOCl}	7.54 (titration; 33)

Figure S4. Equilibria among cyanuric acid species, chlorinated cyanurates, and free chlorine, and the corresponding reactions, equilibrium constants and hydrolytic dissociation constants reported in the literature. The reported K were values at 22–25 °C.

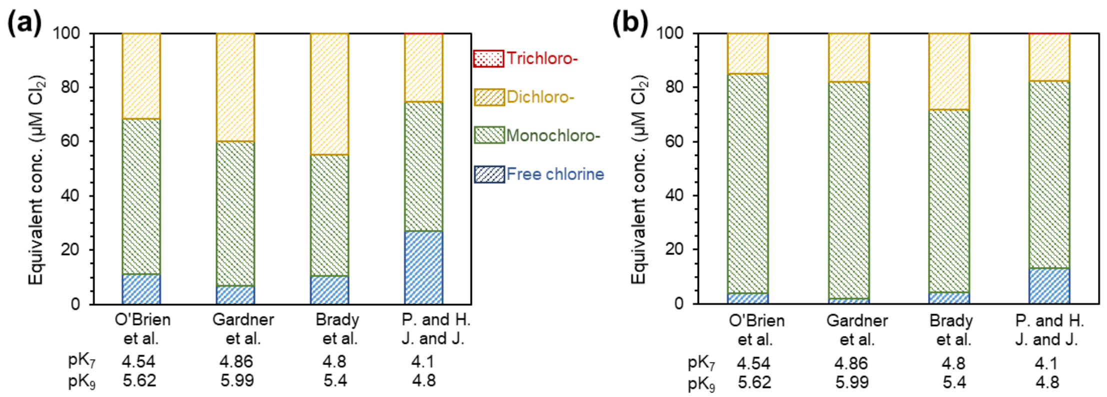

Figure S5. Equilibrium concentrations of free chlorine and Cl-cyanurates calculated using pK_7 and pK_9 reported in the literature. (a) $[\text{chlorine}]_{total}$ = 100 µM and $[\text{cyanuric acid}]_{total}$ = 100 µM. (b) $[\text{chlorine}]_{total}$ = 100 µM and $[\text{cyanuric acid}]_{total}$ = 200 µM. 'P. and H.' denotes Pinsky and Hu,[24] and 'J. and J.' refers to Jensen and Johnson.[26] Monochloro-cyanurates include H_2ClCy, $HClCy^-$, and $ClCy^{2-}$. Dichloro-cyanurates include HCl_2Cy and Cl_2Cy^-. Trichloro-cyanurate denotes Cl_3Cy.

Table S5. Bimolecular rate constants for the reaction of HOCl/OCl$^-$ with phenols.

	pK_a	$k_{HOCl, PhOH}$	k_{HOCl, PhO^-} ($M^{-1}s^{-1}$)
Phenol	9.99	0.36±0.28 [(b)]	$2.61(\pm0.26)\times10^4$ [(a)]
p-Chlorophenol	8.56	-	$3.58(\pm0.23)\times10^3$ [(a)]

(a) Ref: Lau et al.[34] (b) Ref: Gallard and von Gunten[35]

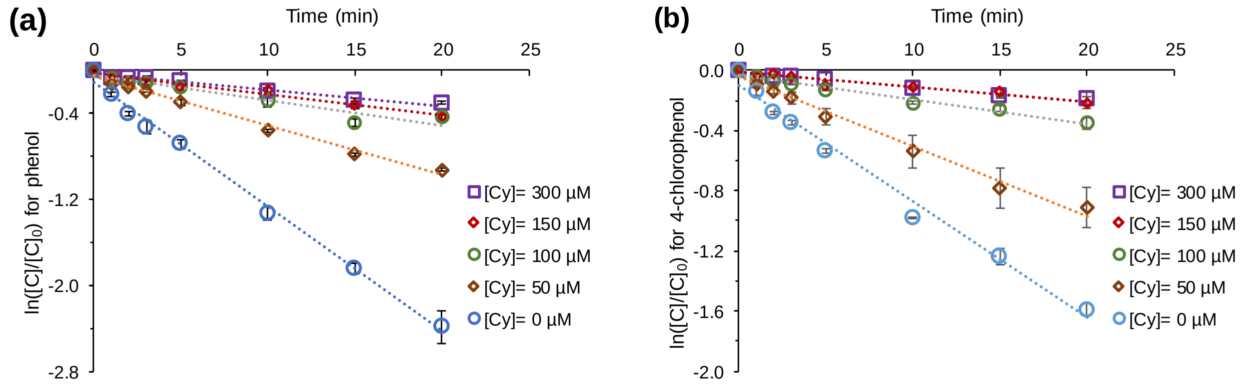

Figure S6. Plots of ln([C]/[C]$_0$) against reaction time for (a) phenol or (b) *p*-chlorophenol during the treatments of phenol or *p*-chlorophenol at 2 µM with 100 µM HOCl and 0–300 µM cyanuric acid (Cy). Reactions were conducted at pH 7 (10 mM phosphate buffer) at 25±1 °C.

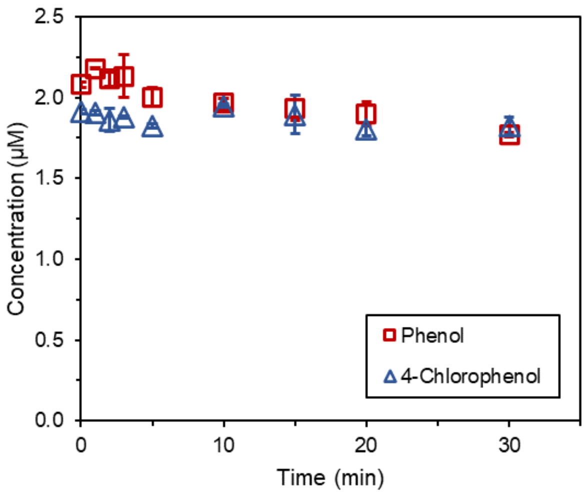

Figure S7. Time-dependent concentrations for phenol or 4-chlorophenol during the treatments of phenol or 4-chlorophenol at 2 µM with 50 µM HOCl and 1000 µM cyanuric acid. Reactions were conducted at pH 7 (10 mM phosphate buffer) at 25±1 °C. The 1000 µM cyanuric acid rendered more than 98% of the chlorine bound to it, forming chlorinated cyanurates at concentrations above 49 µM. Error bar represents data range from experimental duplicates.

Text S4. The rate constant for the reaction of cyanuric acid/cyanurate with HOCl in the kinetic model.

Matte et al.[25] reported bimolecular rate constants for the chlorination of H_2Cy^- and HCy^{2-} by HOCl, respectively. They reported k values of 7.27×10^4 $M^{-1}s^{-1}$ for H_2Cy^- and 2.16×10^7 $M^{-1}s^{-1}$ for HCy^{2-} (Reactions C2 and C3). Rate constants for the chlorination of H_3Cy, H_2ClCy, $HClCy^-$, and HCl_2Cy (Reactions C1, C4–C6) are not available in the literature. Note that these reactions (Reactions C1–C6) are the reverse of the hydrolytic dissociation reactions of the corresponding chlorinated cyanurates. With the knowledge of the equilibrium constant, the corresponding rate constant for the hydrolytic dissociation reaction can be calculated accordingly.

$H_3Cy + HOCl \rightarrow H_2ClCy + H_2O$	k_{C1}	(C1)
$H_2Cy^- + HOCl \rightarrow HClCy^- + H_2O$	$k_{C2} = 7.27\times10^4$ $M^{-1}s^{-1}$	(C2)
$HCy^{2-} + HOCl \rightarrow ClCy^{2-} + H_2O$	$k_{C3} = 2.16\times10^7$ $M^{-1}s^{-1}$	(C3)
$H_2ClCy + HOCl \rightarrow HCl_2Cy + H_2O$	k_{C4}	(C4)
$HClCy^- + HOCl \rightarrow Cl_2Cy^- + H_2O$	k_{C5}	(C5)
$HCl_2Cy + HOCl \rightarrow Cl_3Cy + H_2O$	k_{C6}	(C6)

To estimate rate constants for Reactions C1 and C4–C6, we leveraged kinetic modeling based on experimental data from Jensen and Johnson[36], who observed that the reaction between HOCl and DPD had a half-time of <0.4 s in HOCl–cyanuric acid solutions. This rapid consumption was similar to that observed without cyanuric acid, suggesting fast hydrolytic dissociation of chlorinated cyanurates to replenish free HOCl and maintain equilibrium during DPD reaction.

Given the known rate constant for HOCl + DPD ($k_{HOCl,\ DPD}$) is $3.15\pm0.03\times10^6$ $M^{-1}s^{-1}$,[28] and their experimental setup (pH 6.2, [DPD] = 1.09×10^{-4} M, [cyanuric acid] ~100×[HOCl], [HOCl] in the µM range), we modeled chlorine kinetics including reversible chlorination reactions for chlorinated cyanurates. By adjusting the assumed rate constant for Reactions C1 and C4–C6, we found that $k = 2\times10^5$ $M^{-1}s^{-1}$ produced a modeled half-time of 0.4 s (Figure S8). This value serves as a reasonable estimate for the average rate constant governing the conversion of HOCl to higher-order chlorinated cyanurates.

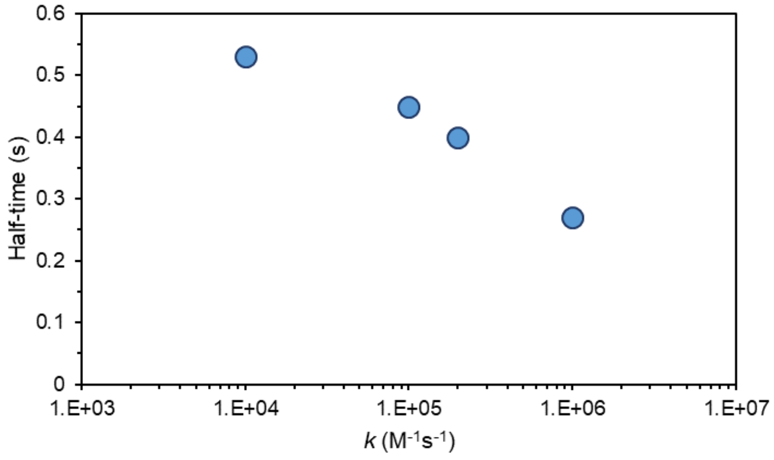

Figure S8. Modeled half-time for total chlorine depletion as a function of the assumed bimolecular rate constant (k) for reactions between HOCl and chlorinated cyanurates (Reactions C1 and C4–C6), under conditions consistent with Jensen and Johnson[36]: pH 6.2, [DPD] = 1.09×10^{-4} M, and a 100-fold excess of cyanuric acid over HOCl. A rate constant of 2×10^{5} $M^{-1}s^{-1}$ yields a half-time of 0.4 s, consistent with their experimental data.

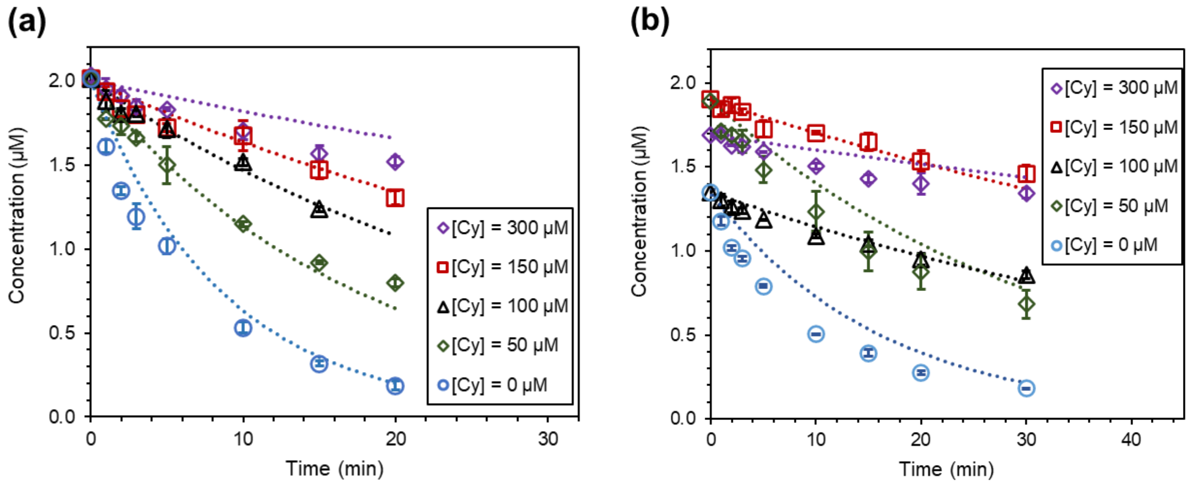

Figure S9. Time-dependent degradation of (a) phenol and (b) p-chlorophenol during the treatments of 2 μM phenols with 100 μM HOCl and 0–300 μM cyanuric acid (Cy). Symbols are experimental data, while lines represent the kinetic model simulations. Experiments were conducted at pH 7 with 10 mM phosphates. Error bar represents data range from experimental duplicates.

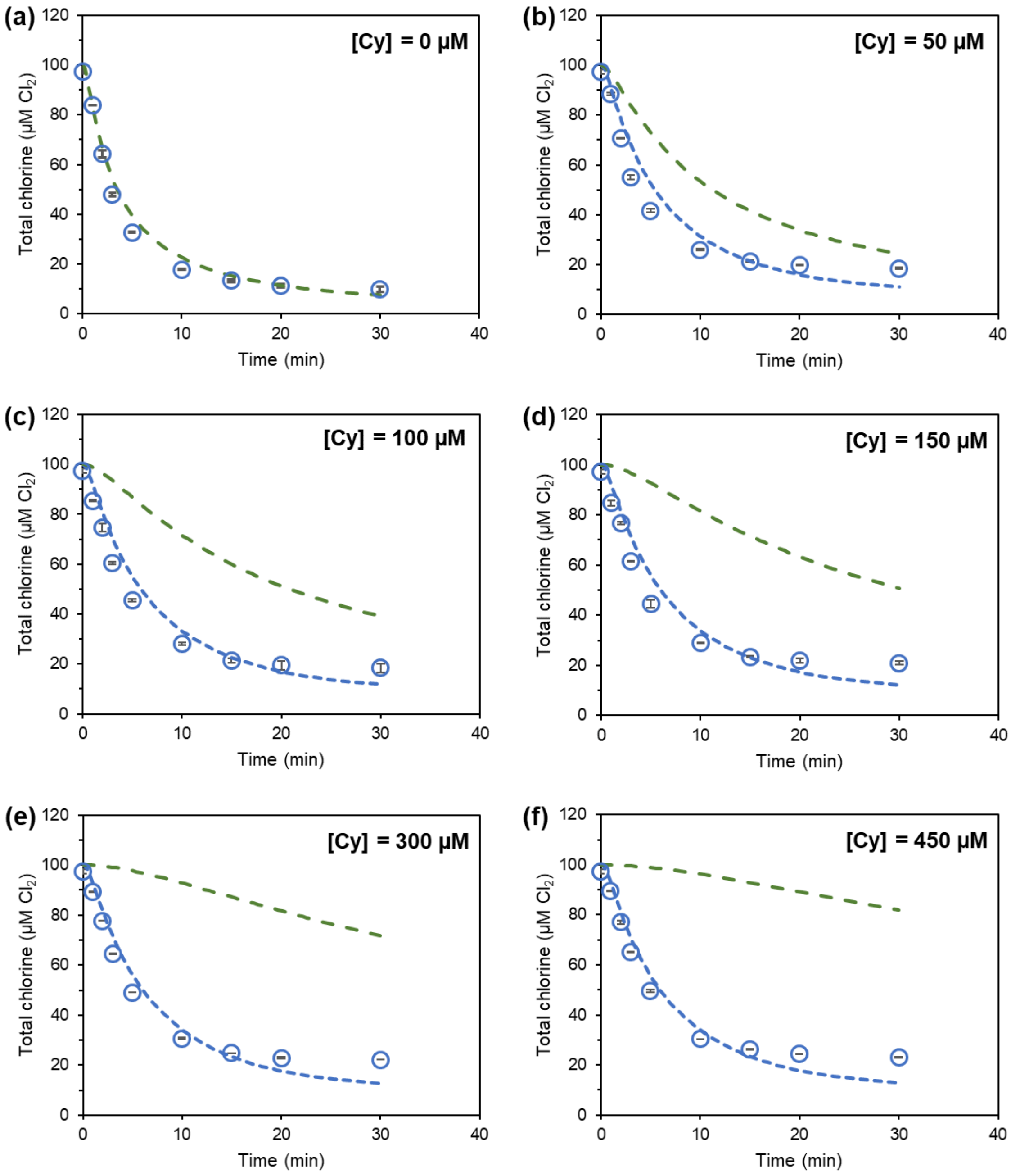

Figure S10. Experimental data for total chlorine concentration during the reaction of 100 μM HOCl with 50 μM NH_4^+ in the presence of 0–450 μM cyanuric acid (Cy). Green lines represent kinetic model simulations without considering the (chlorinated) cyanurates-ammonia/chloramines interaction (as listed in Table 1 in the main text), while the blue lines represent those with considering those interactions. Experiments were conducted at pH 7 in 10 mM phosphate buffer. Error bar represent the ranges from experimental duplicates. Experimental total chlorine concentrations were measured by the DPD method.

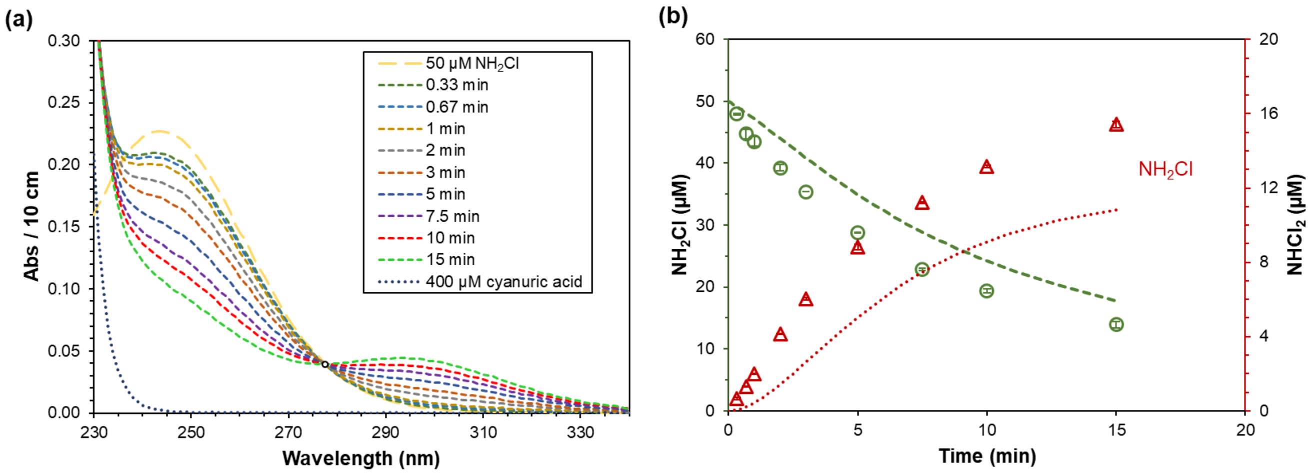

Figure S11. (a) Time-dependent UV spectra of a mixture containing 400 μM cyanuric acid in 10 mM phosphates at pH 6.0 treated with 50 μM NH_2Cl. The hollow circle denotes the isosbestic point. (b) Concentrations of NH_2Cl and $NHCl_2$ over time, determined by spectral deconvolution using the UV absorbance/simultaneous equations method. Error bar represents data range from experimental duplicates.

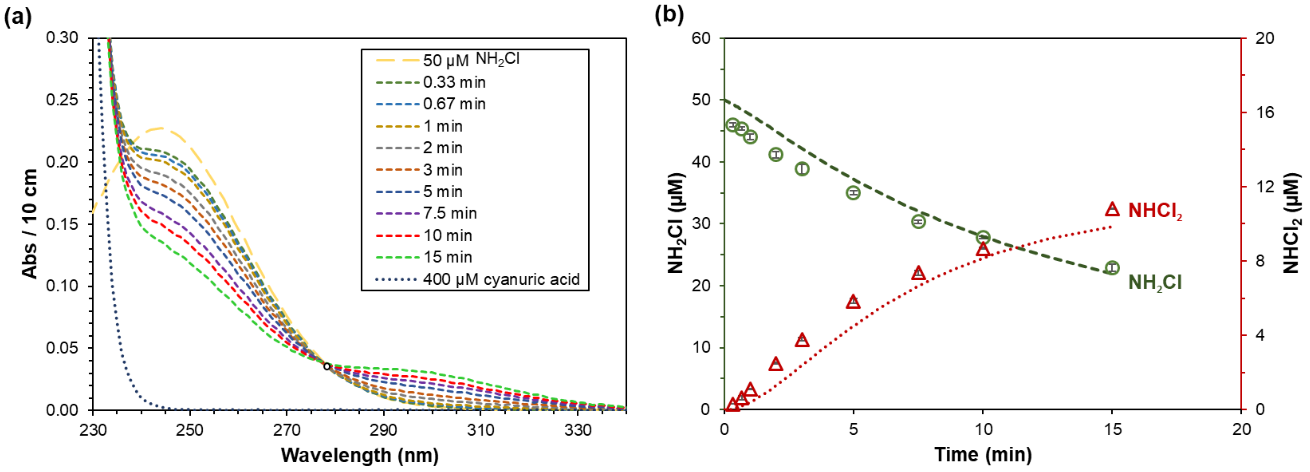

Figure S12. (a) Time-dependent UV spectra of a mixture containing 400 μM cyanuric acid in 10 mM phosphates at pH 6.5 treated with 50 μM NH_2Cl. The hollow circle denotes the isosbestic point. (b) Concentrations of NH_2Cl and $NHCl_2$ over time, determined by spectral deconvolution using the UV absorbance/simultaneous equations method. Error bar represents data range from experimental duplicates.

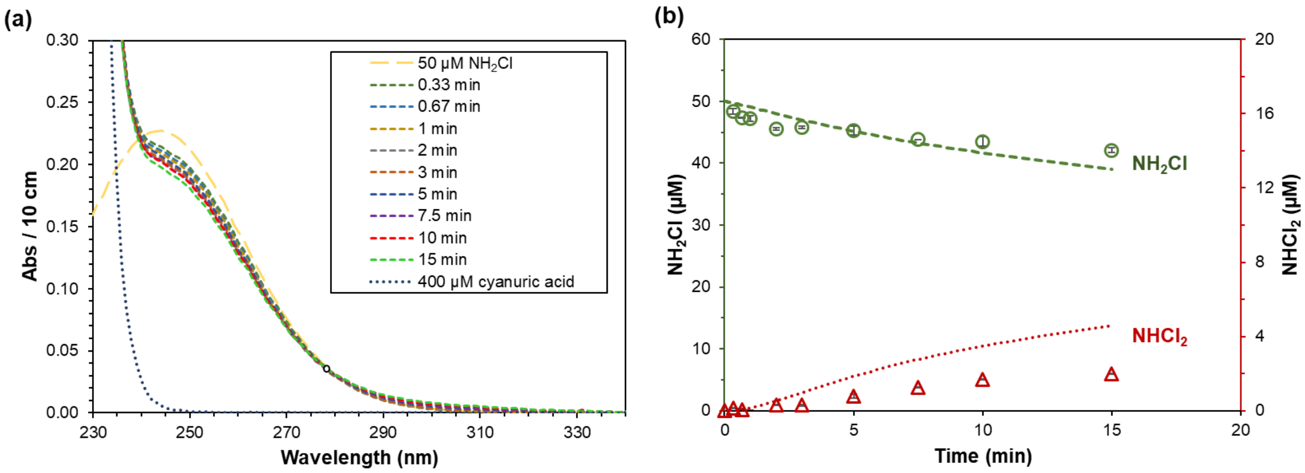

Figure S13. (a) Time-dependent UV spectra of a mixture containing 400 μM cyanuric acid in 10 mM phosphates at pH 7.5 treated with 50 μM NH_2Cl. The hollow circle denotes the isosbestic point. (b) Concentrations of NH_2Cl and $NHCl_2$ over time, determined by spectral deconvolution using the UV absorbance/simultaneous equations method. Error bar represents data range from experimental duplicates.

Text S5. Kinetics analyses of NH_2Cl decomposition during cyanuric acid-NH_2Cl interactions.

The decomposition of NH_2Cl to $NHCl_2$ occurs through the bimolecular pathway:

$$NH_2Cl + NH_2Cl \rightarrow NHCl_2 + NH_3$$

Accordingly, the rate of NH_2Cl disappearance is given by:

$$d[NH_2Cl]/dt = -2k[NH_2Cl]^2$$

where k is the apparent second-order rate constant. Integration of this expression with respect to time (t) yields:

$$1/[NH_2Cl]_t - 1/[NH_2Cl]_0 = 2kt$$

Here, $[NH_2Cl]_0$ and $[NH_2Cl]_t$ denotes the NH_2Cl concentrations at the initial time and at reaction time t, respectively.

To evaluate the kinetics from the experiments where 50 µM NH_2Cl at 10 mM phosphate-buffered at pH 7 treated by 400 µM cyanuric acid, plots of ($1/[NH_2Cl]_t - 1/[NH_2Cl]_0$) versus t were constructed for reactions conducted over the pH range 6.0–7.5. The resulting linear relationships confirm that NH_2Cl decomposition in the presence of cyanuric acid follows second-order kinetics under these conditions. The slope of the regression lines corresponds to $2k$, from which apparent rate constants (k_{app}) were determined. The calculated k_{app} values decreased systematically with increasing pH, ranging from 26.4±0.1 $M^{-1}s^{-1}$ at pH 6.0 to 1.7 ± 0.1 $M^{-1}s^{-1}$ at pH 7.5 in phosphate-buffered systems. These results demonstrate that the NH_2Cl self-reaction is strongly pH dependent, with faster decomposition occurring under more acidic conditions.

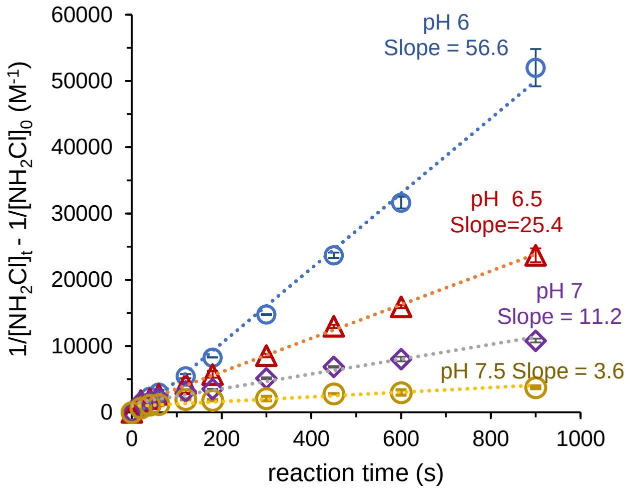

Figure S14. Second-order kinetic analysis of NH_2Cl decomposition during cyanuric acid (400 µM)–NH_2Cl (50 µM) interactions in 10 mM phosphate-buffered systems at pH 6–7.5. Plots of ($1/[NH_2Cl]_t - 1/[NH_2Cl]_0$) versus reaction time ($t$) yielded linear relationships across the tested pH range (6.0–7.5), confirming second-order kinetics. The slopes of the regression lines correspond to $2k$, from which apparent rate constants (k_{app}) were derived. Error bars represent data range from experimental duplicates.

Previous work by Valentine and Jafvert[37] demonstrated that NH_2Cl disproportionation to $NHCl_2$ can be catalyzed by general acids (Reaction S5 in Table S4). In this pathway, protonation of NH_2Cl produces NH_3Cl^+, which rapidly chlorinates a second NH_2Cl to generate $NHCl_2$, NH_3, and H^+. Accordingly, the overall second-order rate constant for NH_2Cl disproportionation (i.e., $d[NH_2Cl]/dt = k_{S5}\times[NH_2Cl]^2$) was expressed as the sum of contributions from proton-donating species. We extended this framework by recognizing that both H_3Cy and H_2Cy^- bear exchangeable protons and may therefore catalyze NH_2Cl disproportionation. Incorporating these terms yields:

$$k_{S5} = k_{H+}[H^+] + k_{H3PO4}[H_3PO_4] + k_{H2PO4-}[H_2PO_4^-] + k_{H3Cy}[H_3Cy] + k_{H2Cy-}[H_2Cy^-]$$

The k_{H3Cy} and k_{H2Cy-} can be estimated using the linear free-energy relationships (LFER) developed in Valentine and Jafvert's work,[37] which relate the specific rate constant of a proton donor to its pK_a, the number of exchangeable protons, and the binding capacity of its conjugate base:

$$\log (k_i/P) = C_1[pK_a + \log (P/Q)] + C_2$$

Here, k_i is the specific rate constant for acid species i, P is the number of exchangeable protons for acid species i, and Q is the maximum number of protons that the conjugate base of specie i could combine with. C_1(-0.68) and C_2(4.09) are constants. Table S6 shows the calculated specific rate constants for different acids.

Table S6. Calculation of the specific rate constant for acids using the linear free energy relationships developed by Valentine and Jafvert[37].

Acid species i	pK_a	P	Q	P/Q	pK_a+log(P/Q)	Calculated using $\log (k_i/P) = C_1[pK_a + \log (P/Q)] + C_2$		Literature reported (ref.) k_i ($M^{-2}s^{-1}$)
						Log(k_i/P)	k_i ($M^{-2}s^{-1}$)	
H^+	0	1	1	1	0.0	4.1	1.2×10^4	6.4×10^3 (37)
HSO_4^-	2	1	2	0.5	1.7	2.9	8.6×10^2	1.7×10^3 (37)
H_3PO_4	2.1	3	1	3	2.6	2.3	6.5×10^2	8.7×10^2 (37)
$H_2PO_4^-$	7.2	2	2	1	7.2	-0.8	3.1×10^{-1}	3.9×10^{-1} (37)
H_3Cy	6.88	3	1	3	7.4	-0.9	3.7×10^{-1}	N.A.
H_2Cy^-	11.4	2	2	1	11.4	-3.7	4.4×10^{-4}	N.A.
H_2CO_3	6.4	2	1	2	6.7	-0.5	6.8×10^{-1}	1.1×10^1 (38)
HCO_3^-	10.4	1	2	0.5	10.1	-2.8	1.7×10^{-3}	2.2×10^{-1} (38)

The resulting values, 3.7×10^{-1} $M^{-2}s^{-1}$ for H_3Cy and 4.4×10^{-4} $M^{-2}s^{-1}$ for H_2Cy^-, were on par with those for H_2CO_3 (6.8×10^{-1} $M^{-2}s^{-1}$ $M^{-2}s^{-1}$) and HCO_3^- (1.7×10^{-3} $M^{-2}s^{-1}$) calculated using the LFER. Notably, the LFER-derived values for $H_2CO_3$and HCO_3^- are themselves one to two orders of magnitude lower than experimentally determined rate constants.[38]

When the concentrations of each acid were substituted into the expanded expression for k_{S5} ($k_{S5} = k_{H+}[H^+] + k_{H3PO4}[H_3PO_4] + k_{H2PO4-}[H_2PO_4^-] + k_{H3Cy}[H_3Cy] + k_{H2Cy-}[H_2Cy^-]$) under our experimental conditions, the calculated k_{S5} values (1.5×10^{-3}–1.6×10^{-2} $M^{-1}s^{-1}$, see Table S7) were 3 to 4 orders of magnitude lower than the experimental k_{app} values obtained in the NH_2Cl–cyanuric acid systems (1.7–26.4 $M^{-1}s^{-1}$). This large discrepancy indicates that acid-catalyzed disproportionation alone cannot explain the rapid NH_2Cl decay observed in the presence of cyanuric acid.

To test this further, we performed control experiments in carbonate buffer (pH 7), where the H_2CO_3 concentration (173 µM) matched the H_3Cy concentration present in NH_2Cl–cyanuric acid experiments (50 µM NH_2Cl and 400 µM cyanuric acid at pH 7, see Table S7). Under these conditions, NH_2Cl remained essentially stable over 20 min (Figure S15), in stark contrast to the rapid decay observed in the presence of cyanuric acid

(Figure 2 in the main text). These findings demonstrate that cyanuric acid promotes NH_2Cl conversion through additional pathways beyond simple acid catalysis.

Table S7. Calculated k_{S5} values (rate constant for the NH_2Cl disproportionation reaction) under experimental conditions in this study. The subscript "eq." denotes equilibrium concentrations under our experimental conditions.

Experimental conditions								
pH	$[NH_2Cl]_{initial}$ (μM)	[Cyanuric acid]$_{initial}$ (μM)	$[H_3Cy]_{eq.}$ (M)	$[H_2Cy^-]_{eq}$ (M)	$[H_3PO_4]_{eq.}$ (M)	$[H_2PO_4^-]_{eq.}$ (M)	Calculated k_{S5} ($M^{-1}s^{-1}$)	Exp. observed k_{app} ($M^{-1}s^{-1}$)
6	50	400	3.5×10^{-4}	4.7×10^{-5}	1.2×10^{-6}	9.4×10^{-3}	1.6×10^{-2}	26.4±0.1
6.5	50	400	2.8×10^{-4}	1.2×10^{-4}	3.3×10^{-7}	8.3×10^{-3}	6.8×10^{-3}	12.6±0.0
7	50	400	1.7×10^{-4}	2.3×10^{-4}	7.7×10^{-8}	6.1×10^{-3}	3.3×10^{-3}	5.6±0.0
7.5	50	400	7.7×10^{-5}	3.2×10^{-4}	1.3×10^{-8}	3.3×10^{-3}	1.5×10^{-3}	1.7±0.1

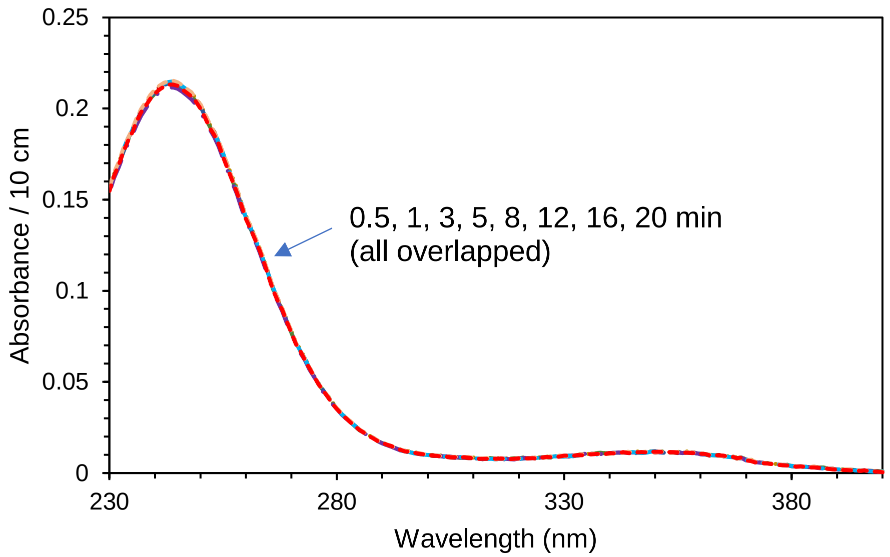

Figure S15. Time-dependent UV spectra of a mixture containing 400 μM cyanuric acid in 1 mM carbonates at pH 7 treated with 50 μM NH_2Cl. Under this condition, the H_2CO_3 concentration was 173 μM. The spectra can be compared to Figure 2 in the main text.

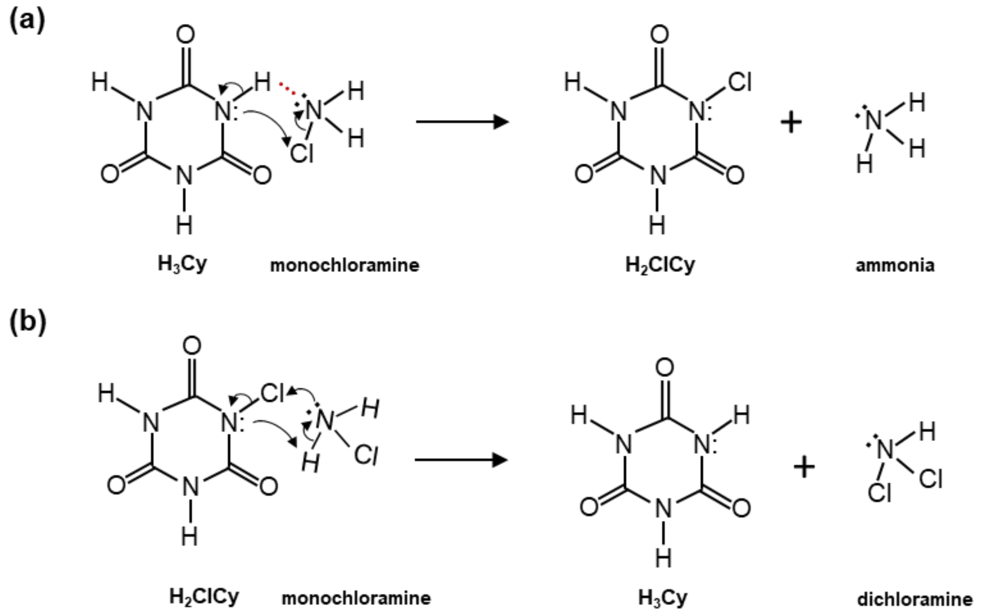

Scheme S1. Plausible mechanism by which H_3Cy catalyzes Cl(+1) transfer, facilitating the formation of $NHCl_2$ from two equivalents of NH_2Cl.

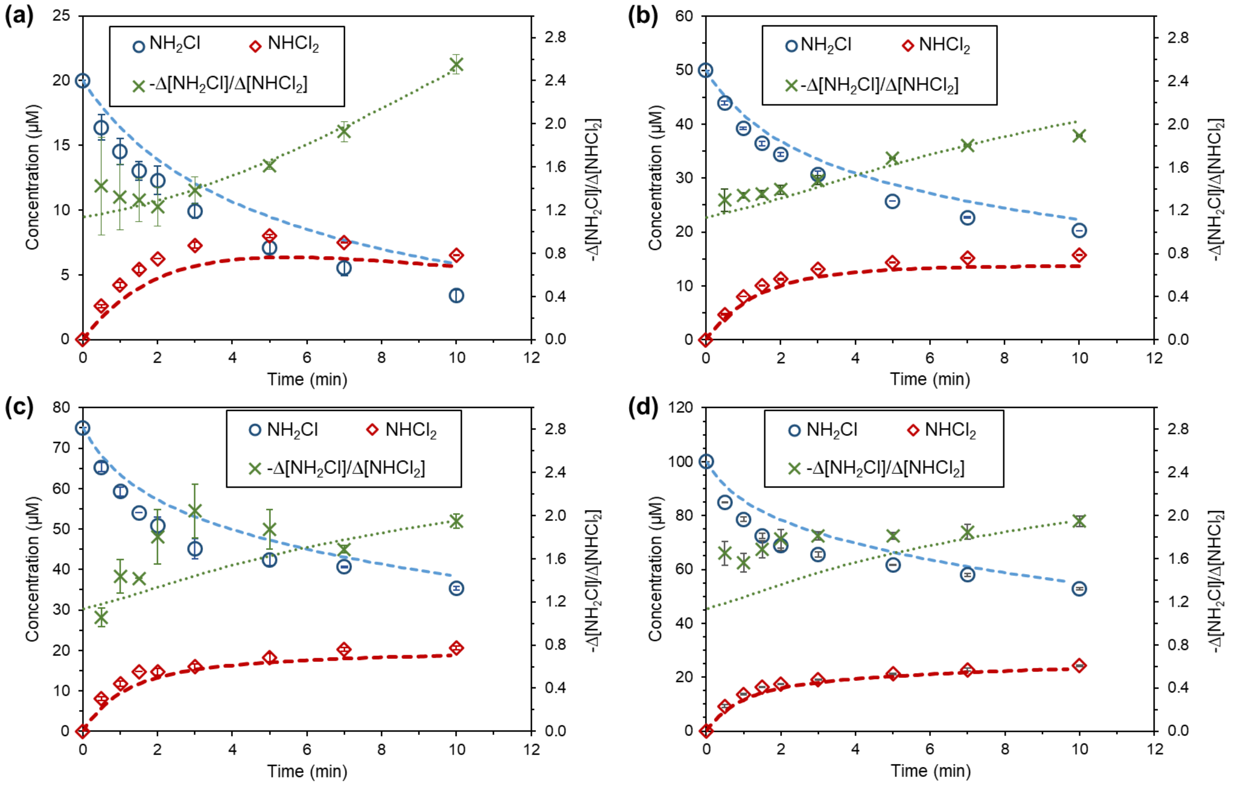

Figure S16. Concentration of NH_2Cl and $NHCl_2$ during the treatments of a mixture of 20 µM HOCl + 400 µM cyanuric acid in deionized water buffered at pH 7 by (a) 20 µM NH_2Cl, (b) 50 µM NH_2Cl, (c) 75 µM NH_2Cl, and (d) 100 µM NH_2Cl. Symbols indicate experimental data, and lines are kinetic model simulations. Error bar represent the ranges from experimental duplicates.

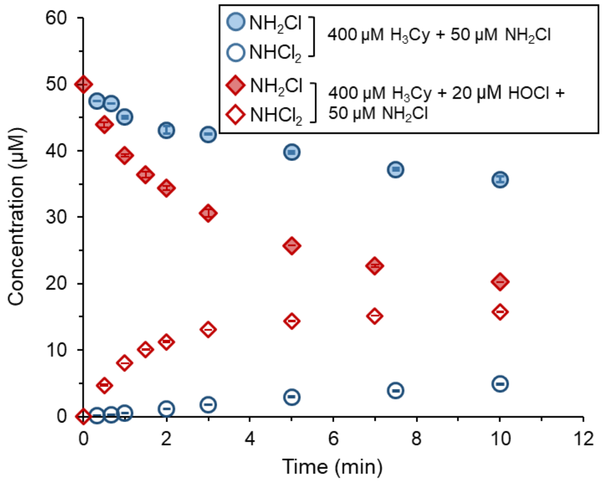

Figure S17. Concentration of NH_2Cl and $NHCl_2$ during the treatment of a mixture of 400 µM cyanuric acid in deionized water buffered at pH 7 by 50 µM NH_2Cl, or during the treatment of a mixture of 20 µM HOCl + 400 µM cyanuric acid (pre-equilibrated first) in deionized water buffered at pH 7 by 50 µM NH_2Cl. Error bar represent the ranges from experimental duplicates.

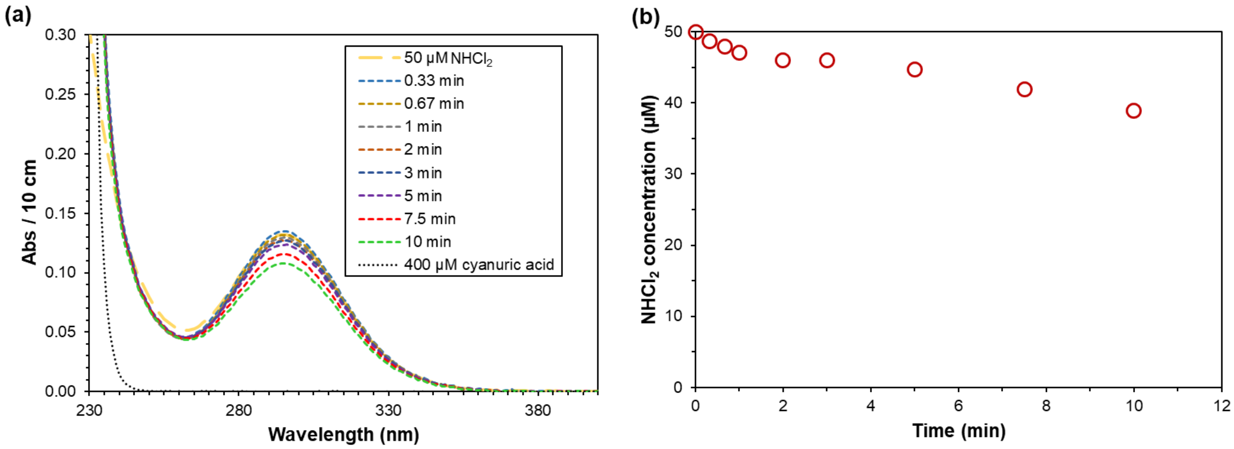

Figure S18. (a) Time-dependent UV spectra of a mixture containing 400 µM cyanuric acid in 20 mM phosphates at pH 7.0 treated with 50 µM $NHCl_2$. (b) Concentrations of $NHCl_2$ over time, determined using the UV absorbance/simultaneous equations method.

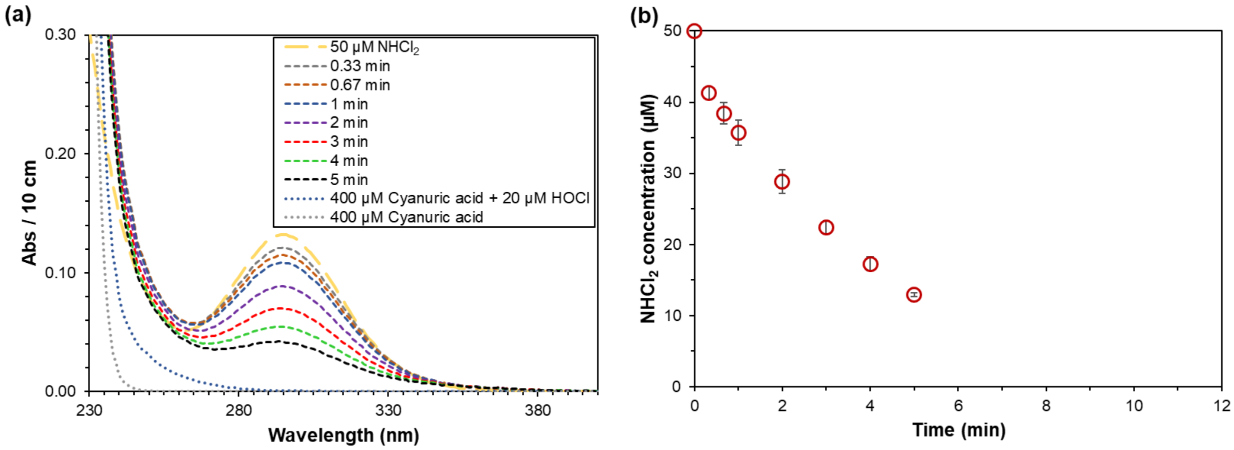

Figure S19. (a) Time-dependent UV spectra of a mixture containing 20 µM HOCl + 400 µM cyanuric acid (pre-equilibrated first) in 20 mM phosphates at pH 7.0 treated with 50 µM $NHCl_2$. (b) Concentrations of $NHCl_2$ over time, determined using the UV absorbance/simultaneous equations method. Error bar represents data range from experimental duplicates.

Text S6. Determination of rate constants for reactions between (chlorinated) cyanuric acid and chloramines.

For the experiments involving the reaction between chlorinated cyanurates and NH_2Cl (i.e., Exp. Nos.1, 5–10 in Table S2), a solution containing 20 µM HOCl pre-equilibrated with 400 µM cyanuric acid was treated by 20–125 µM NH_2Cl. The large excess of cyanuric acid relative to HOCl ensured that more than 96% of the chlorine remained cyanurate-bound, with $HClCy^-$ accounting for 93% of the total Cl_2 (i.e., $[HClCy^-]$ = 18.6 µM).

UV-spectra were taken periodically, and the concentrations of NH_2Cl and $NHCl_2$ were determined using the UV absorbance/simultaneous equations method as described in Text S1. Results were presented in Figure S16. The initial decay rate for NH_2Cl was calculated by $([NH_2Cl]_{initial} – [NH_2Cl]_{30\ sec})$/30 sec. Plotting the initial decay rates for NH_2Cl against $[NH_2Cl]_{initial} \times [HClCy^-]_{initial}$ from these experiments yielded linear lines (Figure S20a), with a slope of 166 $M^{-1}s^{-1}$, which is equivalent of the rate constants of k_{R3} ($HClCy^-$ + NH_2Cl) in the main text.

Similar approach was employed for the experimental data involving the reactions between chlorinated cyanurates and $NHCl_2$. Plotting the initial decay rates for $NHCl_2$ against $[NHCl_2]_{initial} \times [HClCy^-]_{initial}$ from these experiments yielded linear lines (Figure S20b), with a slope of 135 $M^{-1}s^{-1}$, which is equivalent of the rate constants of k_{R6} ($HClCy^-$ + NH_2Cl) in the main text.

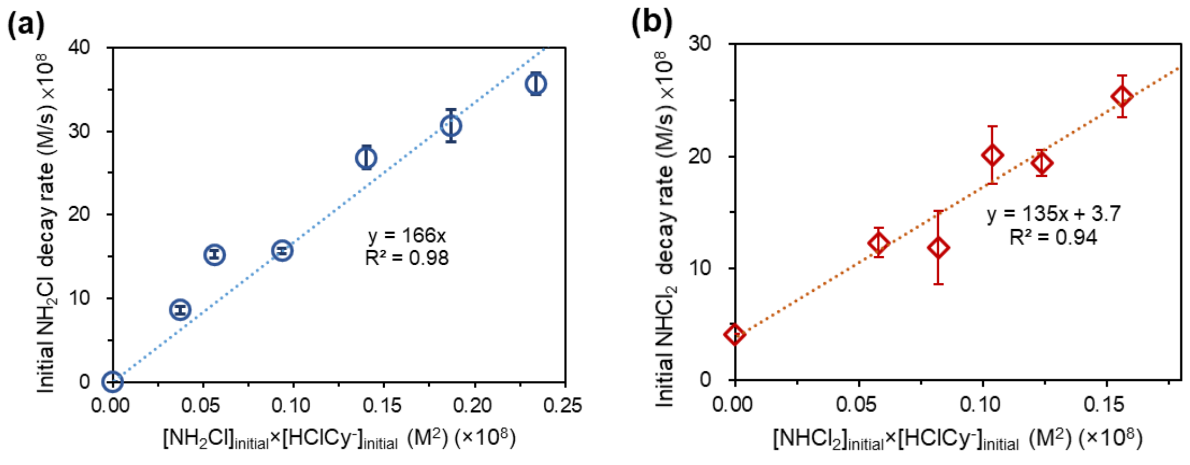

Figure S20. Relationships between initial decay rates for NH_2Cl or $NHCl_2$ and $[NH_2Cl]_{initial}$ (or $[NHCl_2]_{initial}$)$\times[HClCy^-]_{initial}$ from experiments with NH_2Cl or $NHCl_2$ in excess to $HClCy^-$. Experimental conditions were provided in Table S2. Error bar represent the ranges from experimental duplicates.

Text S7. Determination of species-specific reaction rate constants for k_{R1} and k_{R2}.

We fitted the experimental data for NH_2Cl concentration and for $NHCl_2$ formation at 15 min after the reactions, by implementing the kinetics model incorporating reactions R1–R6 in the main text. We performed two-variable data fitting to obtain the optimal k_{R1} and k_{R2} during the reaction between H_3Cy and NH_2Cl.

Table S8 summarizes the data fitting results. The optimal k_{R1} and k_{R2} were determined using least squares fitting, where the sum of squared relative error (SSRE) is defined as: $\mathrm{SSRE} = \sum\left[\left([\mathrm{NH_2Cl}]_{\mathrm{modeled}} - [\mathrm{NH_2Cl}]_{\mathrm{exp.}}\right)^2 + \left([\mathrm{NHCl_2}]_{\mathrm{modeled}} - [\mathrm{NHCl_2}]_{\mathrm{exp.}}\right)^2\right]$; $[NH_2Cl]_{exp.}$ and $[NH_2Cl]_{modeled}$ respectively represents the experimentally determined and modeled NH_2Cl concentration at 15 min, while $[NHCl_2]_{exp.}$ and $[NHCl_2]_{modeled}$ respectively represents the experimentally determined and modeled $NHCl_2$ concentration at 15 min. For example, for 400 µM H_3Cy with 50 µM NH_2Cl, implementing the kinetics model using [k_{R1} = 0.5 $M^{-1}s^{-1}$, k_{R1} = 0.01 $M^{-1}s^{-1}$] predicted $[NH_2Cl]$ and $[NHCl_2]$ concentration at 15 min were 3.2×10^{-5} M and 7.1×10^{-6} M, respectively, at pH 6.0, were 3.5×10^{-5} M and 6.4×10^{-6} M, respectively, at pH 6.5, were 3.8×10^{-5} M and 4.9×10^{-6} M, respectively, at pH 7.0, and 4.2×10^{-5} M and 3.3×10^{-6} M, respectively, at pH 7.5. This results in a SSRE of 6.1×10^{-10}.

We implemented kinetics simulation using k_{R1}=0.5 to 4 $M^{-1}s^{-1}$, with an interval of 0.05 $M^{-1}s^{-1}$, and k_{R2}=0.01, 0.05, or 0.1 $M^{-1}s^{-1}$; k_{R2} was found to be not sensitive to the modeling results. The results showed that a k_{R1} of 2 $M^{-1}s^{-1}$ and a k_{R2} of 0.01 resulted in a smallest SSRE.

Table S8. Comparison between experimentally observed and modeled concentrations of NH_2Cl and $NHCl_2$ at 15 min (unit: M), and the corresponding SSRE, using different k_{R1} and k_{R2} values (unit = $M^{-1}s^{-1}$).

Experimentally observed concentration at 15 min										
		pH 6		pH 6.5		pH 7		pH 7.5		
		[NH_2Cl]	[$NHCl_2$]	[NH_2Cl]	[$NHCl_2$]	[NH_2Cl]	[$NHCl_2$]	[NH_2Cl]	[$NHCl_2$]	
		1.4E-05	1.5E-05	2.3E-05	1.1E-05	3.2E-05	6.1E-06	4.2E-05	2.0E-06	
Modeled concentrations at 15 min										
		pH 6		pH 6.5		pH 7		pH 7.5		
k_{R1}	k_{R2}	[NH_2Cl]	[$NHCl_2$]	[NH_2Cl]	[$NHCl_2$]	[NH_2Cl]	[$NHCl_2$]	[NH_2Cl]	[$NHCl_2$]	SSRE
0.5	0.01	3.2E-05	7.1E-06	3.5E-05	6.4E-06	3.8E-05	4.9E-06	4.2E-05	3.3E-06	6.1E-10
1	0.01	2.6E-05	9.1E-06	2.9E-05	8.1E-06	3.5E-05	6.1E-06	4.1E-05	3.8E-06	2.4E-10
1.5	0.01	2.1E-05	1.0E-05	2.5E-05	9.2E-06	3.2E-05	7.0E-06	4.0E-05	4.2E-06	9.8E-11
2	0.01	1.8E-05	1.1E-05	2.2E-05	9.9E-06	3.0E-05	7.7E-06	3.9E-05	4.6E-06	**6.0E-11**
2.5	0.01	1.5E-05	1.1E-05	2.0E-05	1.0E-05	2.8E-05	8.2E-06	3.8E-05	4.9E-06	7.6E-11
3	0.01	1.3E-05	1.1E-05	1.8E-05	1.0E-05	2.7E-05	8.6E-06	3.7E-05	5.2E-06	1.2E-10
4	0.01	1.0E-05	1.1E-05	1.5E-05	1.1E-05	2.4E-05	9.1E-06	3.6E-05	5.7E-06	2.3E-10
0.5	0.05	3.2E-05	7.1E-06	3.4E-05	6.4E-06	3.8E-05	5.1E-06	4.2E-05	3.5E-06	6.0E-10
1	0.05	2.6E-05	9.1E-06	2.9E-05	8.2E-06	3.5E-05	6.2E-06	4.1E-05	3.9E-06	2.4E-10
2	0.05	1.8E-05	1.1E-05	2.2E-05	9.9E-06	3.0E-05	7.7E-06	3.9E-05	4.7E-06	6.4E-11
3	0.05	1.3E-05	1.1E-05	1.7E-05	1.0E-05	2.7E-05	8.6E-06	3.7E-05	5.3E-06	1.2E-10
4	0.05	1.0E-05	1.1E-05	1.5E-05	1.1E-05	2.4E-05	9.2E-06	3.6E-05	5.7E-06	2.4E-10
0.5	0.1	3.2E-05	7.2E-06	3.4E-05	6.5E-06	3.8E-05	5.3E-06	4.1E-05	3.7E-06	5.8E-10
1	0.1	2.6E-05	9.2E-06	2.9E-05	8.2E-06	3.4E-05	6.4E-06	4.0E-05	4.1E-06	2.3E-10
2	0.1	1.8E-05	1.1E-05	2.2E-05	9.9E-06	3.0E-05	7.8E-06	3.8E-05	4.8E-06	6.9E-11
3	0.1	1.3E-05	1.1E-05	1.7E-05	1.0E-05	2.7E-05	8.7E-06	3.7E-05	5.4E-06	1.3E-10
4	0.1	1.0E-05	1.1E-05	1.5E-05	1.1E-05	2.4E-05	9.2E-06	3.5E-05	5.8E-06	2.4E-10

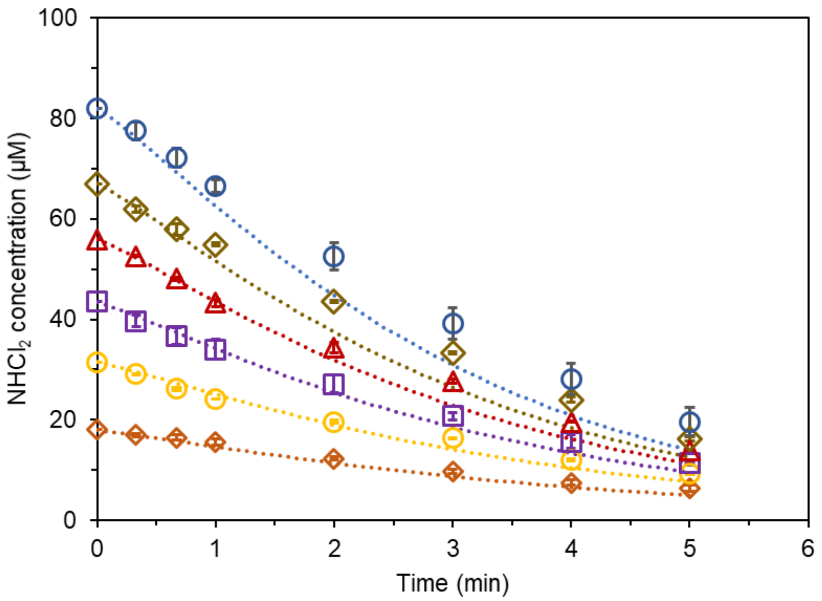

Figure S21. $NHCl_2$ concentration during the treatments of a solution containing 400 µM cyanuric acid and 20 µM HOCl by 20–80 µM $NHCl_2$ at pH 7. The experimental conditions were listed in Table S2 (Exp. Nos. 12–17). Symbols represent experimental data, and lines are model predictions. Error bar represent the ranges from experimental duplicates.

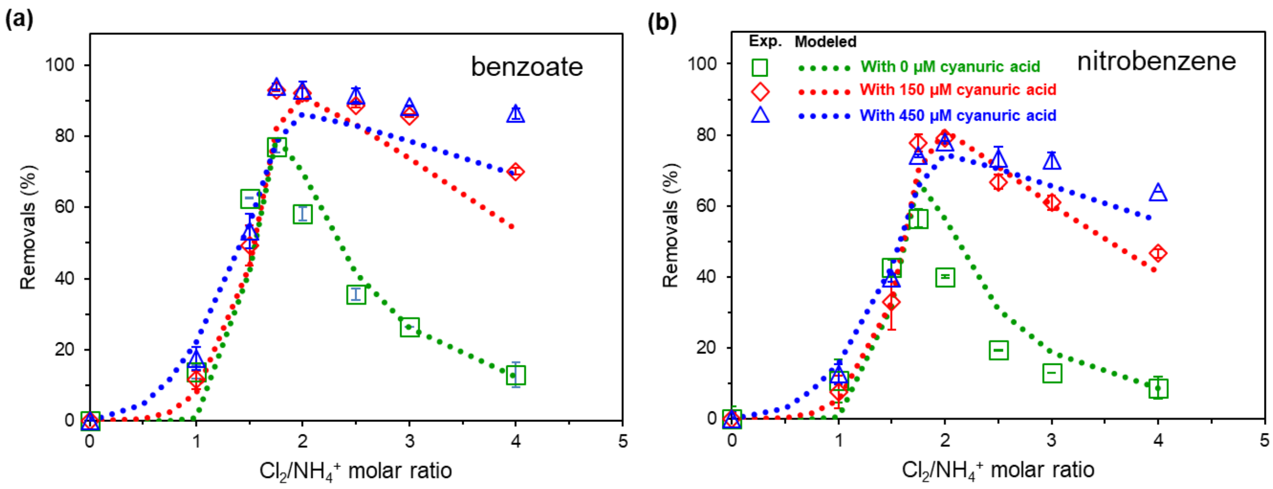

Figure S22. Experimental and modeled removals for (a) benzoate and (b) nitrobenzene during the chlorination of a mixture of 50 μM NH_4^+ and five micropollutants at 0.2 or 0.4 μM at pH 7 by 0–200 μM HOCl with or without cyanuric acid. Error bar represent the ranges from experimental duplicates.

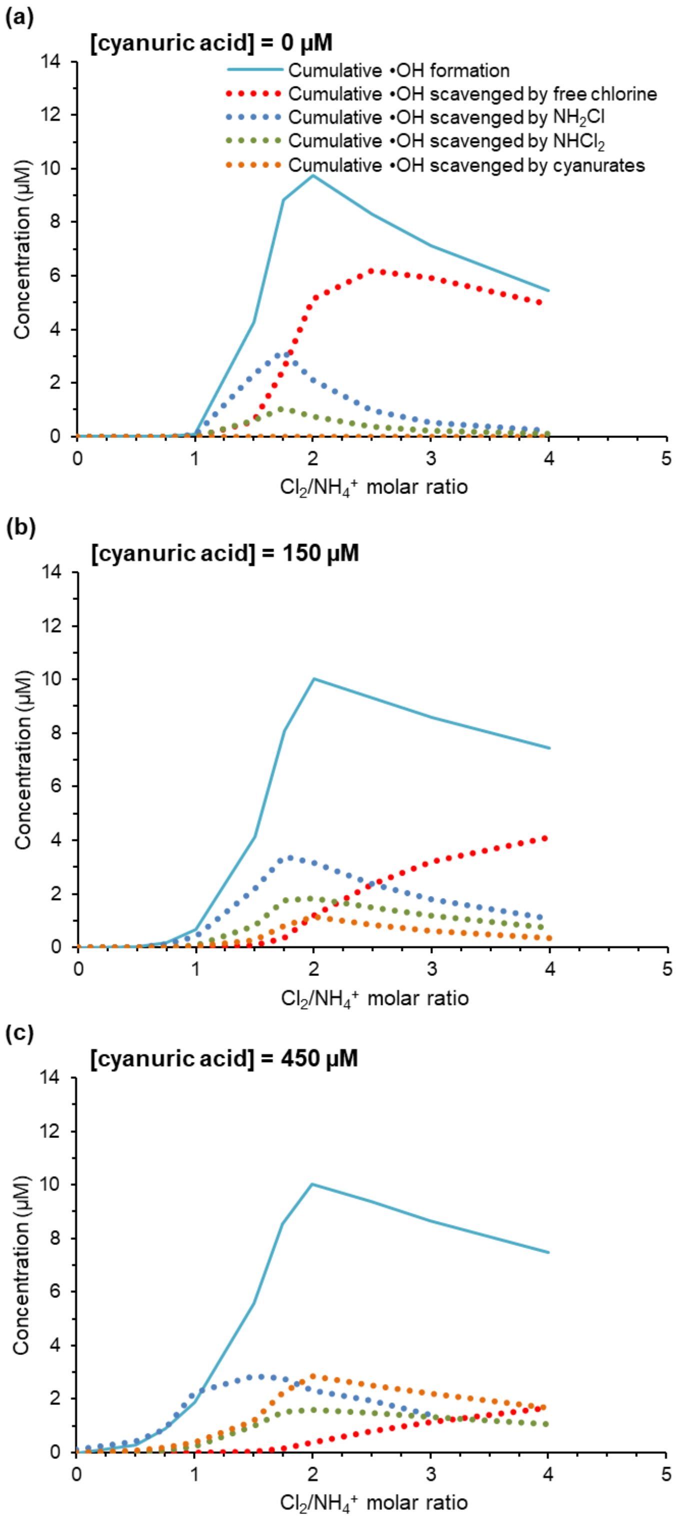

Figure S23. Modeled cumulative formation of •OH and cumulative •OH scavenged by free chlorine, NH_2Cl, $NHCl_2$, and (chlorinated) cyanurates at 60 minutes after treatment of a solution containing 50 µM NH_4^+ and five micropollutants (each at 0.2 or 0.4 µM) with 0–200 µM HOCl. Three cyanuric acid concentrations were tested: (a) 0 µM, (b) 150 µM, and (c) 450 µM, at pH 7.

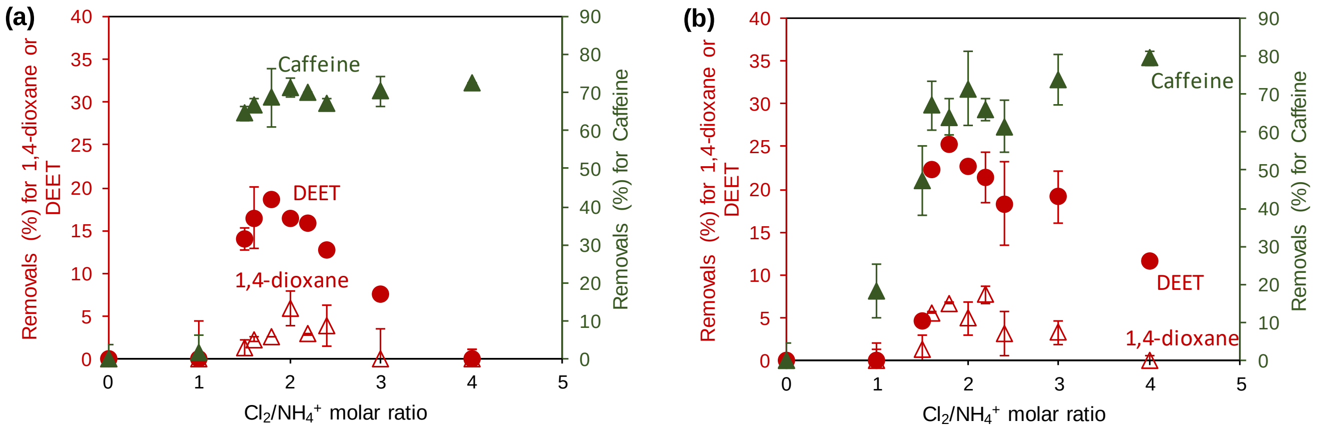

Figure S24. Micropollutant removal experiments at different Cl_2/NH_4^+ molar ratios in real pool water: (a) without cyanuric acid, and (b) with 60 µM cyanuric acid. A grab sample of pool water (which contained no cyanuric acid) was collected in northern Taiwan, with the following water quality parameters: pH 7.4; NH_4^+ < 0.01 mg/L-N; NO_2^- 0.005 mg/L-N; NO_3^- 4.08 mg/L-N; free chlorine 0.27 mg/L as Cl_2; total chlorine 0.28 mg/L as Cl_2; DOC 0.81 mg/L-C; total inorganic carbon 5.24 mg/L-C; UV_{254} absorbance 0.0142 cm^{-1}. Background free chlorine was first quenched with an equimolar amount of sodium thiosulfate as field sample were taken. To initiate the experiments, the three micropollutants were spiked into the pool water at 0.2 µM each, followed by the addition of 30 µM NH_4^+ and free chlorine at varying Cl_2/NH_4^+ molar ratios for 1 h. Error bar represents data range from experimental duplicates.

Text S8. Limitation of the ClNO vapor method for determining the rate constant of ClNO with HOCl/OCl$^-$ (k_{ClNO} for HOCl/OCl$^-$) and the estimations

Although the rate constant for the reaction between ClNO and cyanurates (H_3Cy/H_2Cy^-) was successfully determined using a competition kinetics approach, where ClNO vapor was generated and introduced into solutions containing both Cl-DMA (as a reference compound) and cyanuric acid at pH 7 (see Text S2), this method is not suitable for determining the rate constant for the reaction of ClNO with free chlorine species (HOCl/OCl$^-$). The limitation arises because ClNO vapor, upon dissolution in water, rapidly hydrolyzes to form NO_2^-, which subsequently reacts with HOCl. A previous study reported a hydrolysis rate constant for ClNO of 1.76×10^6 $M^{-1}s^{-1}$.[19] When we attempted to apply the same competition kinetics strategy using solutions containing 1 mM Cl-DMA and 1 mM HOCl in 20 mM phosphate buffer (pH 7), spiked with varying volumes (0–150 μL) of ClNO vapor, we consistently observed disproportionately large decreases in HOCl concentration compared to Cl-DMA. Moreover, the observed changes did not exhibit a clear linear relationship, further indicating the unsuitability of this method for quantifying ClNO reactivity with free chlorine.

To assess the potential impact of the ClNO–HOCl/OCl$^-$ reaction on NDMA formation, we used the Kintecus kinetic software to simulate the system described in this study. We evaluated how varying the rate constant for the reaction between ClNO and HOCl/OCl$^-$ (i.e., k_{ClNO} for HOCl/OCl$^-$) affects the cumulative product from the reaction of ClNO with Cl-DMA, which would be associated with NDMA formation concentration. Modeling were conducted using three k_{ClNO} for HOCl/OCl$^-$ at pH 7: (1) 6.8×10^6 $M^{-1}s^{-1}$, which is equal to k_{ClNO} for Cl-DMA, (2) 3.4×10^6 $M^{-1}s^{-1}$, which is half that of k_{ClNO} for Cl-DMA, and (c) 6.9×10^5 $M^{-1}s^{-1}$, which is equal to k_{ClNO} for H_3Cy/H_2Cy^- at pH 7. The results are presented in Figure S25. Under the conditions employed in Figure S25, the cumulative concentrations of products formed from the reaction of ClNO and Cl-DMA follow patterns similar to the observed NDMA formation concentration when k_{ClNO} for HOCl/OCl$^-$ was close to the k_{ClNO} for Cl-DMA (6.8×10^6 $M^{-1}s^{-1}$).

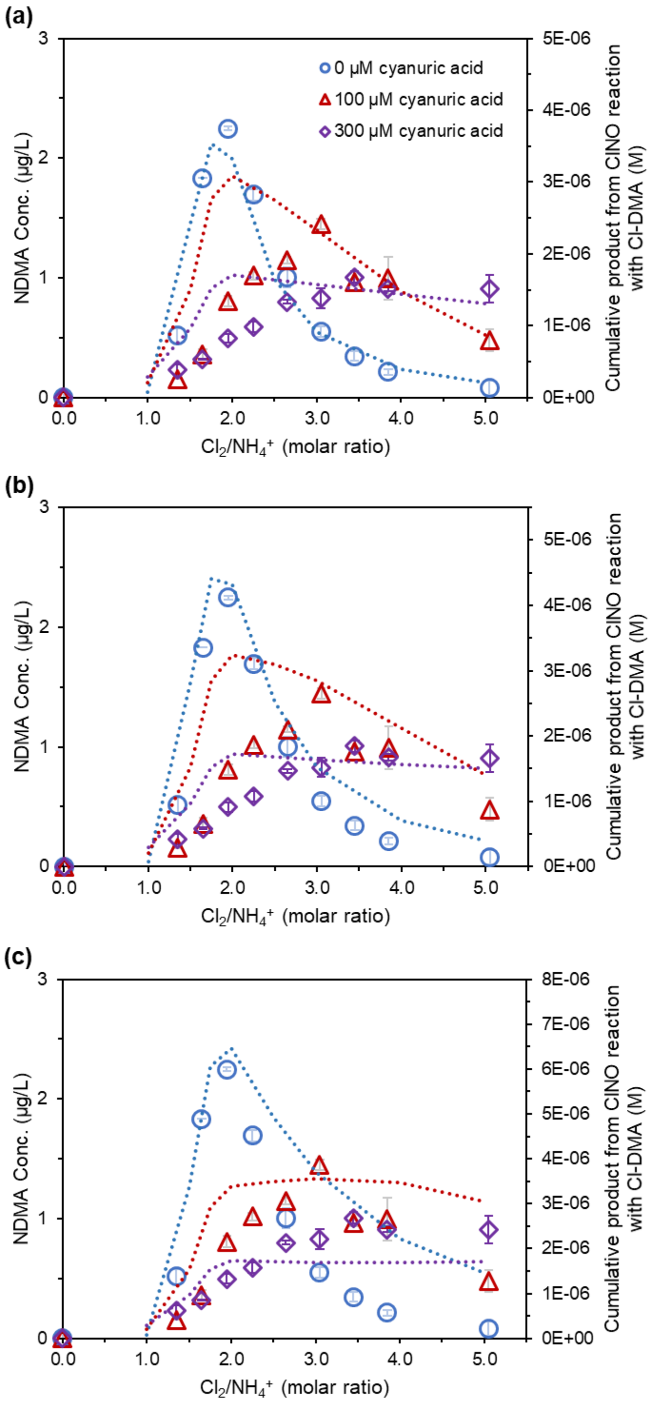

Figure S25. Comparison between experimental measured NDMA formation concentration (symbols; error bar represents data range from experimental duplicates) and modeled (dotted lines) cumulative product from the reaction of ClNO with Cl-DMA at 60 minutes after treatment of a solution containing 50 μM NH_4^+ and 7.5 μM Cl-DMA with 0–250 μM HOCl and with 0–300 μM cyanuric acid. The modeled cumulative product from the reaction of ClNO with Cl-DMA were obtained using three k for the reaction of ClNO with HOCl/OCl^-: (a) k_{ClNO} for HOCl/OCl^- = 6.8×10^6 $M^{-1}s^{-1}$, (b) k_{ClNO} for HOCl/OCl^- = 3.4×10^6 $M^{-1}s^{-1}$, (c) k_{ClNO} for HOCl/OCl^- = 6.9×10^5 $M^{-1}s^{-1}$.

Text S9. Micropollutant concentrations and NDMA formation potential in simulated chlorinated pool waters with or without cyanuric acid

We used a kinetic model to simulate the fate of micropollutants (DEET and caffeine, representative compounds frequently detected in pool waters[39]) and the cumulative yield of products from the reaction of ClNO with Cl-DMA, reflecting NDMA formation potential. The purpose of this simulation is to test whether the mechanistic implications derived from our lab experiments hold under continuous, pool-like input conditions, rather than to provide exact predictions of micropollutant or NDMA concentrations in real pools. The simulation considered a short-course pool (25 m × 20 m × 1 m; 500 m^3) with 400 swimmer visits over 12 h of operation, each releasing 250 mL of a "body fluid mixture" (200 mL sweat + 50 mL urine) based on previous studies.[40, 41]

The composition of sweat and urine was compiled from literature (Table S9),[39, 42, 43] including urea, lactate, ammonia, bicarbonate, and chloride in sweat, and urea, creatinine, ammonia, uric acid, DMA, and chloride in urine. Using these values, the body fluid mixture contained ~151 mM DOC (as C), 2.2 mM bicarbonate, 11.1 mM ammonium, and 0.32 mM DMA. Caffeine concentration was estimated as 0.6 µM using a urine-based caffeine:creatinine ratio reported in the literature,[39] and DEET release was assumed equal to caffeine for simplicity.

Assuming continuous excretion over 12 h, the release rates into the pool were calculated as follows: 7×10^{-10} M/s (as C) for DOC, 5.1×10^{-11} M/s (as N) for ammonium, 1.5×10^{-12} M/s for DMA, 1.0×10^{-11} M/s for bicarbonate, and 2.8×10^{-15} M/s for caffeine (and DEET).

For DOC: 400 swimming events × 0.25 L body fluid mixture / swimming event × 151.4 mmole-C/L / (25 m × 20 m × 1 m) / 12 h = 2.52 mmole-C/m^3/h = 7×10^{-10} M/s as C

For ammonia: 400 swimming events × 0.25 L body fluid mixture / swimming event × 11.1 mmole-N/L / (25 m × 20 m × 1 m) / 12 h = 0.19 mmole-N/m^3/h = 5.14×10^{-11} M/s as N

For DMA: 400 swimming events × 0.25 L body fluid mixture / swimming event × 0.32 mmole/L / (25 m × 20 m × 1 m) / 12 h = 0.62 mmole/m^3/h = 1.5×10^{-12} M/s

For $H_2CO_3^-$: 400 swimming events × 0.25 L body fluid mixture / swimming event × 2.2 mmole/L / (25 m × 20 m × 1 m) / 12 h = 0.037 mmole/m^3/h = 1.02×10^{-11} M/s

For caffeine: 400 swimming events × 0.25 L body fluid mixture / swimming event × 0.0006 mmole/L / (25 m × 20 m × 1 m) / 12 h = 1×10^{-5} mmole/m^3/h = 2.8×10^{-15} M/s

These parameters were incorporated into the kinetic model for a 12-h simulation with free chlorine (or free chlorine + chlorinated cyanurates) maintained at 1 or 2 mg/L as Cl_2. The model included the reactions listed in Table S4, supplemented by 12 additional reactions (Table S10) covering: continuous release of body-fluid constituents (Reactions S69–S74), chlorination of DMA to Cl-DMA (Reaction S77), radical scavenging by DOC and bicarbonate (Reactions S75–S76), and carbonate equilibria (Reactions S78–S81). Simulations were conducted at pH 7, assuming an initial DOC concentration of 0.5 mg/L-C and an initial HCO_3^- concentration of 1 mM, representative of typical concentrations in pristine pool water.

Simulation results are shown in Figure S26. After 400 swimming events, DEET and caffeine concentrations reached ~0.12 nM, and DOC concentration increased from 0.5 to 0.86 mg/L. The DOC increase of 0.36 mg/L-C is consistent with a previous field study reporting a ~0.2 mg/L-C increase after ~500 swimming visitors.[44] In pools without cyanuric acid (Scenario 2), caffeine was almost completely removed, while DEET removal was limited (~0.5%). The limited removal of DEET is attributable to •OH scavenging by free chlorine,

bicarbonate, and organic matter, while the near-quantitative removal of caffeine reflects the contribution of •ClO generated from HOCl/OCl^- reacting with radicals. Adding 100–400 µM cyanuric acid slightly enhanced DEET removal (~4%) while maintaining complete caffeine removal (Scenarios 3–5). The cumulative ClNO–Cl-DMA product increased with cyanuric acid from 0 to 100 µM, then declined as cyanuric acid further increased to 400 µM. Simulations with 2 mg/L free chlorine (or free chlorine + chlorinated cyanurates) gave comparable trends. Overall, the modeling results matched the trends observed in our experiments.

Table S9. Composition of body fluid mixtures

Components	Concentration range (average)	Reference
Human sweat		
Lactate	5–40 (22.5) mM	[42]
Urea	4–12 (8) mM	[42]
Ammonia	1–8 (4.5) mM	[42]
Bicarbonate	0.5–5 (2.75) mM	[42]
Chloride	10–90 (50) mM	[42]
Urine		
Urea	170–600 (385) mM	[39]
Creatinine	5–20 (12.5) mM	[39]
Ammonia	15–60 (37.5) mM	[39]
Uric acid	2–6 (4) mM	[39]
Chloride	50–200 (125) mM	[39]
Body fluid mixture		
Urea	83.4 mM	Calculated using 80% human sweat + 20% urine
Creatinine	2.5 mM	
Lactate	18 mM	
Ammonia	11.1 mM	
Bicarbonate	2.2 mM	
Chloride	65 mM	
DMA	0.32 mM	[43]
Caffeine	0.6 µM	[39]
DEET	0.6 µM	

Table S10. Additional reactions incorporated into the kinetic model for simulating micropollutant removals and nitrosamine formation in chlorinated swimming pools with or without cyanuric acid.

No.	*k*	Unit for *k*	Reaction	Note	Ref.
S69			NH_3-source → NH_4^+	Rate = 5.14×10^{-11} M/s	
S70			DOC-source → DOC	Rate = 7×10^{-10} M/s	
S71			HCO_3^--source → HCO_3^-	Rate = 1.02×10^{-11} M/s	
S72			DMA-source → DMA	Rate = 1.5×10^{-12} M/s	
S73			Caffeine-source → Caffeine	Rate = 2.8×10^{-15} M/s	
S74			DEET-source → DEET	Rate = 2.8×10^{-15} M/s	
S75	1.36×10^{8}	$M^{-1}s^{-1}$	•OH + DOC → products		45
S76	8.5×10^{6}	$M^{-1}s^{-1}$	•OH + HCO_3^- → products		6
S77	6.1×10^{7}	$M^{-1}s^{-1}$	HOCl + DMA → Cl-DMA		46
S78	4.3×10^{3}	$M^{-1}s^{-1}$	H_2CO_3 → H^+ + HCO_3^-	pK_a = 6.37	
S79	1×10^{10}	$M^{-1}s^{-1}$	H^+ + HCO_3^- → H_2CO_3	Assumed	
S80	4.8×10^{-1}	$M^{-1}s^{-1}$	HCO_3^- → H^+ + CO_3^{2-}	pK_a = 10.3	
S81	1×10^{10}	$M^{-1}s^{-1}$	H^+ + CO_3^{2-} → HCO_3^-	Assumed	

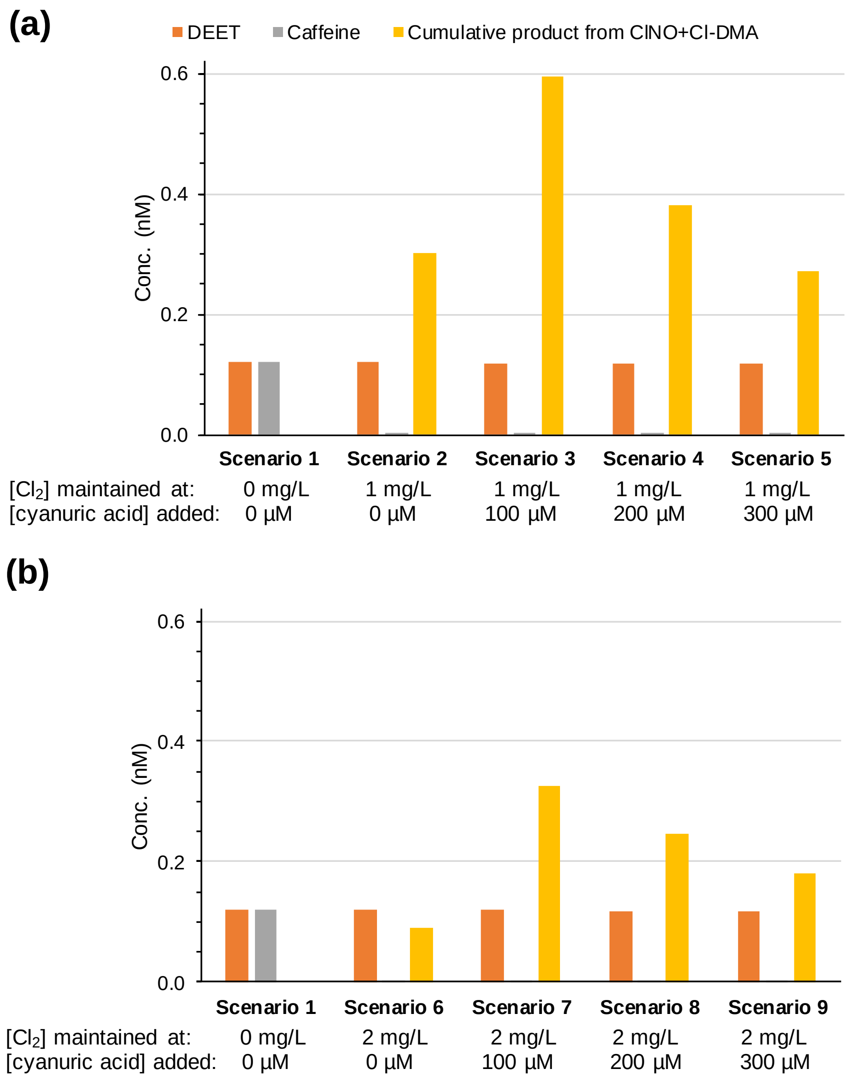

Figure S26. Simulated concentrations of micropollutants and cumulative product from the reaction of ClNO with Cl-DMA in chlorinated pool water under continuous swimmer inputs, with or without cyanuric acid. Kinetic simulations considered a short-course pool (25 m × 20 m × 1 m) with 400 swimmer visits over 12 h, releasing 250 mL of body fluid mixture per event. Free chlorine (or free chlorine + chlorinated cyanurates) was maintained at (a) 1 mg/L as Cl_2 and (2) 2 mg/L as Cl_2. Simulations were performed at pH 7 with an initial DOC of 0.5 mg/L-C and HCO_3^- of 1 mM.

References

1. Zhang, Z.; Chuang, Y. H.; Huang, N.; Mitch, W. A., Predicting the contribution of chloramines to contaminant decay during ultraviolet/hydrogen peroxide advanced oxidation process treatment for potable reuse. *Environ. Sci. Technol.* **2019,** *53*, (8), 4416-4425.
2. Schurter, L. M.; Bachelor, P. P.; Margerum, D. W., Nonmetal Redox Kinetics: Mono-, Di-, and Trichloramine Reactions with Cyanide Ion. *Environ. Sci. Technol.* **1995,** *29*, (4), 1127-1134.
3. Chuang, Y. H.; Chen, T. Y.; Chou, C. S.; Chu, L. K.; Hou, C. Y.; Szczuka, A., Critical Role of Trichloramine Interaction with Dichloramine for N-Nitrosamine Formation during Breakpoint Chlorination. *Environ. Sci. Technol.* **2023,** *57*, (40), 15232-15242.
4. USEPA, Definition and Procedure for the Determination of the Method Detection Limit, Code of Federal Regulations Title 40, Part 136, Appendix B, Federal Register Vol. 49, No. 209. In 1984.
5. Patton, S.; Li, W.; Couch, K. D.; Mezyk, S. P.; Ishida, K. P.; Liu, H., Impact of the ultraviolet photolysis of monochloramine on 1,4-dioxane removal: New insights into potable water reuse. *Environ Sci Technol Lett* **2017,** *4*, (1), 26-30.
6. Buxton, G. V.; Greenstock, C. L.; Helman, W. P.; Ross, A. B., Critical Review of rate constants for reactions of hydrated electrons, hydrogen atoms and hydroxyl radicals (•OH/•O^-) in aqueous solution. *J Phys Chem Ref Data* **1988,** *17*, (2), 513-886.
7. Thomas, J., Rates of reaction of the hydroxyl radical. *T Faraday Soc* **1965,** *61*, 702-707.
8. Lei, Y.; Yu, Y.; Lei, X.; Liang, X.; Cheng, S.; Ouyang, G.; Yang, X., Assessing the Use of Probes and Quenchers for Understanding the Reactive Species in Advanced Oxidation Processes. *Environ. Sci. Technol.* **2023,** *57*, (13), 5433-5444.
9. Lu, S.; Shang, C.; Sun, B.; Xiang, Y., Dominant Dissolved Oxygen-Independent Pathway to Form Hydroxyl Radicals and the Generation of Reactive Chlorine and Nitrogen Species in Breakpoint Chlorination. *Environ. Sci. Technol.* **2023,** *57*, (1), 150-159.
10. Lei, Y.; Cheng, S. S.; Luo, N.; Yang, X.; An, T. C., Rate Constants and Mechanisms of the Reactions of •Cl^- and •Cl_2^- with Trace Organic Contaminants. *Environ. Sci. Technol.* **2019,** *53*, (19), 11170-11182.
11. The NIST database. NDRL/NIST Solution Kinetics Database (Web pages; https://kinetics.nist.gov/solution/). http://kinetics.nist.gov/solution/ (2025.01.01 accessed),
12. Sun, P. Z.; Lee, W. N.; Zhang, R. C.; Huang, C. H., Degradation of DEET and caffeine under UV/chlorine and simulated sunlight/chlorine conditions. *Environ. Sci. Technol.* **2016,** *50*, (24), 13265-13273.
13. Guo, K.; Wu, Z.; Shang, C.; Yao, B.; Hou, S.; Yang, X.; Song, W.; Fang, J., Radical chemistry and structural relationships of PPCP degradation by UV/chlorine treatment in simulated drinking water. *Environ. Sci. Technol.* **2017,** *51*, (18), 10431-10439.
14. Benitez, F. J.; Acero, J. L.; Real, F. J.; Roldan, G.; Rodriguez, E., Modeling the photodegradation of emerging contaminants in waters by UV radiation and UV/H2O2 system. *Journal of Environmental Science and Health, Part A* **2013,** *48*, (1), 120-128.
15. Song, W.; Cooper, W. J.; Peake, B. M.; Mezyk, S. P.; Nickelsen, M. G.; O'Shea, K. E., Free-radical-induced oxidative and reductive degradation of N,N ́ -diethyl-m-toluamide (DEET): Kinetic studies and degradation pathway. *Water Res.* **2009,** *43*, (3), 635-642.
16. Williams, D. L. H., Nitrosation Mechanisms. In *Advances in Physical Organic Chemistry*, Gold, V.; Bethell, D., Eds. Academic Press: 1983; Vol. 19, pp 381-428.
17. Clayden, J.; Greeves, N.; Warren, S., *Organic chemistry*. Oxford university press: 2012.
18. Lebl, R.; Cantillo, D.; Kappe, C. O., Continuous generation, in-line quantification and utilization of nitrosyl chloride in photonitrosation reactions. *React Chem Eng* **2019,** *4*, (4), 738-746.
19. Challis, B. C.; Shuker, D. E., The chemistry of nitroso-compounds. Part 14. Nitrosation of amines in aqueous solution by dissolved gaseous NOCl. *Journal of the Chemical Society, Perkin Transactions 2* **1979**, (7), 1020-1024.
20. Chuang, Y. H.; Shi, H. J., UV/chlorinated cyanurates as an emerging advanced oxidation process for drinking water and potable reuse treatments. *Water Res.* **2022,** *211*, 118075.

21. Stanbury, D. M., Kinetics and Equilibria Interconverting Aqueous Inorganic Chloramines: Errors and Corrections. *ACS ES&T Water* **2024,** *4*, (9), 3750-3757.
22. Jafvert, C. T.; Valentine, R. L., Reaction scheme for the chlorination of ammoniacal water. *Environ. Sci. Technol.* **1992,** *26*, (3), 577-586.
23. O'Brien, J. E.; Morris, J. C.; Butler, J. N., Equilibria in Aqueous Solutions of Chlorinated Isocyanurate. In *Equilibria in Aqueous Solutions of Chlorinated Isocyanurate. In Chemistry of Water Supply, Treatment, and Distribution.*, Ann Arbor Science Publishers Inc.: Ann Arbor, Mich., 1974.
24. Pinsky, M. L.; Hu, H.-C., Evaluation of the chloroisocyanurate hydrolysis constants. *Environ. Sci. Technol.* **1981,** *15*, (4), 423-430.
25. Matte, D.; Solastiouk, B.; Merlin, A.; Deglise, X., Étude cinétique de la N-chloration de l'acide cyanurique en phase aqueuse. *Canadian Journal of Chemistry* **1990,** *68*, (2), 307-313.
26. Jensen, J. N.; Johnson, J. D., Quantitation of interferences under equilibrium conditions with application to free chlorine analysis in the presence of organic chloramines. *Anal Chem* **1989,** *61*, (9), 991-994.
27. Bulman, D. M.; Mezyk, S. P.; Remucal, C. K., The impact of pH and irradiation wavelength on the production of reactive oxidants during chlorine photolysis. *Environ. Sci. Technol.* **2019,** *53*, (8), 4450-4459.
28. Song, D.; Liu, H.; Qiang, Z.; Qu, J., Determination of rapid chlorination rate constants by a stopped-flow spectrophotometric competition kinetics method. *Water Res.* **2014,** *55*, 126-132.
29. Wahman, D. G., Chlorinated cyanurates: Review of water chemistry and associated drinking water implications. *J. Am. Water Works Ass.* **2018,** *110*, (9), E1-E15.
30. Wojtowicz, J. A., Reevaluation of Chloroisocyanurate Hydrolysis Constants. *Journal of the Swimming Pool Spa Industry* **1996,** *2*, (2), 14.
31. Gardiner, J., Chloroisocyanurates in the treatment of swimming pool water. *Water Res.* **1973,** *7*, (6), 823-833.
32. Brady, A. P.; Sancier, K. M.; Sirine, G., Equilibria in Solutions of Cyanuric Acid and its Chlorinated Derivatives. *J Am Chem Soc* **1963,** *85*, (20), 3101-3104.
33. Albert, A., *The determination of ionization constants: a laboratory manual*. Springer Science & Business Media: 2012.
34. Lau, S. S.; Abraham, S. M.; Roberts, A. L., Chlorination Revisited: Does Cl– Serve as a Catalyst in the Chlorination of Phenols? *Environ. Sci. Technol.* **2016,** *50*, (24), 13291-13298.
35. Gallard, H.; Von Gunten, U., Chlorination of phenols: Kinetics and formation of chloroform. *Environ. Sci. Technol.* **2002,** *36*, (5), 884-890.
36. Jensen, J. N.; Johnson, J. D., Interferences by monochloramine and organic chloramines in free available chlorine methods. 2. N,N-Diethyl-p-phenylenediamine. *Environ. Sci. Technol.* **1990,** *24*, (7), 985-990.
37. Valentine, R. L.; Jafvert, C. T., General acid catalysis of monochloramine disproportionation. *Environ. Sci. Technol.* **1988,** *22*, (6), 691-696.
38. Vikesland, P. J.; Ozekin, K.; Valentine, R. L., Monochloramine decay in model and distribution system waters. *Water Res.* **2001,** *35*, (7), 1766-1776.
39. Bouatra, S.; Aziat, F.; Mandal, R.; Guo, A. C.; Wilson, M. R.; Knox, C.; Bjorndahl, T. C.; Krishnamurthy, R.; Saleem, F.; Liu, P.; Dame, Z. T.; Poelzer, J.; Huynh, J.; Yallou, F. S.; Psychogios, N.; Dong, E.; Bogumil, R.; Roehring, C.; Wishart, D. S., The Human Urine Metabolome. *PLOS ONE* **2013,** *8*, (9), e73076.
40. Judd, S. J.; Black, S. H., Disinfection by-product formation in swimming pool waters: a simple mass balance. *Water Res.* **2000,** *34*, (5), 1611-1619.
41. Jmaiff Blackstock, L. K.; Wang, W.; Vemula, S.; Jaeger, B. T.; Li, X.-F., Sweetened Swimming Pools and Hot Tubs. *Environ Sci Technol Lett* **2017,** *4*, (4), 149-153.
42. Baker, L. B.; Wolfe, A. S., Physiological mechanisms determining eccrine sweat composition. *European journal of applied physiology* **2020,** *120*, (4), 719-752.
43. Tricker, A. R.; Pfundstein, B.; Kälble, T.; Preussmann, R., Secondary amine precursors to nitrosamines in human saliva, gastric juice, blood, urine and faeces. *Carcinogenesis* **1992,** *13*, (4), 563-568.
44. Zwiener, C.; Richardson, S. D.; De Marini, D. M.; Grummt, T.; Glauner, T.; Frimmel, F. H., Drowning in Disinfection Byproducts? Assessing Swimming Pool Water. *Environ. Sci. Technol.* **2007,** *41*, (2), 363-372.

45. Lei, Y.; Lei, X.; Westerhoff, P.; Zhang, X.; Yang, X., Reactivity of Chlorine Radicals (Cl• and Cl2•–) with Dissolved Organic Matter and the Formation of Chlorinated Byproducts. *Environ. Sci. Technol.* **2021,** *55*, (1), 689-699.
46. Mitch, W. A.; Sedlak, D. L., Formation of N-nitrosodimethylamine (NDMA) from dimethylamine during chlorination. *Environ. Sci. Technol.* **2002,** *36*, (4), 588-595.
